# Multi-omics prediction of oat agronomic and seed nutritional traits across environments and in distantly related populations

**DOI:** 10.1007/s00122-021-03946-4

**Published:** 2021-10-13

**Authors:** Haixiao Hu, Malachy T. Campbell, Trevor H. Yeats, Xuying Zheng, Daniel E. Runcie, Giovanny Covarrubias-Pazaran, Corey Broeckling, Linxing Yao, Melanie Caffe-Treml, Lucía Gutiérrez, Kevin P. Smith, James Tanaka, Owen A. Hoekenga, Mark E. Sorrells, Michael A. Gore, Jean-Luc Jannink

**Affiliations:** 1 Plant Breeding and Genetics Section, School of Integrative Plant Science, Cornell University, Ithaca, NY 14853 USA; 2 Department of Plant Sciences, University of California Davis, Davis, CA 95616 USA; 3 International Maize and Wheat Improvement Center (CIMMYT), Km. 45, Carretera México-Veracruz, El Batán, 56130 Texcoco, Edo. de México México; 4 Proteomics and Metabolomics Facility, Colorado State University, C130 Microbiology, 2021 Campus Delivery, Fort Collins, CO 80521 USA; 5 Department of Agronomy, Horticulture & Plant Science, South Dakota State University, Brookings, SD 57007 USA; 6 Department of Agronomy, University of Wisconsin-Madison, Madison, WI 53706 USA; 7 Department of Agronomy & Plant Genetics, University of Minnesota, St. Paul, MN 55108 USA; 8 Cayuga Genetics Consulting Group LLC, Ithaca, NY 14850 USA; 9 USDA-ARS, Robert W. Holley Center for Agriculture and Health, Ithaca, NY 14853 USA

## Abstract

**Key message:**

Integration of multi-omics data improved prediction accuracies of oat agronomic and seed nutritional traits in multi-environment trials and distantly related populations in addition to the single-environment prediction.

**Abstract:**

Multi-omics prediction has been shown to be superior to genomic prediction with genome-wide DNA-based genetic markers (G) for predicting phenotypes. However, most of the existing studies were based on historical datasets from one environment; therefore, they were unable to evaluate the efficiency of multi-omics prediction in multi-environment trials and distantly related populations. To fill those gaps, we designed a systematic experiment to collect omics data and evaluate 17 traits in two oat breeding populations planted in single and multiple environments. In the single-environment trial, transcriptomic BLUP (T), metabolomic BLUP (M), G + T, G + M, and G + T + M models showed greater prediction accuracy than GBLUP for 5, 10, 11, 17, and 17 traits, respectively, and metabolites generally performed better than transcripts when combined with SNPs. In the multi-environment trial, multi-trait models with omics data outperformed both counterpart multi-trait GBLUP models and single-environment omics models, and the highest prediction accuracy was achieved when modeling genetic covariance as an unstructured covariance model. We also demonstrated that omics data can be used to prioritize loci from one population with omics data to improve genomic prediction in a distantly related population using a two-kernel linear model that accommodated both likely casual loci with large-effect and loci that explain little or no phenotypic variance. We propose that the two-kernel linear model is superior to most genomic prediction models that assume each variant is equally likely to affect the trait and can be used to improve prediction accuracy for any trait with prior knowledge of genetic architecture.

**Supplementary Information:**

The online version contains supplementary material available at 10.1007/s00122-021-03946-4.

## Introduction

Oat (Avena sativa L.) ranks sixth in world cereal production and has increasingly been consumed as a human food (USDA 2019). Oat has a high content of health-promoting compounds such as unsaturated fatty acids, dietary fiber, antioxidants, and vitamins, which makes it an interesting target for metabolomics studies from a human health and nutrition perspective (IMARC Group 2019). In addition, high-density genetic markers have been developed in oat (Bekele et al. 2018), a draft genome sequence has been released (PepsiCo 2020) and a high-quality and comprehensive seed transcriptome has been characterized (Hu et al. 2020). Furthermore, recent advances in high-throughput sequencing and metabolite profiling technologies enable quantification of gene expression and metabolite abundance for hundreds of samples with high precision and reasonable cost (Alseekh and Fernie 2018; Moll et al. 2014). All these advances in technology provides an opportunity to integrate different omics data and improve predictions for phenotypes of interest.

Several multi-omics prediction studies have been reported in cereal and animal species (Guo et al. 2016; Riedelsheimer et al. 2012; Schrag et al. 2018; Wang et al. 2019; Westhues et al. 2017; Xu et al. 2017; Xu et al. 2021; Ye et al. 2020; Zhao et al. 2015). These studies have shed light on the merits of multi-omics prediction over traditional genomic prediction and discussed useful statistical methods for integrating omics data. For instance, Xu et al. (2017) and Wang et al. (2019) suggested that best linear unbiased prediction was the most efficient method compared to other commonly used genomic prediction and non-linear machine learning methods. However, most of those studies were based on historical datasets with a limited number of metabolite features and each level of omics data was collected from different projects. Therefore, they were unable to evaluate the efficiency of multi-omics prediction in multi-environment trials and genetically distant populations. However, in plant breeding, multi-environment trials are important for assessing the performance of genotypes across environments and identifying well-adapted genotypes for a specific region (Burgueño et al. 2012; Mathew et al. 2018). In addition, prediction of breeding values of distantly related individuals are needed in many and perhaps the most promising applications of genomic selection in both plant and animal breeding programs (Lorenz and Smith 2015; Meuwissen 2009; Moghaddar et al. 2019).

To fill the knowledge gaps of multi-omics prediction in plant breeding, we designed a systematic experiment to collect omics data and evaluate eight agronomic and nine fatty acid traits (Table S1) in a core set of a worldwide oat collection (termed Diversity panel) planted in one environment and advanced breeding lines adapted to the upper Midwest region in the USA (termed Elite panel) planted in three environments. Our efforts included (i) comparing the accuracy of multi-omics prediction against genomic prediction in a single-environment trial; (ii) evaluating the efficiency of multi-omics prediction in multi-environment trials; and (iii) exploring the potential of using multi-omics data to predict distantly related individuals.

## Materials and methods

### The plant materials and experimental designs

The Diversity and Elite panels consisted of 378 and 252 lines (Table S2), respectively. The Diversity panel originally included 500 lines described by Carlson et al. (2019) that was a core set of worldwide collection of oat germplasm, and we further selected for lines with visible anther extrusion for the convenience of collecting developing seeds for RNA sequencing. The Diversity panel was planted at Ithaca, NY, and the Elite panel was planted at Madison, WI, Crookston, MN, and Brookings, SD, respectively. An augmented incomplete design was used for both panels. The Diversity panel included 18 blocks of 23 plots each, one common check across all blocks and six secondary checks replicated in three blocks each. The Elite panel included 12 blocks of 25 plots each, one common check across all blocks and two secondary checks replicated in six blocks each.

### Phenotype evaluation and analysis

Plant height was evaluated for five randomly selected plants in each plot after anthesis. Days to heading was defined by the days from seeding to heading in > 50% of total plants. 100 randomly selected seeds from each plot were dehulled with a hand dehuller for evaluation of hundred kernel weight, hundred hull weight and groat percentage. After dehulling, 50 randomly selected seeds were delivered to the Proteomics and Metabolomics Facility at Colorado State University for metabolite analysis, and the other 50 seeds were used for measuring seed length, width and height with an electronic micrometer. Fatty acids were identified and quantified with targeted GC-MS, then normalized to concentration (mg/g of oats) against the internal standard (C17:0) (details were described in the Supplemental Methods).

### Genotype analysis

Genotypic data of the two panels were downloaded from T3/oat (https://triticeaetoolbox.org/oat/). SNPs were filtered using the following criteria (i) minor allele frequency (MAF) > 2%; (ii) site missingness < 60%; and (iii) site heterozygosity < 10%. After initial SNP filtering, lines were selected if (i) call rate > 80% and (ii) heterozygosity < 10%. A total of 73,014 markers and 568 lines (368 for the diversity panel, 232 for the elite panel, 32 in common) met these criteria and were used for further analyses. Subsequently, missing genotypes were imputed using the linear regression method glmnet described by Chan et al. (2016). The imputed genotypic data was used for constructing a neighbor-joining tree based on Rogers’ distance using the ape package (Paradis et al. 2004), and the tree was visualized with the ggtree package (Yu 2020).

### Transcript profiling

RNAseq was based on developing seeds at 23 days after anthesis (DAA). The 23 DAA was chosen based on our pilot study (Hu et al. 2020) that showed 23 DAA had slightly higher correlation between transcript and metabolite abundance than other sampled seed developmental time points. Seed sample collection, RNA extraction, library construction procedures were described in details by Hu et al. (2020). Pooled libraries were sequenced using Illumina NextSeq500 with a 150 nt single-end run. The RNAseq reads quality trimming, transcript abundance quantification, and library size normalization followed Hu et al. (2020).

### Metabolite profiling and network analysis

Metabolite analysis was based on physiologically mature seeds because they have the highest level of health-promoting compounds and those compounds are stable at room temperature until germination. GC-MS non-targeted analysis and LC-MS phenyl–hexyl analysis were done at the Proteomics and Metabolomics Facility at Colorado State University. Details of chemical analysis, raw mass spectrometry data processing, metabolite annotation, and normalization were described in Supplemental Methods. The normalized metabolomics data were used for network analysis with the WGCNA package (Zhang and Horvath, 2005) following the tutorial at https://horvath.genetics.ucla.edu/html/CoexpressionNetwork/Rpackages/WGCNA/Tutorials/FemaleLiver-02-networkConstr-man.R. Module identification included the following steps: (i) Correlation network adjacency was calculated using the soft thresholding power 4, which was selected based on the scale independence chart as described in the WGCNA tutorial; (ii) To minimize effects of noise and spurious associations, we transformed the adjacency matrix into Topological Overlap Matrix (TOM), and calculated the corresponding dissimilarity (1-TOM); (iii) We then used hierarchical clustering to produce a hierarchical clustering tree of metabolite features based on TOM dissimilarity matrix with method = "average"; (iv) Modules were identified using the cutreeDynamic function with the following parameters: method = "hybrid", distM = dissTOM, deepSplit = 2, pamRespectsDendro = FALSE, minClusterSize = 20.

### Analysis of phenotypic traits, transcriptomic, and metabolic features

Phenotypic traits, transcriptomic and metabolic features were analyzed following a standard linear mixed model of an augmented design accounting for effects of check genotypes and blocks (Campbell et al. 2021a). For metabolites analysis, batch effect was also included in the model to account for batch variation. All statistical models were described in Supplemental Methods and fitted using the sommer package (Covarrubias-Pazaran 2016).

### Single-environment prediction

The additive genomic relationship matrix was calculated with the A.mat function implemented in the rrBLUP package (Endelman 2011), and relationship matrices for transcripts (TRM) and metabolites (MRM) were calculated with the following equations:

1 $${\text{TRM}} = \frac{1}{{N_{{\text{T}}} }} W_{{\text{T}}} W_{{\text{T}}}^{{\text{T}}} ,$$

2 $${\text{MRM}} = \frac{1}{{N_{{\text{M}}} }} W_{{\text{M}}} W_{{\text{M}}}^{{\text{T}}} ,$$

where *N*_T_ and *N*_M_ denoted the number of transcript and metabolite features, respectively, *W*_T_ and *W*_M_ are the feature matrices of transcripts and metabolites, and *W*_T_^T^ and *W*_M_^T^ are transpose of feature matrices.

GBLUP, Transcriptomic BLUP (T), metabolomic BLUP (M), G + T, G + M, and G + T + M models were fitted with the BGLR package (Pérez & De Los Campos, 2014). The equations used to implement G + T, G + M and G + T + M models are:

3 $$y = Xb + G\alpha + T\beta + \varepsilon ,$$

4 $$y = Xb + G\alpha + M\gamma + \varepsilon ,$$

5 $$y = Xb + G\alpha + T\beta + M\gamma + \varepsilon ,$$

where *y* is a vector of phenotypes, *X* is a design matrix relating the fixed effects to each genotype, *b* is a vector of fixed effects, *α*, *β* and *γ* are random effects of genome, transcriptome and metabolome, respectively; *G*, *T,* and *M* are design matrices allocating records to those random effects; *ε* is random residual effect.

In the Diversity panel, transcriptomics and metabolomics data were collected on the same plots as the phenotypic data and therefore non-genetic (i.e., microenvironmental) factors that affected both omics features and phenotypic traits may induce non-genetic correlations among traits. Therefore, we estimated prediction accuracy as $c\hat{o}r_{g} \left( {\sqrt {\hat{h}_{{\hat{u}}}^{2} } } \right)$ described by Runcie and Cheng (2019), and used a 50:50 training/testing split of the data to ensure that $c\hat{o}r_{g}$ could be estimated accurately in the testing partition. This cross-validation procedure was repeated for 50 times with different random partitions. To determine whether there was a significant difference in prediction accuracy between each omics model and the GBLUP model, we performed the Wilcoxon signed-rank test based on prediction accuracies across the 50 cross-validation runs for each pair of models. The Wilcoxon signed-rank test was also applied to multi-environment prediction and prediction of distantly related individuals in this study.

### Multi-environment prediction

The metabolomics data were also collected on the same plots as the phenotypic data for the Elite panel, which would bias prediction accuracy if directly using metabolites to predict target phenotypes from the same environment. Therefore, when predicting target phenotypes from one environment, we used metabolites from other two environments to make the metabolomic relationship matrix. For each trait, we fitted six multi-trait mixed models on G, M and G + M kernels with different genetic and residual covariance structures. A standard multi-trait linear mixed model was used, and the equation for the case of genomic SNPs is:

6 $$y = Xb + Zg + \varepsilon ,$$

where *y* = (*y*_1_’, *y*_2_’, *y*_3_’)’, *g* = (*g*_1_’, *g*_2_’, *g*_3_’)’, *ε* = (*ε*_1_’, *ε*_2_’, *ε*_3_’)’. *y*_1_, *y*_2_, and *y*_3_ are the column vectors of phenotypic data in each environment. g_1_, g_2_, and g_3_ are the column vectors of random genetic effects in each environment. *ε*_1_, *ε*_2_, and *ε*_3_ are the column vectors of random error terms associated with each environment. *X* and *Z* are design matrices relating the fixed and random effects to each genotype. Vectors containing the random effects in Eq. (6) are assumed to follow a multivariate normal distribution, centered at zero, and with covariance structure Cov(*g*, *g*’) = *G*_0_ $\otimes$ *K*, Cov(*ε*, *ε*’) = *I* $\otimes$ *R*_0_, and Cov(*g*, *ε*’) = 0, where *K* is the additive genomic relationship matrix, *I* is an identity matrix, $\otimes$ is the Kronecker product, *G*_0_ is a 3 × 3 genetic covariance matrix, *R*_0_ is a 3 × 3 residual covariance for the three locations. There are various covariance structures for *R*_0_ or *G*_0_ (Burgueño et al. 2012). In this study, six multi-trait models on three different kernels/combinations (G, M, G + M) with various genetic and residual covariance structure were used (codes and covariance structures of the six multi-trait mixed models were described in Table S3).

We applied a single-environment cross-validation method originally designed for genomic prediction described by Mathew et al. (2018) and extended it to multi-kernel omic prediction (illustrated in Fig. S1). To predict a phenotype in the first environment, we masked 20% of lines for cross-validation and used metabolites from the other two environments to construct the metabolomic relationship matrix. We then used multi-trait models treating phenotypes from all three environments as separate traits for model training but using only the phenotypic data of the masked lines from the first environment as the testing data. We further estimated prediction accuracy of the first environment as $r\left( {\hat{y},y} \right)/\sqrt {h^{2} }$ (Riedelsheimer et al. 2012), where *r*($\hat{y}$,y) is the Pearson correlation between the observed (y) and predicted ($\hat{y}$) phenotypic values and h^2^ is the heritability of the target trait. To predict the phenotype in the second and third environments, we masked 20% of lines (the same set of lines as those in the first environment) from the second and third environments, respectively, and calculated their prediction accuracies following the same procedure as that applied to the first environment. Finally, we averaged the three prediction accuracies across environments to represent the prediction accuracy of a single run. This procedure was repeated for 50 times with different random partitions.

### Prediction of distantly related individuals

Seed fatty acid concentrations were used as target traits for predicting distantly related individuals, which included two steps: likely causal loci prioritization in the Diversity panel (training population) and multiple-kernel prediction in the Elite panel (test population).

We first performed the WGCNA on all metabolite features in the Diversity panel (training population), and identified twenty-six network modules. Based on the metabolites annotation, we performed Fisher's exact test to identify a subset of network modules enriched with lipids and lipid-like molecules. We then performed hierarchical clustering (using correlation based dissimilarity matrix with method = "average") and GWAS on eigenvectors of the twenty-six network modules and PC1 of fatty acids. GWAS was performed based on the linear mixed model (Yu et al. 2006) implemented in the GWAS function of the rrBLUP package (Endelman 2011) with the following parameters: K = GRM (additive genomic relationship matrix), n.PC = 2, min.MAF = 0.02, n.core = 4 (Campbell et al. 2021a). Based on these analyses, we found that a 'darkred' module enriched with lipids and lipid-like molecules, clustered together with PC1 of fatty acids, and its eigenvector had a QTL co-located with the major-effect QTL of fatty acids on chromosome 6A. We finally prioritized 140 markers including significant markers and the markers in LD with them based on the GWAS peak on chromosome 6A identified from the 'darkred' module. A LD threshold of *r*^2^ = 0.1 was used as it is frequently recommended for SNP pruning (Kawakami et al. 2014).

The prioritized markers and all rest markers were used to construct two genomic relationship kernels in the Elite panel (test population) and perform a multiple-kernel prediction. The two genomic relationship matrices were calculated with the A.mat function implemented in the rrBLUP package (Endelman 2011). Genomic predictions with GBLUP and BayesB models were used as references to compare with the two-kernel linear model. The fivefold cross-validation was used to estimate prediction accuracies for all models and the prediction accuracy was estimated as $r\left( {\hat{y},y} \right)/\sqrt {h^{2} }$ (Riedelsheimer et al. 2012). This cross-validation procedure was repeated for 50 times with different random partitions.

## Results

**Fig. 1:**
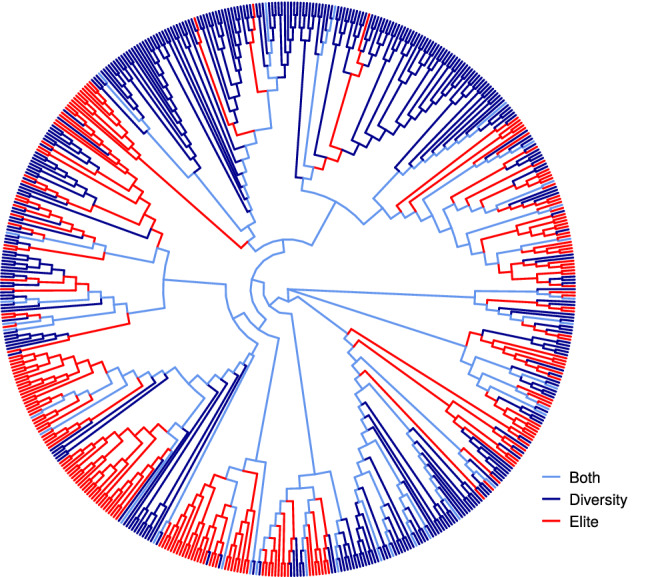
Neighbor-joining tree of 568 oat lines in the Diversity and Elite panels. Different panels are shown in different colors (darkblue, Diversity panel; red, Elite panel, light blue, lines in common)

After filtering out lines with low-quality genetic markers, the Diversity and Elite panels consisted of 368 and 232 lines (Table S2), respectively, with 32 lines in common. A reconstructed phylogenetic tree revealed that most of clusters were primarily comprised of either the Diversity or the Elite panel members, although a couple of clusters had approximately equal representation from both sets (Fig. 1). This is consistent with our prior knowledge about different origins of the two panels (Carlson et al., 2019; Campbell et al., 2021b).

### Single-environment prediction in the Diversity panel

**Fig. 2:**
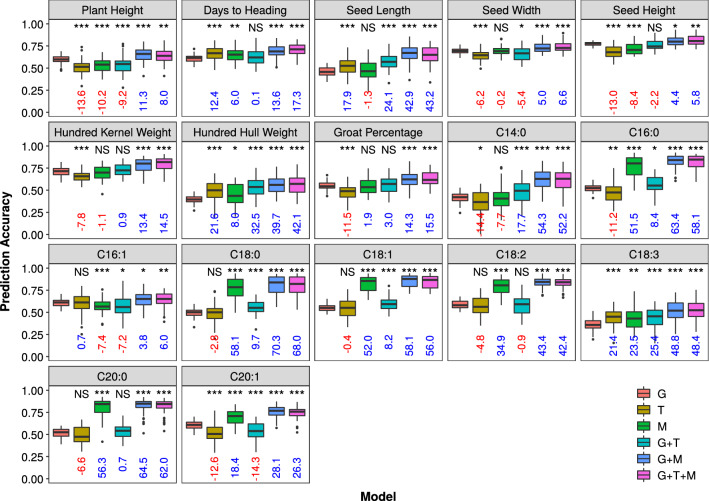
Distribution of prediction accuracy of the 17 phenotypic traits in the Diversity panel across 50 re-sampling runs. For each trait, boxplots with different colors represent prediction models. Medians of percent change in prediction accuracy of omics models relative to GBLUP are indicated below each box in blue if positive and in red if negative. The Wilcoxon Signed Rank was applied to test difference in prediction accuracy between each omics model and the GBLUP model, and significance levels are indicated above each box. *** = significant at *P* < 0.001, ** = significant at *P* < 0.01, * = significant at *P* < 0.05, NS = not significant. G = genomic BLUP, T = transcriptomic BLUP, M = metabolomic BLUP

Using GBLUP (G) as a baseline, there were 5, 10, 11, 17, and 17 traits out of the 17 total traits with improved prediction accuracy from transcriptomic BLUP (T), metabolomic BLUP (M), G + T, G + M, and G + T + M models, respectively (Fig. 2, Table S4). Percent change in prediction accuracy over GBLUP ranged from 0.1% (Days to Heading, G + T model) to 70.3% (C18:0, G + M model) with a median of 21.5%, and most of differences in prediction accuracy between omics models and GBLUP are statistically significant. Because GBLUP does not allow for large-effect or zero-effect genetic markers, we also compared BayesB with the multi-omics models, and found BayesB showed similar results to GBLUP (Fig. S2).

To evaluate whether transcriptomic and metabolomic features equally contribute to improved prediction accuracy or if one is more important than the other, we compared multi-omics prediction models with T and M kernels added in different orders. By adding kernels in their order along the central dogma of molecular biology, median prediction accuracy changes from G to G + T models and from G + T to G + T + M models across all traits ranged from − 11.6 to 35.8% (median = 3.2%) and 6.5–55.6% (median = 16.3%), respectively (Fig. S3). In contrast, when adding the M kernel first (G + M model) then followed by the T kernel (G + T + M model), percent changes in prediction accuracy ranged from 2.5 to 67.3% (median = 41.7%) and − 3.3 to 3.5% (median = − 0.03%), respectively (Fig. S4). These results indicated that seed metabolites generally contributed more than transcripts to improving prediction accuracy of both agronomic and seed nutritional traits when combined with SNPs.

In addition to playing important roles in improving prediction accuracy when combined with other kernels, metabolites alone from mature seeds (M model) greatly outperformed SNPs (G model) and transcripts (T model) in predicting fatty acids (except C16:1, Fig. 2). To understand why metabolites are better predictors for fatty acid traits, we used the Weighted Gene Co-expression Network Analysis (WGCNA, Zhang and Horvath 2005) that accommodated both annotated and unannotated compounds and used metabolites annotations (Table S5) to elucidate their biological functions. The WGCNA was designed to construct gene/metabolite co-expression networks, and a co-expression module (network module) may reflect a true biological pathway (Langfelder and Horvath 2008). We identified twenty-six network modules and found that eight of them were enriched with lipids and lipid-like molecules (Table S6), which included 33.0% of total identified seed metabolite compounds.

### Multi-environment prediction in the Elite panel

Beyond single-environment prediction, omics data might also have merit in predicting multi-environment trials, which has not yet been investigated to our knowledge. Here we used SNPs and metabolites for analyzing the multi-environment trials in the Elite panel, because transcript profiling from a single developmental time point showed limited value for improving prediction accuracy in addition to being very labor-intensive. We focused on prediction of lines that have been evaluated in some but not in target environments (termed CV2 by Burgueño et al. 2012). To this aim, we applied a single-environment cross-validation method (Mathew et al. 2018) (Fig. S1). Briefly, to predict a phenotype in the first environment, we masked 20% of lines for cross-validation and used metabolites from the other two environments to construct metabolomic relationship matrices to minimize the influence of non-genetic effects on prediction accuracy. We then used multi-trait models treating phenotypes from all three environments as separate traits for model training but using only the phenotype data of the masked lines from the first environment as the testing data. This procedure was repeated for the second and third environments and prediction accuracies were averaged across the three environments for each run.

**Fig. 3:**
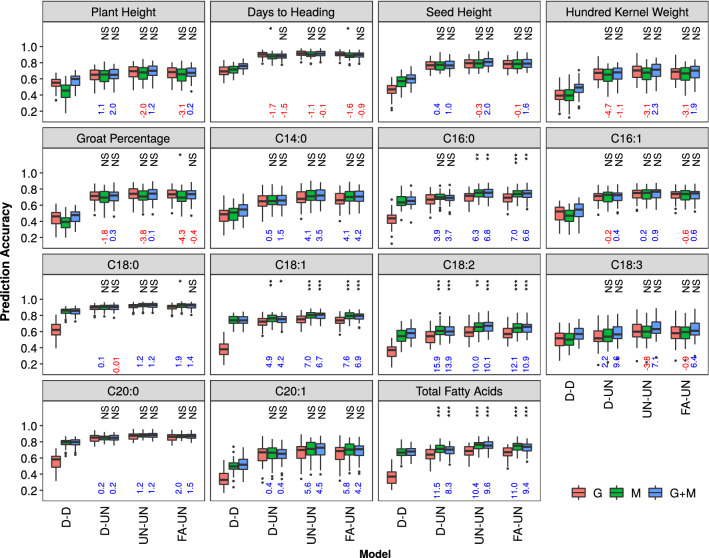
Distribution of prediction accuracy of the 15 phenotypic traits in the Elite panel across 50 re-sampling runs estimated by multi-trait models. For each trait, boxplots with different colors represent models. Medians of percent change in prediction accuracy of M and G + M models relative to the G model are indicated below each box in blue if positive and in red if negative. For each model, the uppercase letters before and after the hyphen represent genetic and residual covariance structures: D = diagonal, UN = unstructured, FA = factor-analytic. The Wilcoxon Signed Rank was applied to test difference in prediction accuracy between each omics model and the GBLUP model, and significance levels are indicated above each box. *** = significant at *P* < 0.001, ** = significant at *P* < 0.01, * = significant at *P* < 0.05, NS = not significant

Multi-environment predictions were performed using six multi-trait models (Table S3) on three different kernels/combinations (G, M, G + M) with various genetic and residual covariance structures (Fig. 3 showed prediction accuracies of D-D, D-UN, UN-UN and FA-UN models; Fig. S5 showed prediction accuracies of UN-D and FA-D models; the uppercase letters before and after the hyphen represent genetic and residual covariance structures; D = diagonal, UN = unstructured, FA = factor-analytic). The diagonal heterogeneous covariance structure (D-D) corresponds to a single-environment model without borrowing information from other environments. The question that we explored was whether multi-omics models (M and G + M) could improve prediction accuracy compared to corresponding multi-trait models based on SNPs alone (G model). To answer this question, within each of the five multi-trait models (the D-D model was excluded), we compared percent change in prediction accuracy of M and G + M models relative to the G model. We found the M model outperformed the G model for all seed fatty acid traits except C16:1 and C18:3, with an increase in prediction accuracy ranging from 0.1 to 15.9%. However, the G + M model outperformed the G model for all traits except days to heading, with an increase in prediction accuracy over the G model ranging from 0.1 to 13.9%. For four fatty acids traits (C16:0, C18:1, C18:2, and total fatty acids), there was a significant difference in prediction accuracy between the multi-trait models (the D-D model was not included) and the corresponding GBLUP model at the significance level of 0.01 for both M and G + M kernels. These results confirmed the value of using multi-omics data in the multi-environment prediction.

In genomic prediction, Burgueño et al. (2012) had shown that different genetic and residual covariance structures in the multi-trait models boosted predictive power in across-environment prediction differently. Mathew et al. (2018) further showed that different residual covariance structures impacted on genomic prediction ability in multi-environment trials and therefore residual covariances across multiple environments couldn’t be neglected. To understand the impact of different genetic and residual covariance structures on prediction accuracy in the context of multi-omics prediction, we compared the performance of different multi-trait models using the prediction accuracy from GBLUP in the single-environment model (D-D) as a baseline. We found that all multi-trait models outperformed their counterpart single-environment models (Fig. 3, Figs. S6-8). The multi-trait models generally performed better when modeling the genetic covariance as unstructured (UN) or as factor-analytic (FA) than modeling genetic covariance as a diagonal structure (D). The highest prediction accuracy was achieved by either UN-D (UN and D represent genetic and residual covariance structures, respectively) or UN-UN models, although FA-D and FA-UN models provided very similar results.

### Using multi-omics data to improve genomic prediction in distantly related populations

Although multi-omics data showed superiority over SNPs to predict phenotypes in both single and multi-environment trials, currently transcript and metabolite profiling is more expensive than SNP genotyping, which would limit their applications in plant breeding. Here we hypothesized that omics data from well characterized populations can be used to prioritize likely causal loci and improve performance of genomic prediction models in distantly related populations. Seed fatty acid concentrations were used as target traits to test the hypothesis because their genetic architectures have been well characterized (Carlson et al. 2019; Campbell et al. 2021a) and lipid biosynthetic pathways are known to be highly conserved in higher plants (de Abreu et al. 2018).

To explore this scientific question, we first attempted to prioritize likely causal loci from the Diversity panel (training population) based on the eight network modules enriched with lipids and lipid-like molecules (Table S6). Among the eight network modules, only one ('darkred') strongly correlated with fatty acids (Fig. S9). We then performed hierarchical clustering and GWAS on eigenvectors of all the 26 network modules and PC1 of fatty acids. The eigenvector of the 'darkred' module was clustered together with PC1 of fatty acids (Fig. S10) and had significant GWAS hits on chromosome 6A (Fig. S11), which co-located with the fatty acids major-effect QTL (*QTL-6A*, Fig. S12). However, the *QTL-6A* was not detected from other network modules. We further prioritized 140 markers including significant markers and the markers in LD with them based on the 'darkred' module GWAS hits on chromosome 6A.

**Fig. 4:**
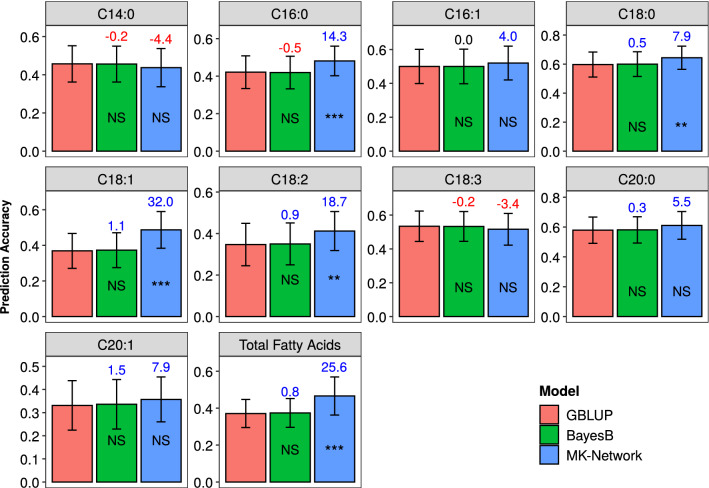
Prediction accuracy of the 10 fatty acid traits in the Elite panel estimated by GBLUP, BayesB and two-kernel BLUP models across 50 re-sampling runs. For each trait, barplots with different colors represent models. Means of percent change in prediction accuracy of all other models relative to GBLUP are indicated above each bar (in blue if positive, in red if negative, and in black if zero). MK-Network = network-based multiple-kernel prediction. The Wilcoxon Signed Rank was applied to test difference in prediction accuracy between other models and the GBLUP model, and significance levels are indicated on each bar. *** = significant at *P* < 0.001, ** = significant at *P* < 0.01, * = significant at *P* < 0.05, NS = not significant

The primary use of locus prioritization is to split markers in the test population into two sets for a multi-kernel model prediction, in which the two genomic relationship kernels were constructed from the two marker sets. We termed our method multi-kernel network-based prediction (MK-Network) and found it improved prediction accuracy over GBLUP and BayesB for all fatty acid traits except C14:0 and C18:3 (Fig. 4) in the Elite panel (test population). For the eight fatty acids traits with improved prediction accuracy in the MK-Network model, the percent change of mean prediction accuracy over GBLUP across 50 cross-validation runs ranged from 4.0% (C16:1) to 32.0% (C18:1) with a mean of 14.5%. For five of the eight fatty acids traits, there was significant difference in prediction accuracy between the MK-Network model and GBLUP model at the significance level of 0.01. All the eight fatty acids traits showed clear peaks for GWAS hits at the *QTL-6A*, although only five of them were significant at FDR < 0.05 (Fig. S12). In contrast, C14:0 had significant GWAS hits on chromosomes 6D and 7A, and C18:3 showed a complex genetic architecture with several visible GWAS peaks on several different chromosomes.

## Discussion

### Roles of transcripts and metabolites in the single-environment prediction

In the single-environment prediction, we found that transcripts showed limited value for improving prediction accuracy either by themselves alone or by combining with SNPs. Other researchers (Guo et al. 2016; Westhues et al. 2017; Xu et al. 2017) also reported that prediction abilities of transcripts were either lower than or close to GBLUP for some traits in their studies and were affected by many other factors. The poor predictive performance of transcripts in existing studies might be explained because they were collected from a single developmental time point and subject to dynamic changes in later unsampled developmental stages or because transcripts and SNPs tend to capture similar genetic signals for predicted traits (Guo et al. 2016).

Metabolite abundance from seedling tissues (Riedelsheimer et al. 2012; Schrag et al. 2018; Westhues et al. 2017), flag leaves (Zhao et al. 2015), and mature seeds (Guo et al. 2016, Xu et al. 2017) were reported not superior to SNPs for predicting hybrid performance and agronomic traits. In this study, we found that metabolites alone (M model) from mature seeds showed mixed results for predicting agronomic traits (Fig. 2), and only significantly better over GBLUP for two traits (Days to Heading and Hundred Hull Weight). One reason for the relatively low performance of metabolite compounds in predicting agronomic and other complex traits across studies could be that development of the agronomic traits and accumulation of the compounds analyzed in existing studies occurred either at different times or in different tissues and therefore the target traits and predictor compounds are quite distant from each other in terms of biological pathways.

However, we found that seed metabolites greatly outperformed SNPs in predicting fatty acids in our study (Fig. 2). In contrast to agronomic and other complex traits, these compounds and fatty acids were synthesized in the same tissue, a large proportion of them directly or indirectly connected with fatty acids through biochemical pathways (Tables S4-5); and different pathways relevant to lipids were likely influenced by overlapping gene sets. Therefore, they should be able to capture more genetic covariance (including both additive and non-additive) with fatty acids than SNPs fitted in an additive model. This hypothesis was partially supported by our results that combining G model and M model (G + M model) significantly improved prediction accuracies than using the G model alone for all the 17 traits (Fig. 2, Table S7) and by findings of Guo et al. (2016) that adding metabolites to saturated SNP densities still led to significant increases in predictive abilities. However, the increase of prediction accuracy with the omics models cannot completely rule out possibilities of non-genetic contributions, for example, cellular microenvironment that affected both target traits of fatty acids and predictor compounds. To provide a better understanding on how the omics models improve prediction accuracy, further research is needed to dissect contributions to the improved prediction accuracy into additive genetic, non-additive genetic and non-genetic components.

### Application of omics data in the multi-environment prediction

In the multi-environment prediction, we observed that for predicting agronomic traits, the M model performed similarly to the G model (i.e., M ~ G, Fig. 3), however, the M model outperformed G model for predicting fatty acids traits (i.e. M > G). This pattern is very similar to that observed in the single-environment prediction, and therefore could be interpreted similarly. Both analyses indicated that when predicting traits very distantly connected or unconnected through biological pathways, metabolites functioned similarly to DNA-based genetic markers (i.e., we need to trace back to the DNA along the central dogma); however, when predicting relevant traits that directly/indirectly connected through biological pathways, metabolites could capture more genetic covariance with the target traits than DNA-based genetic markers, because they shared more similarities in temporal and spatial expression.

In addition, we observed that all multi-trait models outperformed their counterpart single-environment models (Fig. 3, Figs. S6-8), and the multi-trait models generally performed better when modeling the genetic covariance as unstructured (UN) or as factor-analytic (FA) than modeling genetic covariance as a diagonal structure (D). This indicated that the genetic covariance between environments played an important role in the multi-omics prediction models. These findings agree with recent genomic prediction studies (Malosetti et al. 2016; Mathew et al. 2018; Montesinos-López et al. 2016) that UN covariance structure improved prediction accuracy compared to the models with diagonal homogeneous or heterogeneous covariances. Overall, we concluded that considering genetic and non-genetic covariances is useful to improve prediction accuracy of multi-environment models using multi-omics data.

### The genetic basis of predicting distantly related individuals and advantages of the two-kernel linear model

In the prediction of distantly related individuals, the universal QTL of fatty acids (*QTL-6A*, Figs. S12-13) and similar LD relationships (Fig. S14) with the surrounding loci between the Diversity and Elite panels promoted the success of our likely causal loci prioritization. The network-based prioritization strategy takes advantages of pleiotropy, in which one or a few genes influence both target traits and other metabolites from related network modules. In the 'darkred' module, 23 of 32 metabolites showed clear peaks at the *QTL-6A*, although only five of them were significant at FDR < 0.05 (Fig. S15). This indicated that *QTL-6A* was likely a causal locus and influenced both fatty acids and the 'darkred' module. The relationships between fatty acids and the 'darkred' module are expected to be conserved between populations. However, we were unable to test this because there is currently no robust method to map all untargeted metabolites from one panel to another and quantify them accurately.

Most genomic prediction methods assume that each variant is equally likely to affect the trait (MacLeod et al. 2016). There are certain loci that explain more phenotypic variance and they should be placed in different kernels than loci that explain little or no variance. However, the other kernel is still needed because we may unintentionally exclude important loci based on prior biological knowledge alone, for example, a prior GWAS might not identify all possible causal loci. There are many loci that have small effects, through whatever pathway, whether it is through trans effects as hypothesized in the omnigenic model (Liu et al. 2019) or through much more indirect effects like competition for photosynthates or impact on fitness (Price et al. 2018). Li et al. (2018) found that excluding those small-effect loci could not further improve prediction accuracy compared to GBLUP with all SNPs. Therefore, a two-kernel linear model that accommodates both likely casual loci and loci with minimal to no effect should be used to improve prediction accuracy for any traits with prior knowledge of genetic architecture.

## Author contribution statement

JJ, MAG, and MES designed the research. HH analyzed the data. HH, MTC, MAG, and JJ wrote the manuscript. DER, GC, OAH and MES advised HH on data analysis. HH, THY, XZ, MC, LC, KPS, and JT performed experiments. CB and LY performed metabolite analysis. All co-authors were involved in editing the manuscript.

## Supplementary Information

Below is the link to the electronic supplementary material.

Supplementary file1 (PDF 2950 kb)

## Data Availability

All the phenotypic data and omics data are available on CyVerse Data Commons (Hu 2021). Scripts for running all the multi-omics prediction analyses are available at https://github.com/hh622/Oat_Multi-omics_Prediction.
