# Supplementary material for: Multi-omics prediction of oat agronomic and seed nutritional traits across environments and in distantly related populations
Source: Theor Appl Genet. 2021 Oct 13;134(12):4043–54. doi: 10.1007/s00122-021-03946-4 (PMC8580906; doi:10.1007/s00122-021-03946-4)
Supplement: Supplementary file 1 — Supplementary file1 (PDF 2950 kb) [file 122_2021_3946_MOESM1_ESM.pdf]

Supplemental Information

Multi-omics prediction of oat agronomic and seed nutritional traits across environments and in distantly related populations

Haixiao Hu, Malachy T. Campbell, Trevor H. Yeats, Xuying Zheng, Daniel E. Runcie, Giovanny Covarrubias-Pazaran, Corey Broeckling, Linxing Yao, Melanie Caffe-Treml, Lucía Gutiérrez, Kevin P. Smith, James Tanka, Owen A. Hoekenga, Mark E. Sorrells, Michael A. Gore, and Jean-Luc Jannink

The Supplemental Information includes:

- Supplemental Methods
- Supplemental Figures
- Supplemental Tables

Supplemental Methods

Metabolic profiling

Extraction. Samples were homogenized and extracted using a biphasic extraction method to separate polar and non-polar compounds. Samples were homogenized using the Next Advance Bullet Blender system (Troy, NY) in which the intact oats were first crushed in 5-mL polypropylene tubes before the addition of stainless steel beads followed by repetitive concussive forces that grind the oats into a fine powder. The homogenized tissue (100 mg) was weighed into 2-mL glass vials for automated biphasic extraction using a GERSTEL Dual-head MPS fitted with 2.5 mL and 100 µL syringes (GERSTEL; Linthicum, MD).

The extraction solvent methanol/methyl-tert-butyl-ether (MTBE) (1.155 mL, 2:1, v/v) was added to each sample followed by vortexing for 30 min at 800 rpm on MPS. To induce biphasic separation, 640 µL of water was added to each vial followed by an additional 15 minutes of vortexing at 800 rpm. The samples were then centrifuged at 3,000 *g* for 15 min at 4°C and put back onto the MPS system. 60 µL of the upper organic layer from each sample was pooled to create a QC mix. 600 µL of the upper organic layer was transferred to clean 2-mL glass vials. To remove the remaining organic layer in the extraction vial that could not be selectively removed by the syringe needle, 600 µL of MTBE was added back to the original vial slowly as to not disturb the biphasic boundary and then 600 µL was subsequently removed and pooled with the rest of the organic layer. This addition and removal processes was repeated three times to ensure all organic phase was recovered. 600 µL of methanol/acetonitrile (1:1, v/v) was added to the remaining aqueous layer in the extraction vial and vortexed for 3 min to precipitate proteins and excess glycans followed by centrifugation at 3,000 *g* for 15 min at 4°C. 60 µL of aqueous extract was then removed and pooled to create an aqueous layer QC mix before 1.7 mL was transferred to new 2 mL glass vials. Both the organic and aqueous extracts were dried under nitrogen gas overnight. Aqueous fractions were re-suspended in 1.5 mL of 50% methanol in water (v/v) for GC-MS and LC-MS untargeted analysis. Organic fractions were re-suspended in 0.7 mL of methanol/toluene (1:1, v/v) for FAME analysis.

LC-MS Phenyl-Hexyl Analysis. 5 µL of aqueous extract was injected onto a Waters Acquity UPLC system with a pooled QC injection after every 5 sample injections. The metabolites were separated using a Waters Acquity UPLC CSH Phenyl Hexyl column (1.7 µm, 1.0 x 100 mm), using a gradient from solvent A (2mM ammonium formate with

0.1% formic acid) to solvent B (acetonitrile with 0.1% formic acid). The gradient started with 100% A, held at 100% A for 1 min, ramped to 98% B over 12 min, held at 98% B for 3 min, and then returned to starting conditions over 0.05 min and allowed to re-equilibrate for 3.95 min, with a 200 µL/min constant flow rate. The column and samples were held at 65°C and 6°C, respectively. The column eluent was analyzed with a Waters Xevo G2 TOF-MS with an electrospray source in positive mode (cone voltage =30 V), scanning 50-2000 m/z at 0.2 s per scan, alternating between MS (6 V collision energy) and MSE mode (15-30 V ramp). Calibration was performed using sodium iodide with 1 ppm mass accuracy. The capillary voltage was held at 2200 V, source temp at 150°C, and nitrogen desolvation temperature at 350°C with a flow rate of 800 L/hr.

GC-MS Non-targeted Analysis. 40 µL of the aqueous extract was dried under nitrogen, re-suspended in 50 µL of pyridine containing 25 mg/mL of methoxyamine hydrochloride, incubated at 60°C for 1 h, sonicated for 10 min, and incubated for an additional 1 h at 60°C. Next, 50 µL of N- methyl-N-trimethylsilyltrifluoroacetamide with 1% trimethylchlorosilane (MSTFA + 1% TMCS, Thermo Scientific) was added and samples were incubated at 60°C for 45 min, briefly centrifuged, cooled to room temperature, and 100 µL of the supernatant was transferred to a 150-µL glass insert in a GC-MS autosampler vial. Metabolites were detected using a Trace 1310 GC coupled to a Thermo ISQ mass spectrometer (Thermo Scientific). Samples were injected in a 1:10 split ratio. Separation occurred using a 30 m TG-5MS column (Thermo Scientific, 0.25 mm i.d., 0.25 µm film thickness) with a 1.2 mL/min helium gas flow rate, and the program consisted of 80˚C for 30 s, a ramp of 15°C/min to 330°C, and an 8 min hold. Masses between 50-650 m/z were scanned at 5 scans/s after electron impact ionization.

FAME preparation. Samples were re-suspended in 0.7 mL of methanol/toluene (1:1, v/v) and 70 µL was transferred to a 2-mL glass vial and solvent was completely removed by nitrogen evaporation at ambient temperature. To the dry sample, 100 µL of toluene containing 2.5 mg/mL of internal standard, glyceryl triheptadecanoate, and 200 µL of 3 N methanolic HCl were added. The mixture was incubated at 60 °C for 1 hour. Then 0.5 mL of hexane and 0.7 mL of water were added to the cooled sample. After a brief vortex, the sample was centrifuged at 3000 *g* for 5 min at 4°C. After centrifugation, the upper hexane layer was diluted twice with 100% hexane and 100 µL transferred to glass inserts for analysis.

FAME GCMS. Upper hexane layer containing FAME (1 µL) was injected into a TG-WAXMS column (30 m x 0.25 mm x 0.25 µm, Thermo) on a Trace1310 GC (Thermo) coupled to a Thermo ISQ-LT MS. The injector temperature was 260 °C, and split ratio was 15:1. A constant flow rate of the carrier gas (Helium) was controlled at 1.2 mL/min. The initial oven temperature

was 200°C and held for 1 min, then increased to 260 °C at 10 °C/min and held for 3 min. Detection was completed under electron impact mode, with a scan range of 50-650 m/z and scan rate 5 scans/s. Transfer line and source temperature were both at 250°C. Data processing was completed with Chromeleon 7 software (ThermoFisher). QC sample were injected after every 6 samples. Standard curves of C14:0, C16:1, C16:0, C18:0, C18:1, C18:2, C18:3, C20:0, and C20:1 were acquired. The peak area of each fatty acid in the standard curve samples was normalized to that of internal standard (C17:0) and plotted against the concentrations of standards, so that a linear fit equation was obtained to be used to extrapolate the concentration of each fatty acid in actual samples.

Data Processing. For each sample, raw data files were converted to .cdf format, and matrix of molecular features as defined by retention time and mass (m/z) was generated using XCMS software in R (Smith et al., 2006) for feature detection and alignment. The matchedFilter algorithm was used for GC-MS data, and the centWave algorithm for LC-MS data. Features were grouped using RAMClustR (Broeckling et al., 2014), with normalization set to 'none'. GC-MS spectra were annotated by matching unknown spectra to the GOLM metabolome retention indexed spectral library (Kopka et al., 2005), using retention times plotted vs the GOLM retention index to increase confidence in the spectral match. Searching was accomplished using the RAMSearch program (Broeckling et al., 2016).

LC-MS data were first annotated by searching against an in-house spectra and retention time database using RAMSearch. RAMClustR was used to call the findMain (Jaeger et al., 2017) function from the interpretMSSpectrum package to infer the molecular weight of each LC-MS compound and annotate the mass signals. The complete MS spectrum and a trunctated MSE spectrum were written to a .mat format for import to MSFinder (Tsugawa et al., 2016). The MSE spectrum was truncated to only include masses with values less than the inferred M plus its isotopes, and the .mat file precursor ion is set to the M+H ion for the findMain inferred M value. These .mat spectra were analyzed to determine the most probable molecular formula and structure. MSFinder was also used to perform a spectral search against the MassBank database. All results were imported into R and a collective annotation is derived with prioritization of RAMSearch > MSFinder mssearch > MSFinder structure > MSFinder formula > findMain M. Annotation confidence is reported as described (Sumner et al., 2007). All R work was performed using R 3.3.1 (R Core Team, 2017).

Normalization. The original GC-MS and LC-MS spectra abundances were normalized using the Log Transformation method as described by Li et al. (2016). Then, to further

control batch effect, the log transformed spectra abundances were fitting in a linear mixed model including batch number as a covariate (described on the next page).

Analysis of phenotypic traits, transcriptomic and metabolic features

Linear mixed model for analyzing agronomic traits in a single-environment trial:

$$y = \mu + Check + Block + New{:}Entry + e \qquad (Eq.1)$$

Linear mixed model for analyzing metabolites in a single-environment trial:

$$y = \mu + Check + Block + Batch + New{:}Entry + e \qquad (Eq.2)$$

Linear mixed model for analyzing metabolites in multi-environment trials:

$$y = \mu + Check + Loc + Loc{:}Block + Batch + New{:}Entry + Loc{:}New{:}Entry + e \qquad (Eq.3)$$

where Check is a fixed effect for check varieties; Loc is a random location effect; Block and Batch are random effects to account for field blocks and injection batch for GCMS/LCMS; Loc:Block is Block effect nested within location; New is an indicator variable where 0 indicates a check variety and 1 indicates a test entry, and is nested within entry; Loc:New:Entry is random effect of location by entry interaction. The terms μ and e represent the overall mean and the vector of residuals, respectively. The above models were fitted using the sommer package in R (Covarrubias-Pazaran, 2016). Best linear unbiased predictors (BLUPs) were calculated for each variety for downstream analysis.

References

Broeckling, CD, Afsar, FA, Neumann, S., Ben-Hur, A., Prenni, JE. (2014). RAMClust: A novel feature clustering method enables spectral-matching-based annotation for metabolomics data. Analytical Chemistry. DOI: 10.1021/ac501530d.

Broeckling CD, Ganna A, Layer M, Brown K, Sutton B, Ingelsson E, Peers G, Prenni JE. Enabling Efficient and Confident Annotation of LC-MS Metabolomics Data through MS1 Spectrum and Time Prediction. Anal Chem. 2016 Sep 20;88(18):9226-34. doi: 10.1021/acs.analchem.6b02479. Epub 2016 Sep 8. PubMed PMID: 27560453.

Covarrubias-Pazaran, G. (2016). Genome-Assisted prediction of quantitative traits using the r package sommer. PLoS ONE, 11(6), 1–15. https://doi.org/10.1371/journal.pone.0156744

Jaeger C, Méret M, Schmitt CA, Lisec J. Compound annotation in liquid chromatography/high -resolution mass spectrometry based metabolomics: robust adduct ion determination as a prerequisite to structure prediction in electrospray ionization mass spectra. Rapid Commun Mass Spectrom. 2017 Aug 15;31(15):1261- 1266. doi: 10.1002 /rcm.7905. PubMed PMID: 28499062.

Kopka, J., Schauer, N., Krueger, S., Birkemeyer, C., Usadel, B., Bergmuller, E., Dormann, P., Weckwerth, W., Gibon, Y., Stitt, M., Willmitzer, L., Fernie, A.R. and Steinhauser, D. (2005) GMD@CSB.DB: the Golm Metabolome Database, Bioinformatics, 21, 1635-1638

Li B. , Tang J., Yang Q., Cui X., Li S., Chen S., Cao Q., Xue W., Chen N., Zhu F. Performance evaluation and online realization of data-driven normalization methods used in LC/MS based untargeted metabolomics analysis. Sci. Rep.2016; 6:38881

R Core Team (2017). R: A language and environment for statistical computing. R Foundation for Statistical Computing, Vienna, Austria. URL https://www.R-project.org/.

Smith CA, Want EJ, O'Maille G, Abagyan R, Siuzdak G (2006) XCMS: Processing Mass Spectrometry Data for Metabolite Profiling Using Nonlinear Peak Alignment, Matching, and Identification. Analytical Chemistry 78: 779-787.

Sumner LW, Amberg A, Barrett D, Beale MH, Beger R, et al. (2007). Proposed minimum reporting standards for chemical analysis. Metabolomics 3:211–21

Tsugawa H, Kind T, Nakabayashi R, Yukihira D, Tanaka W, Cajka T, Saito K, Fiehn O, Arita M. Hydrogen Rearrangement Rules: Computational MS/MS Fragmentation and Structure Elucidation Using MS-FINDER Software. Anal Chem. 2016 Aug 16;88(16):7946-58. doi: 10.1021/acs.analchem.6b00770. Epub 2016 Aug 4. PubMed PMID: 27419259.

Supplemental Figures

Fig. S1 a scheme of single environment cross validation using metabolites (M model) or SNPs and metabolites (G+M model) for multi-environment prediction.

Fig. S2 Distribution of prediction accuracy of the 17 phenotypic traits in the Diversity panel across 50 re-sampling runs estimated by multi-omics and BayesB models.

Fig. S3 Prediction accuracy changes from G to G+T models and from G+T to G+T+M models of the 17 phenotypic traits in the Diversity panel across 50 re-sampling runs.

Fig. S4 Prediction accuracy changes from G to G+M models and from G+M to G+T+M models of the 17 phenotypic traits in the Diversity panel across 50 re-sampling runs.

Fig. S5 Distribution of prediction accuracy of the 15 phenotypic traits in the Elite panel across 50 re-sampling runs estimated by multi-trait models of D-D, UN-D and FA-D.

Fig. S6 Percentage changes in prediction accuracy estimated from multi-environment GBLUP models over single-environmental GBLUP for the 15 phenotypic traits in the Elite panel

Fig. S7 Percentage changes in prediction accuracy estimated from multi-environment metabolite BLUP (M) models over single-environmental GBLUP for the 15 phenotypic traits in the Elite panel

Fig. S8 Percentage changes in prediction accuracy estimated from multi-environment genomic and metabolomic BLUP (G+M) models over single-environmental GBLUP for the 15 phenotypic traits in the Elite panel.

Fig. S9 Correlation between eigenvector of network modules and fatty acid traits.

Fig. S10 Hierarchical clustering dendrogram of the network eigenvectors and PC1 of the nine fatty acids.

Fig. S11 Manhattan plots of eigenvectors of twenty-six network modules identified by WGCNA and PC1 of fatty acids in the Diversity panel.

Fig. S12 Manhattan plots of fatty acids traits in the Diversity panel.

Fig. S13 Manhattan plots of fatty acids traits in the Elite panel.

Fig. S14 LD relationships of locus QTL-6A with the surrounding loci in the Diversity and Elite panels.

Fig. S15 Manhattan plots of metabolites in the darkred module identified by WGCNA in the Diversity panel.

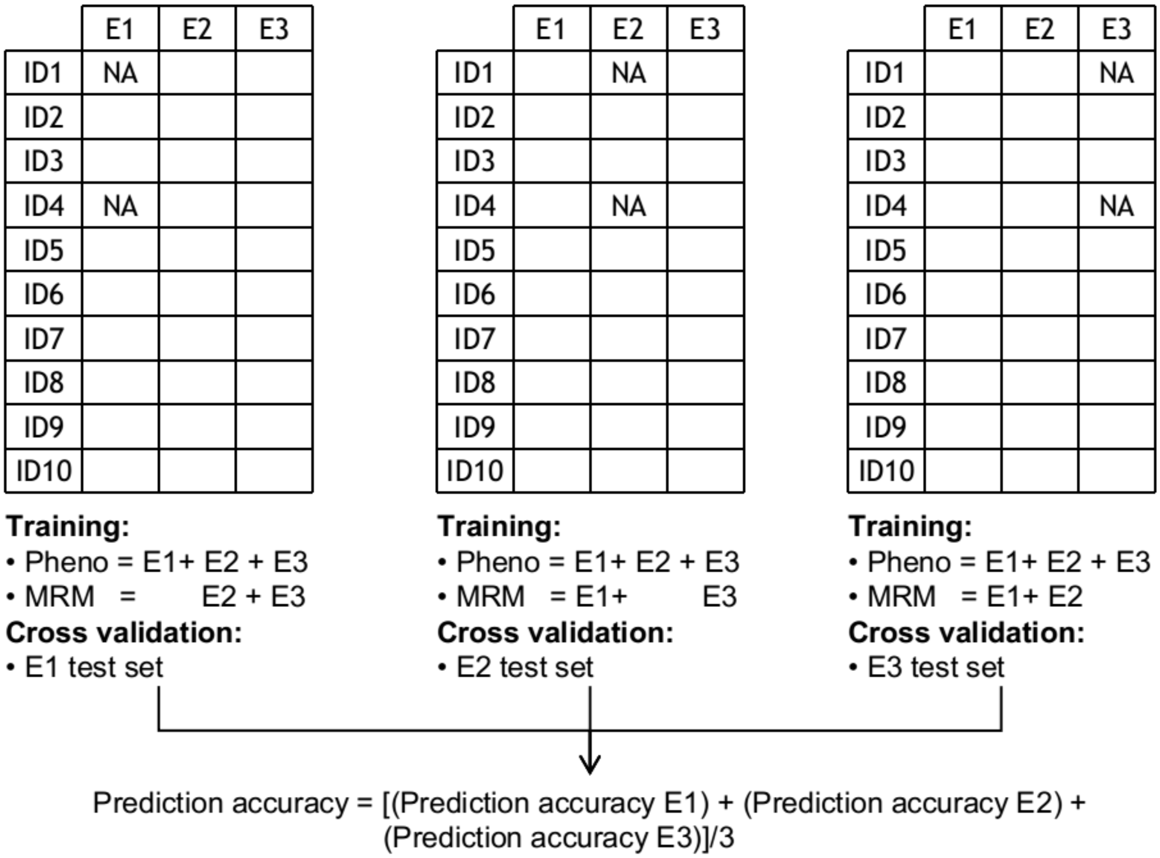

Fig. S1 a scheme of single environment cross validation using metabolites (M model) or SNPs and metabolites (G+M model) for multi-environment prediction. E1= Environment 1, E2=Environment 2, E3=Environment 3, Pheno=Phenotype, MRM=metabolomic relationship matrix.

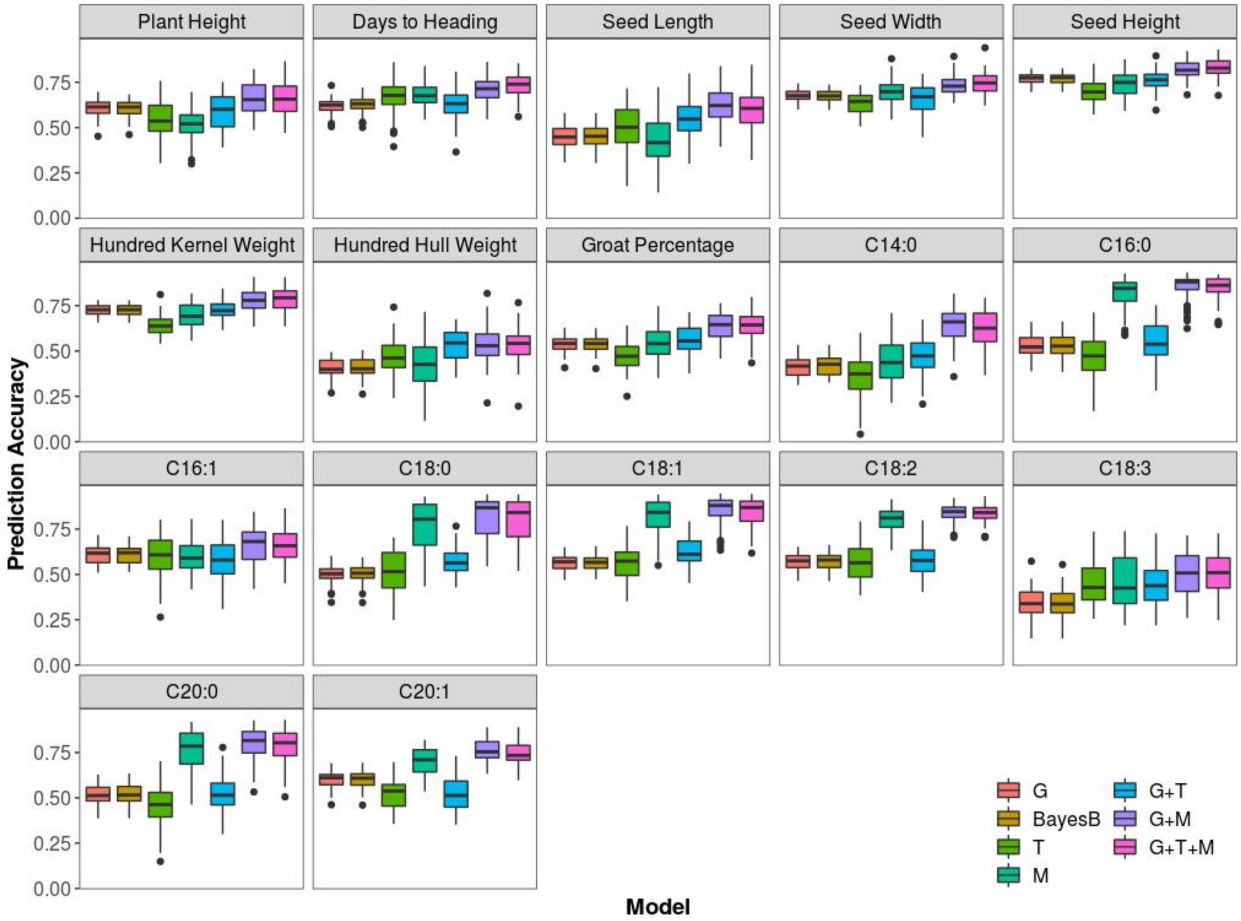

Fig. S2 Distribution of prediction accuracy of the 17 phenotypic traits in the Diversity panel across 50 re-sampling runs estimated by multi-omics and BayesB models. For each trait, boxplots with different colors represented prediction results estimated by different models. G = genomic BLUP, T = transcriptomic BLUP, M = metabolomic BLUP.

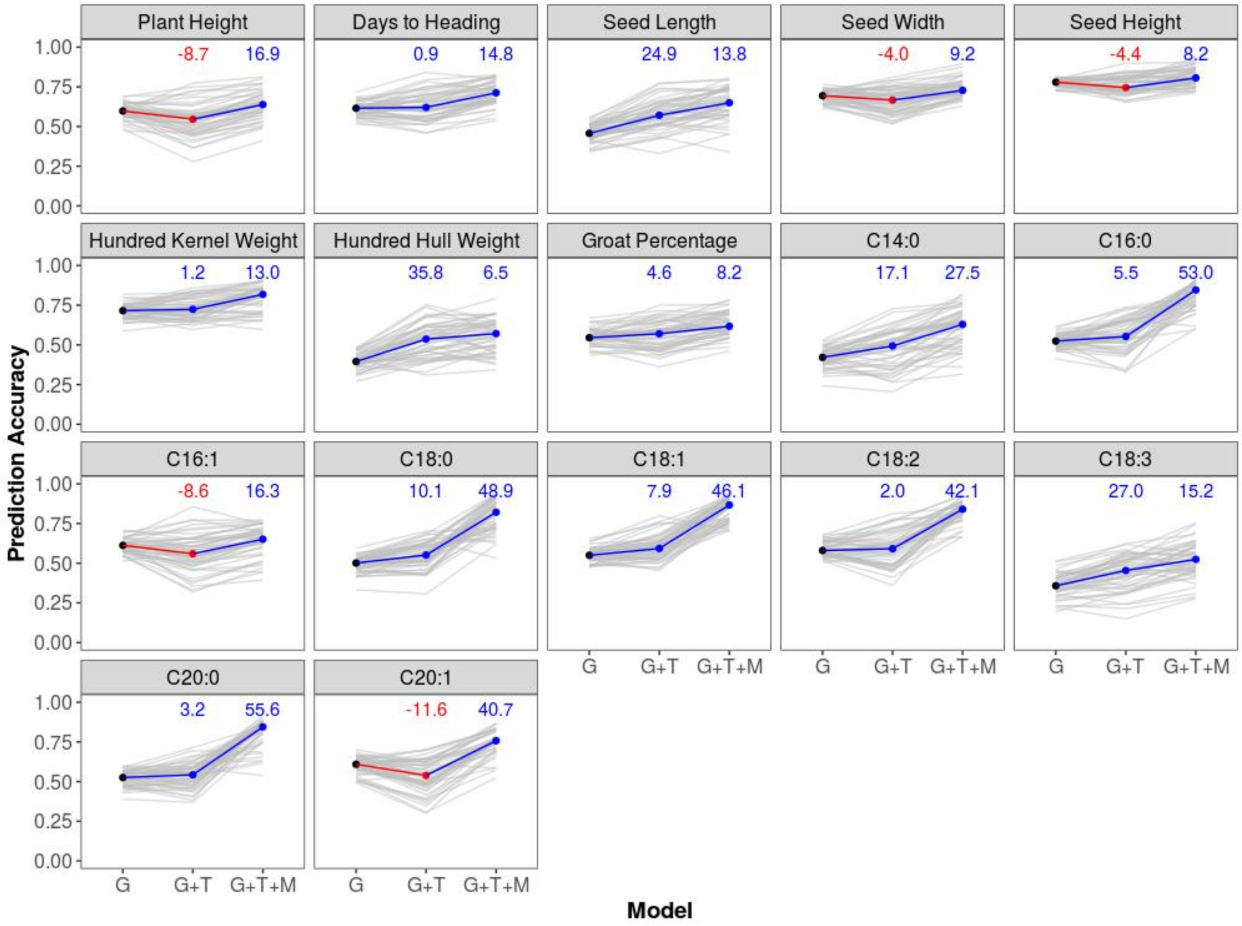

Fig. S3 Prediction accuracy changes from G to G+T models and from G+T to G+T+M models of the 17 phenotypic traits in the Diversity panel across 50 re-sampling runs. Each gray line represents a re-sampling run, and colored lines represent median prediction accuracy across the 50 re-sampling runs. Medians of percent change in prediction accuracy of models relative to a previous reduced model are indicated in blue if positive and in red if negative on top of each box. G = genomic BLUP, T = transcriptomic BLUP, M = metabolomic BLUP

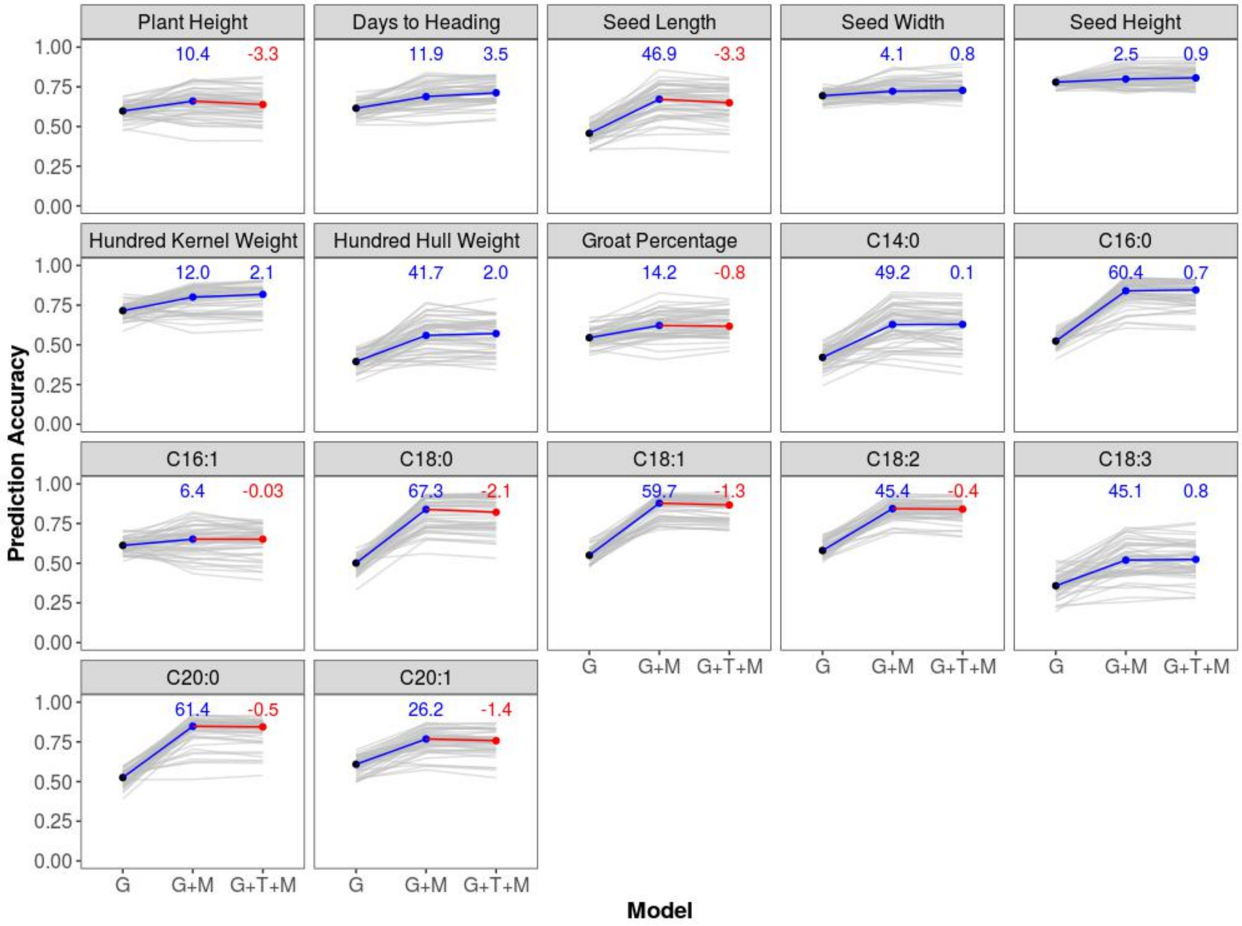

Fig. S4 Prediction accuracy changes from G to G+M models and from G+M to G+T+M models of the 17 phenotypic traits in the Diversity panel across 50 re-sampling runs. Each gray line represents a re-sampling run, and colored lines represent median prediction accuracy across the 50 re-sampling runs. Medians of percent change in prediction accuracy of models relative to a previous reduced model are indicated in blue if positive and in red if negative on top of each box. G = genomic BLUP, T = transcriptomic BLUP, M = metabolomic BLUP

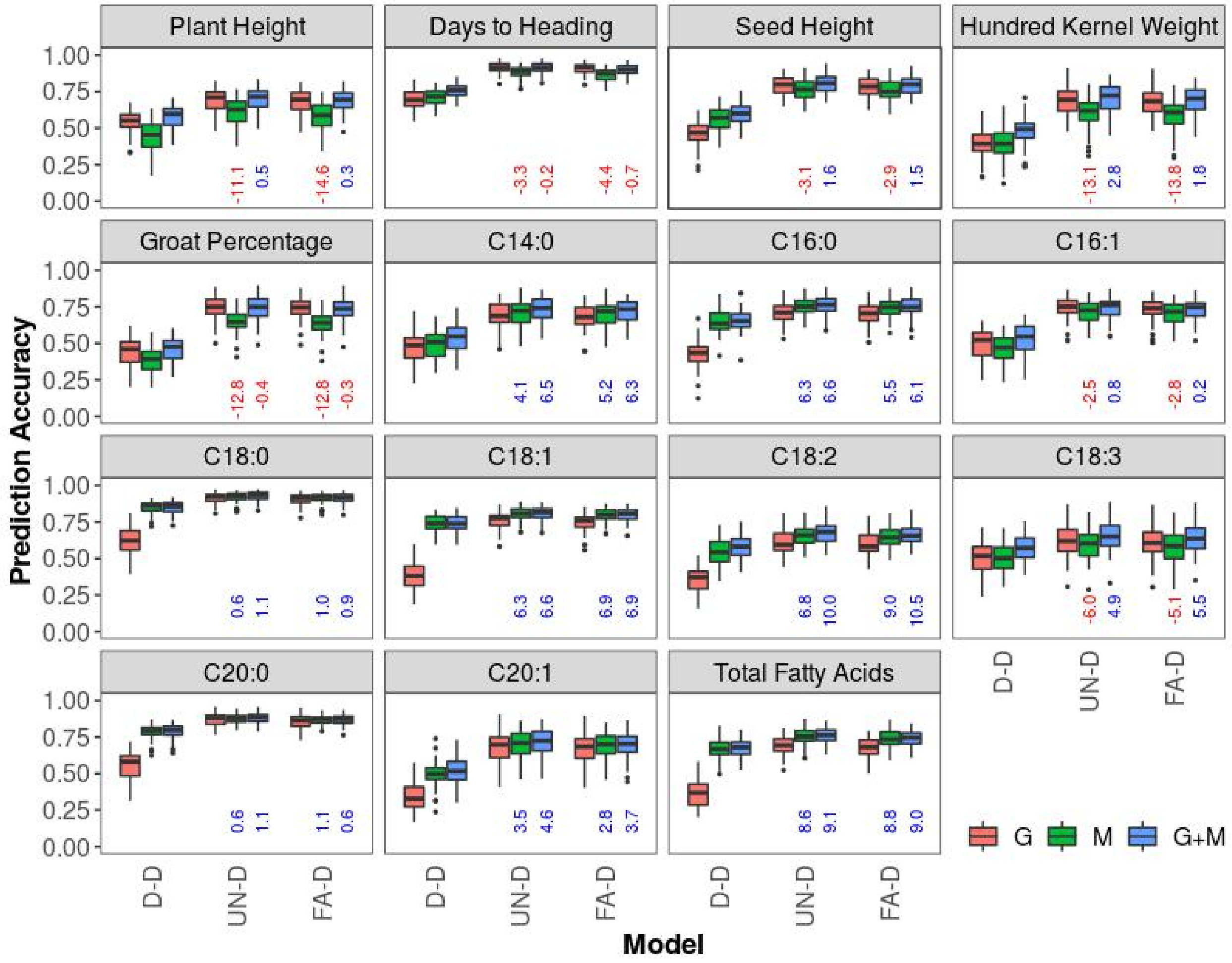

Fig. S5 Distribution of prediction accuracy of the 15 phenotypic traits in the Elite panel across 50 re-sampling runs estimated by multi-trait models of D-D, UN-D and FA-D. For each trait, boxplots with different colors represent models. Medians of percent change in prediction accuracy of M and G+M models relative to the G model are indicated below each box in blue if positive and in red if negative. For each model, the uppercase letters before and after the hyphen represent genetic and residual covariance structures: D=diagonal, UN=unstructured, FA=factor-analytic

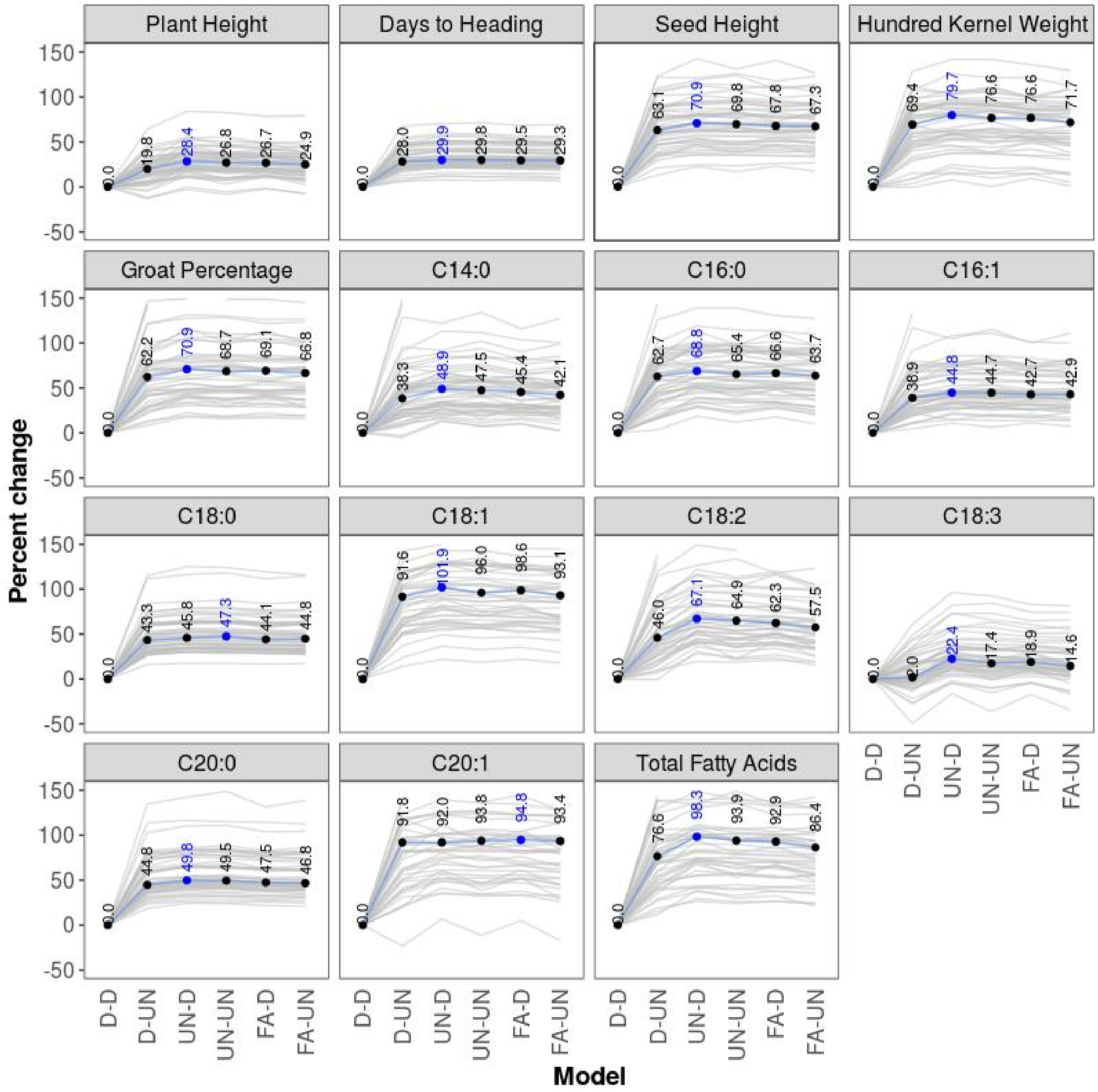

Fig. S6 Percentage changes in prediction accuracy estimated from multi-environment GBLUP models over single-environmental GBLUP for the 15 phenotypic traits in the Elite panel. Each gray line represents a re-sampling run, and the blue line represents median values across 50 re-sampling runs. The number in blue is the approach that showed the most improvement. For each model, the uppercase letters before and after the hyphen represent genetic and residual covariance structures: D=diagonal, UN=unstructured, FA=factor-analytic.

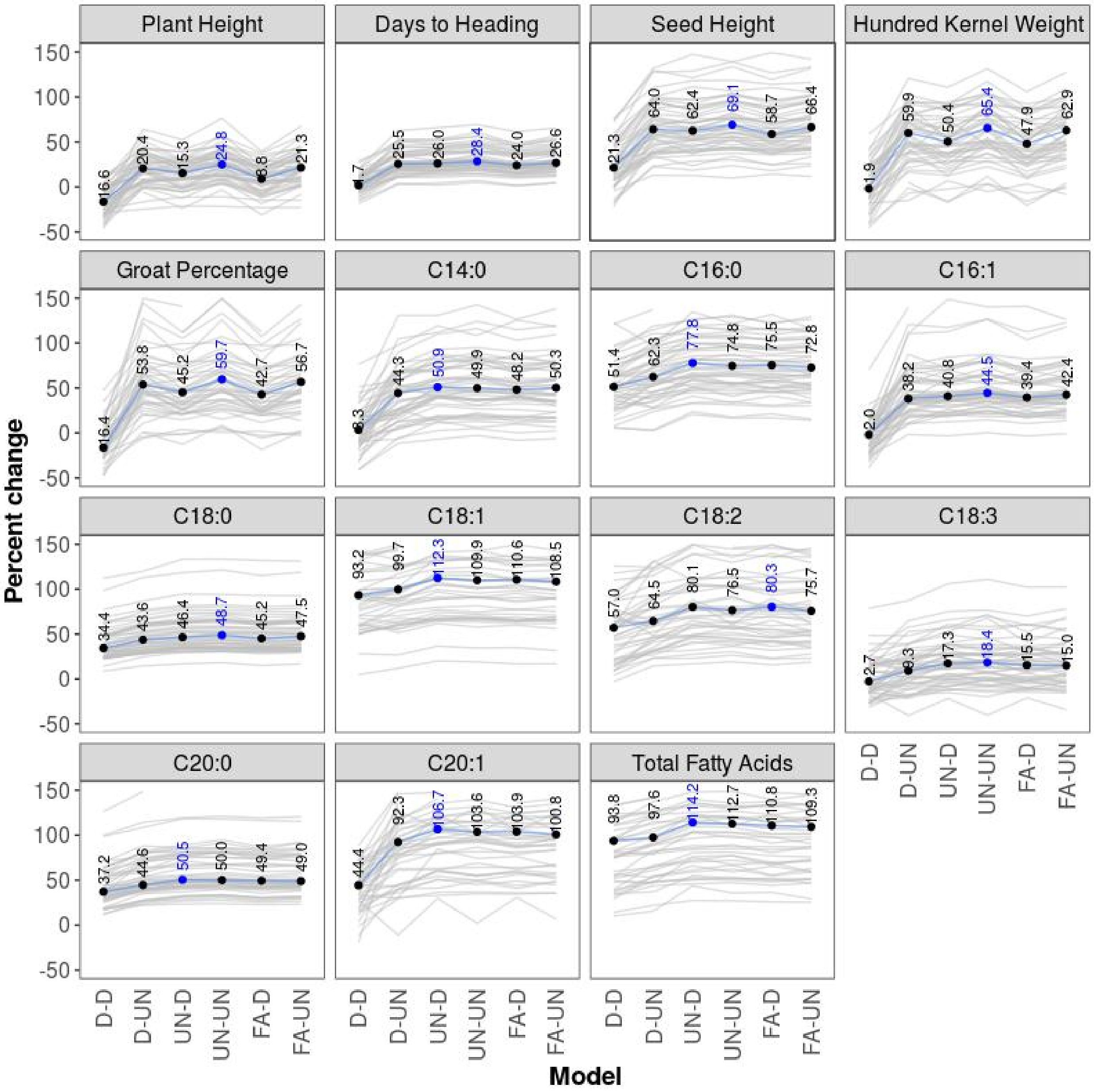

Fig. S7 Percentage changes in prediction accuracy estimated from multi-environment metabolite BLUP (M) models over single-environmental GBLUP for the 15 phenotypic traits in the Elite panel. Each gray line represents a re-sampling run, and the blue line represents median values across 50 re-sampling runs. The number in blue is the approach that showed the most improvement. For each model, the uppercase letters before and after the hyphen represent genetic and residual covariance structures: D=diagonal, UN=unstructured, FA=factor-analytic.

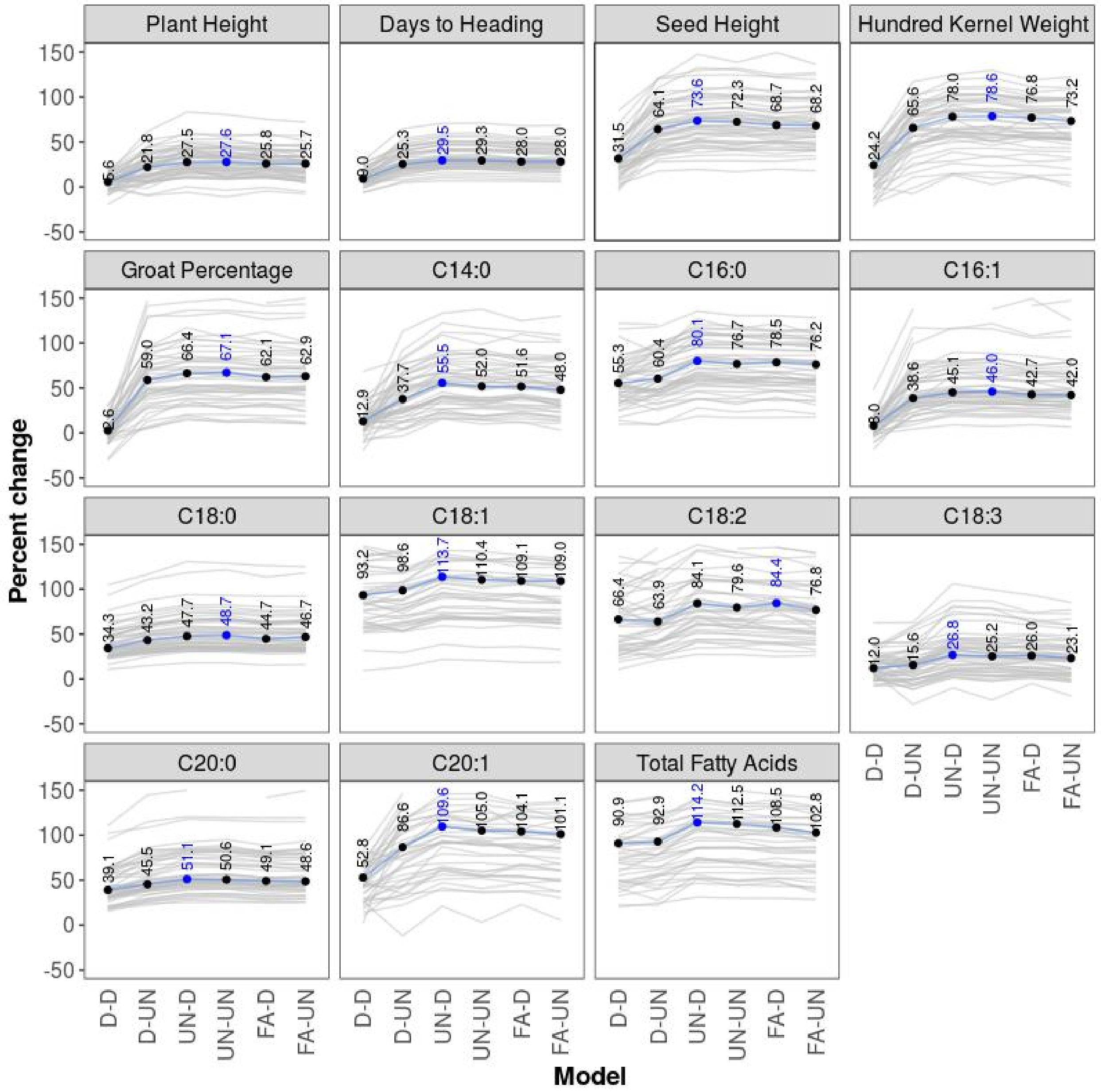

Fig. S8 Percentage changes in prediction accuracy estimated from multi-environment genomic and metabolomic BLUP (G+M) models over single-environmental GBLUP for the 15 phenotypic traits in the Elite panel. Each gray line represents a re-sampling run, and the blue line represents median values across 50 re-sampling runs. The number in blue is the approach that showed the most improvement. For each model, the uppercase letters before and after the hyphen represent genetic and residual covariance structures: D=diagonal, UN=unstructured, FA=factor-analytic.

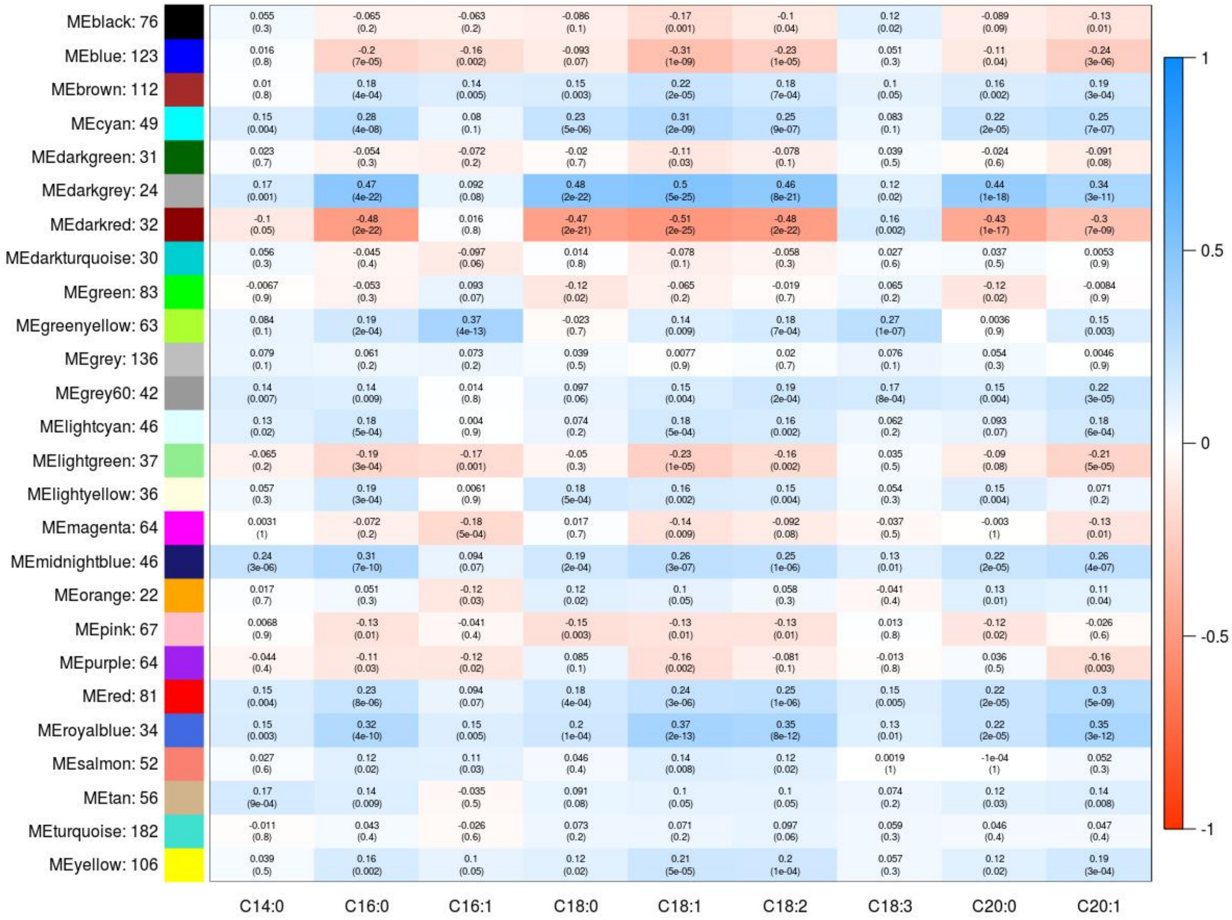

Fig. S9 Correlation between eigenvector of network modules and fatty acid traits. Each row corresponds to a module eigengene, column to a fatty acid trait. Each cell contains the corresponding correlation and p-value. The table is color-coded by correlation according to the color legend.

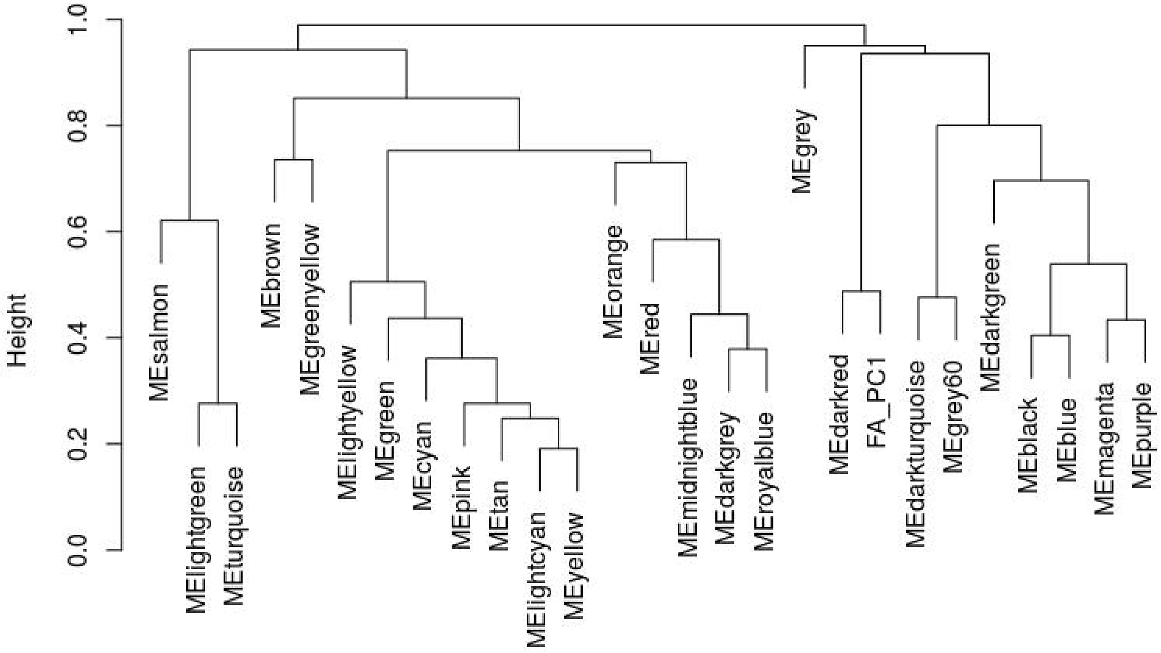

Fig. S10 Hierarchical clustering dendrogram of the network eigenvectors and PC1 of the nine fatty acids.

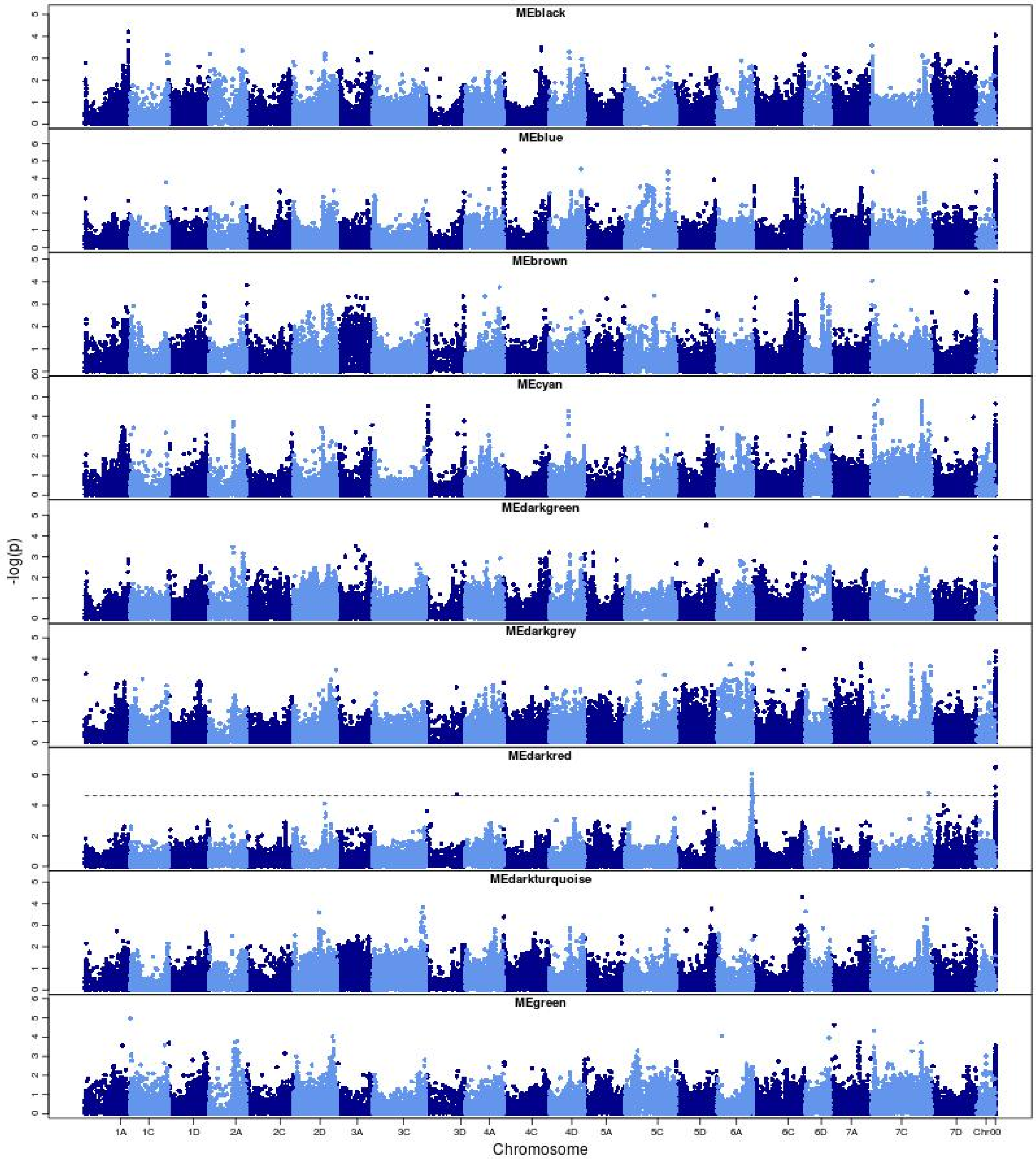

Fig. S11 Manhattan plots of eigenvectors of twenty-six network modules identified by WGCNA and PC1 of fatty acids in the Diversity panel. The dashed line corresponds to an FDR rate of 0.05 (continued on the next page).

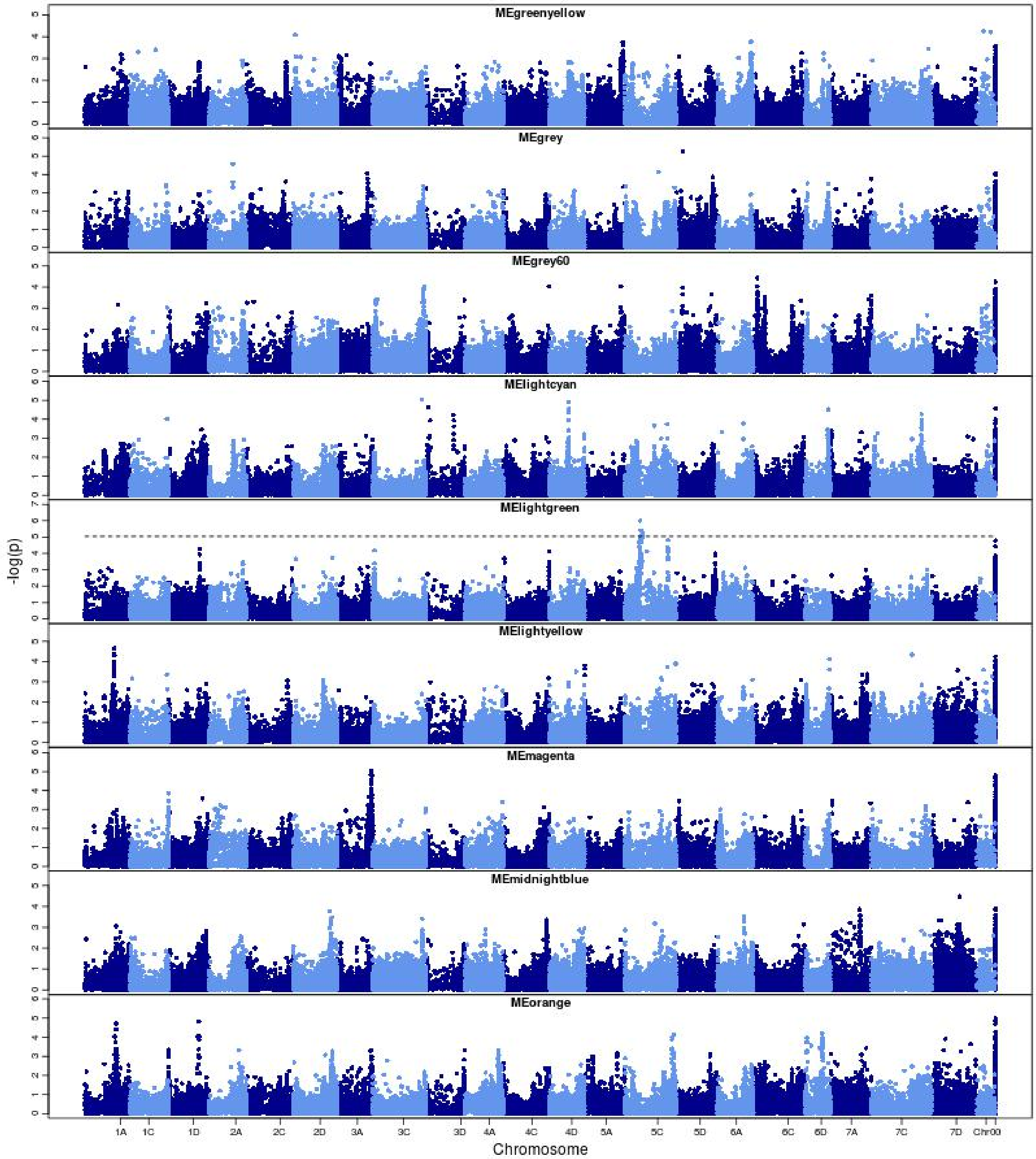

Fig. S11 Manhattan plots of eigenvectors of twenty-six network modules identified by WGCNA and PC1 of fatty acids in the Diversity panel. The dashed line corresponds to an FDR rate of 0.05 (continued on the next page).

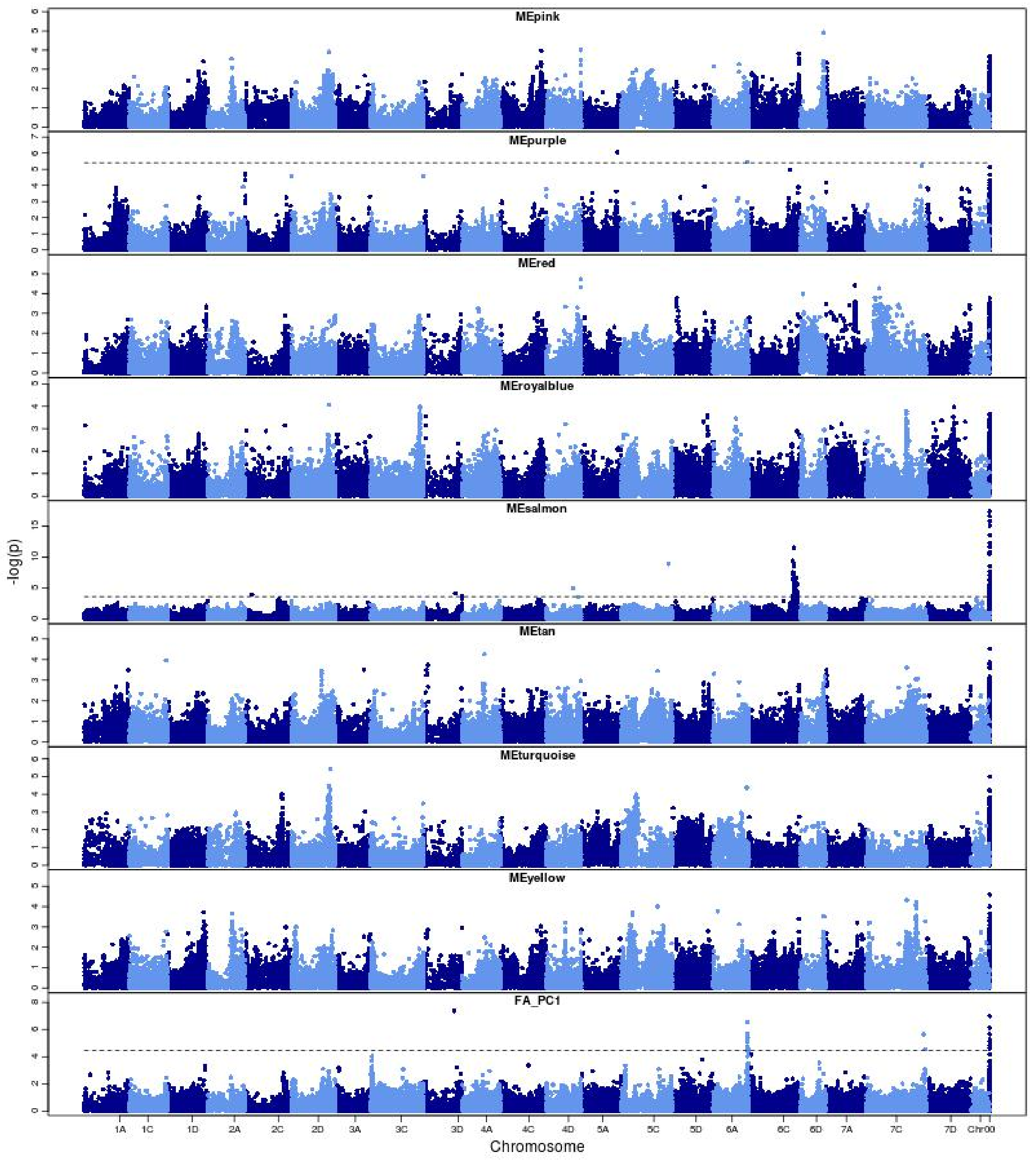

Fig. S11 Manhattan plots of eigenvectors of twenty-six network modules identified by WGCNA and PC1 of fatty acids in the Diversity panel. The dashed line corresponds to an FDR rate of 0.05.

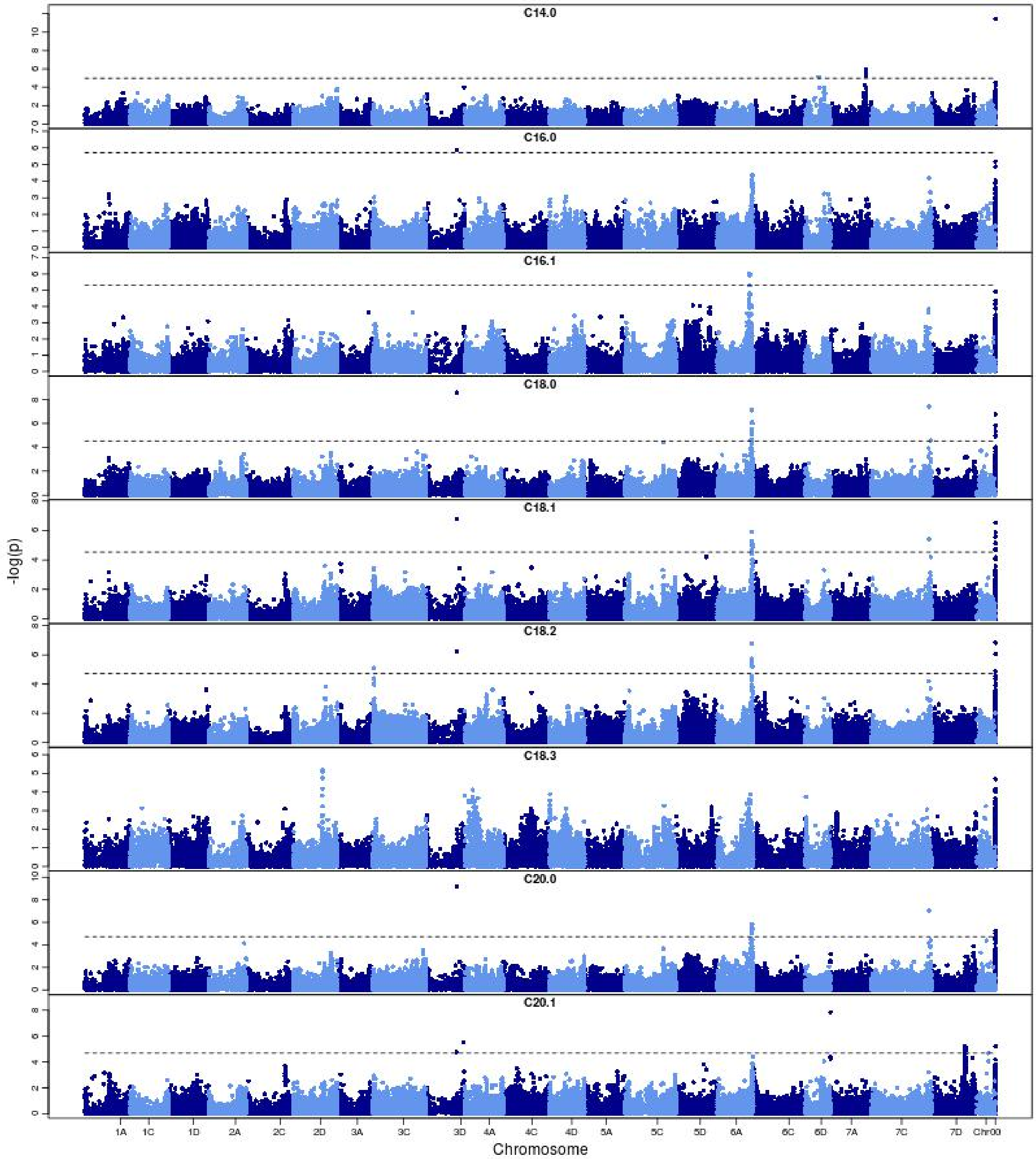

Fig. S12 Manhattan plots of fatty acids traits in the Diversity panel. The dashed line corresponds to an FDR rate of 0.05.

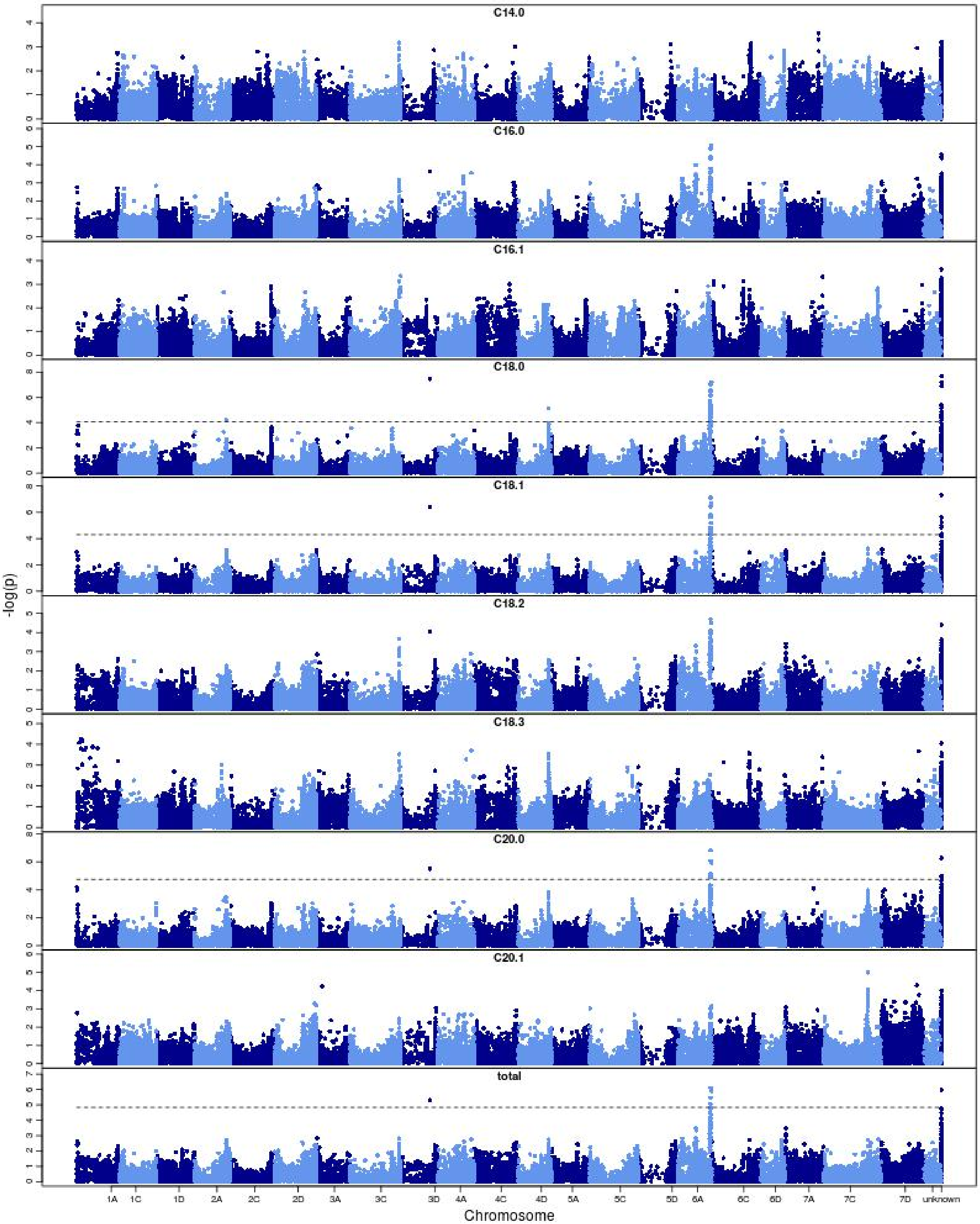

Fig. S13 Manhattan plots of fatty acids traits in the Elite panel. The fatty acids data was collected from filed trial located at Crookston, MN. The dashed line corresponds to an FDR rate of 0.05 (continued on the next page).

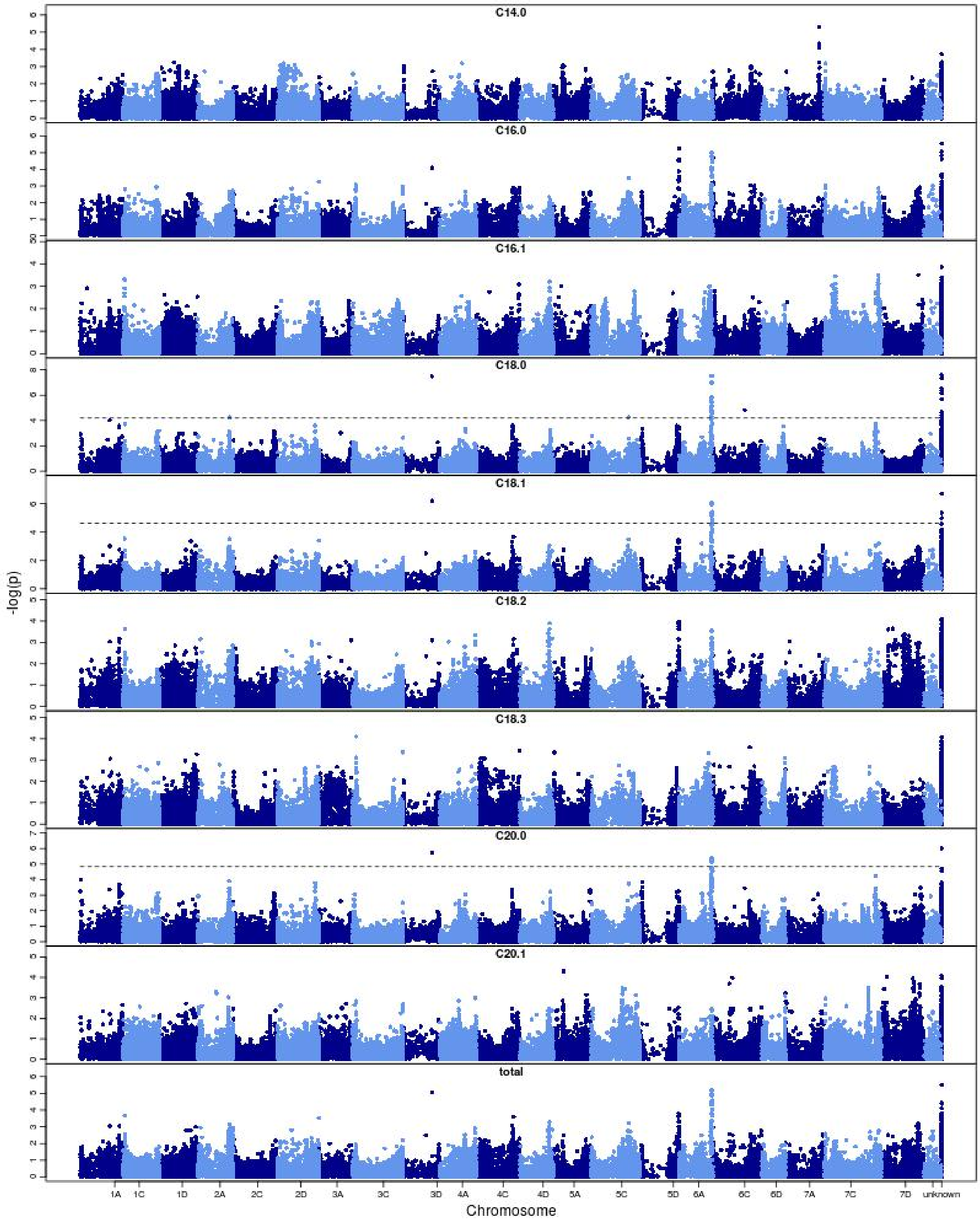

Fig. S13 Manhattan plots of fatty acids traits in the Elite panel. The fatty acids data was collected from filed trial located at Brookings, SD. The dashed line corresponds to an FDR rate of 0.05 (continued on the next page).

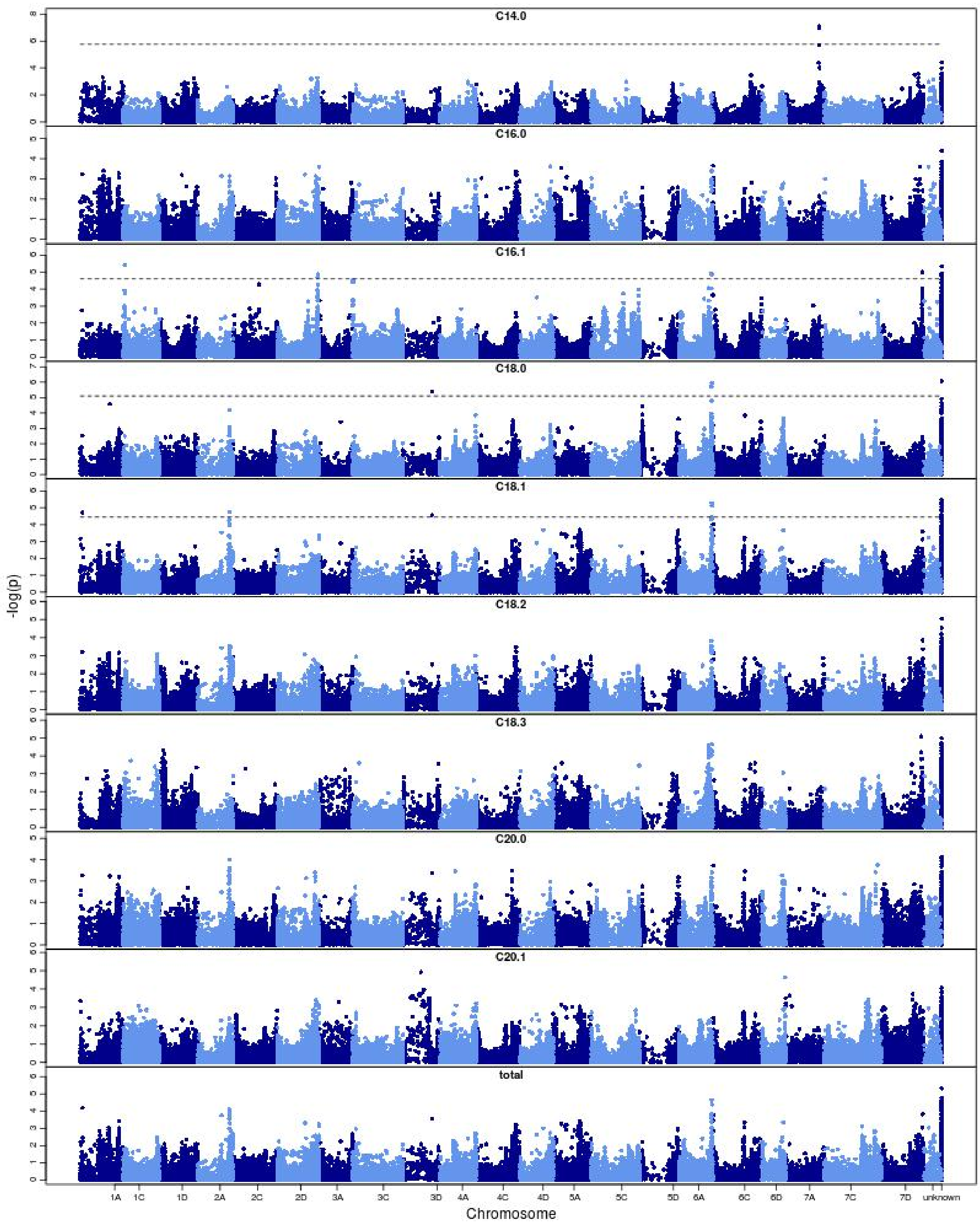

Fig. S13 Manhattan plots of fatty acids traits in the Elite panel. The fatty acids data was collected from filed trial located at Madison, WI. The dashed line corresponds to an FDR rate of 0.05.

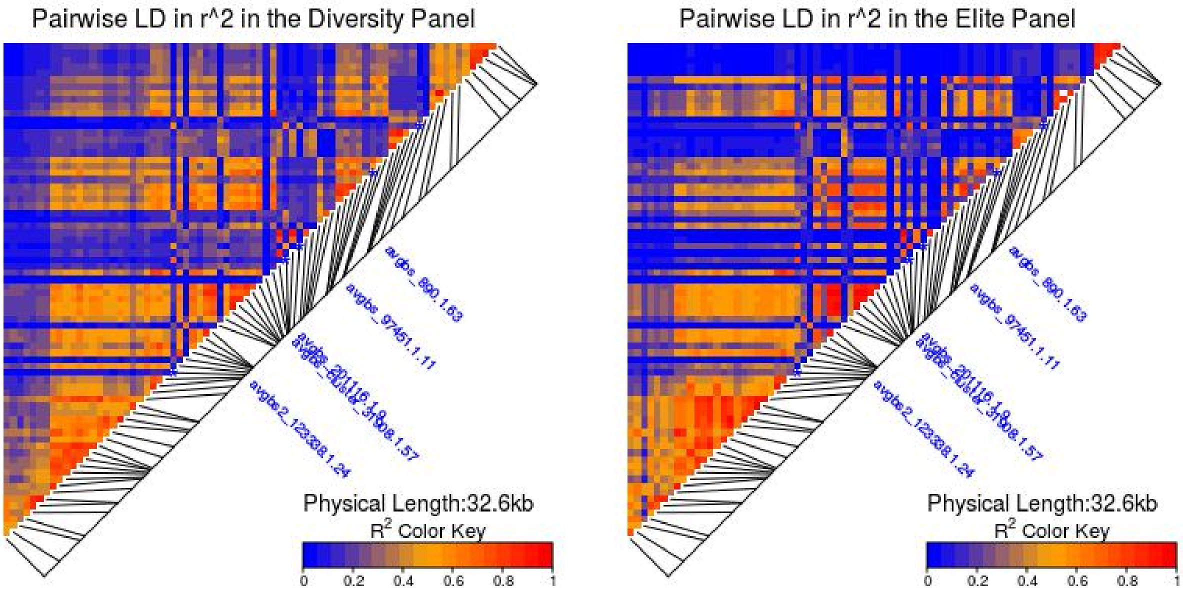

Fig. S14 LD heatmaps of significant markers of *QTL-6A* and the markers in LD with them in the Diversity and Elite panels. Only 75 SNPs on chromosome 6A with putative physical positions were used for the LD plot.The top 5 SNPs with highest marker score (-log_{10}Pvalue) were labeled.

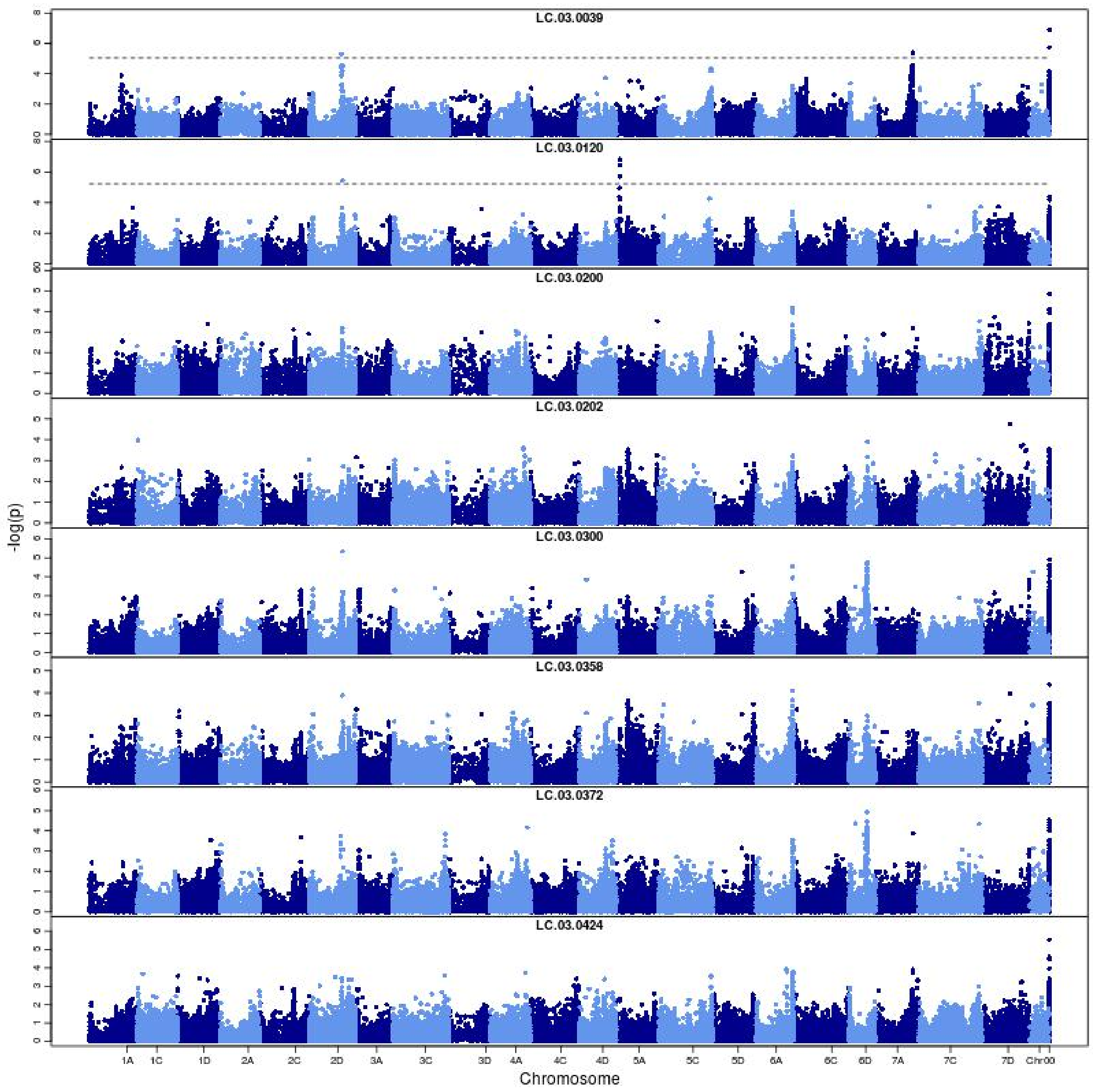

Fig. S15 Manhattan plots of metabolites in the darkred module identified by WGCNA in the Diversity panel. The dashed line corresponds to an FDR rate of 0.05 (continued on the next page).

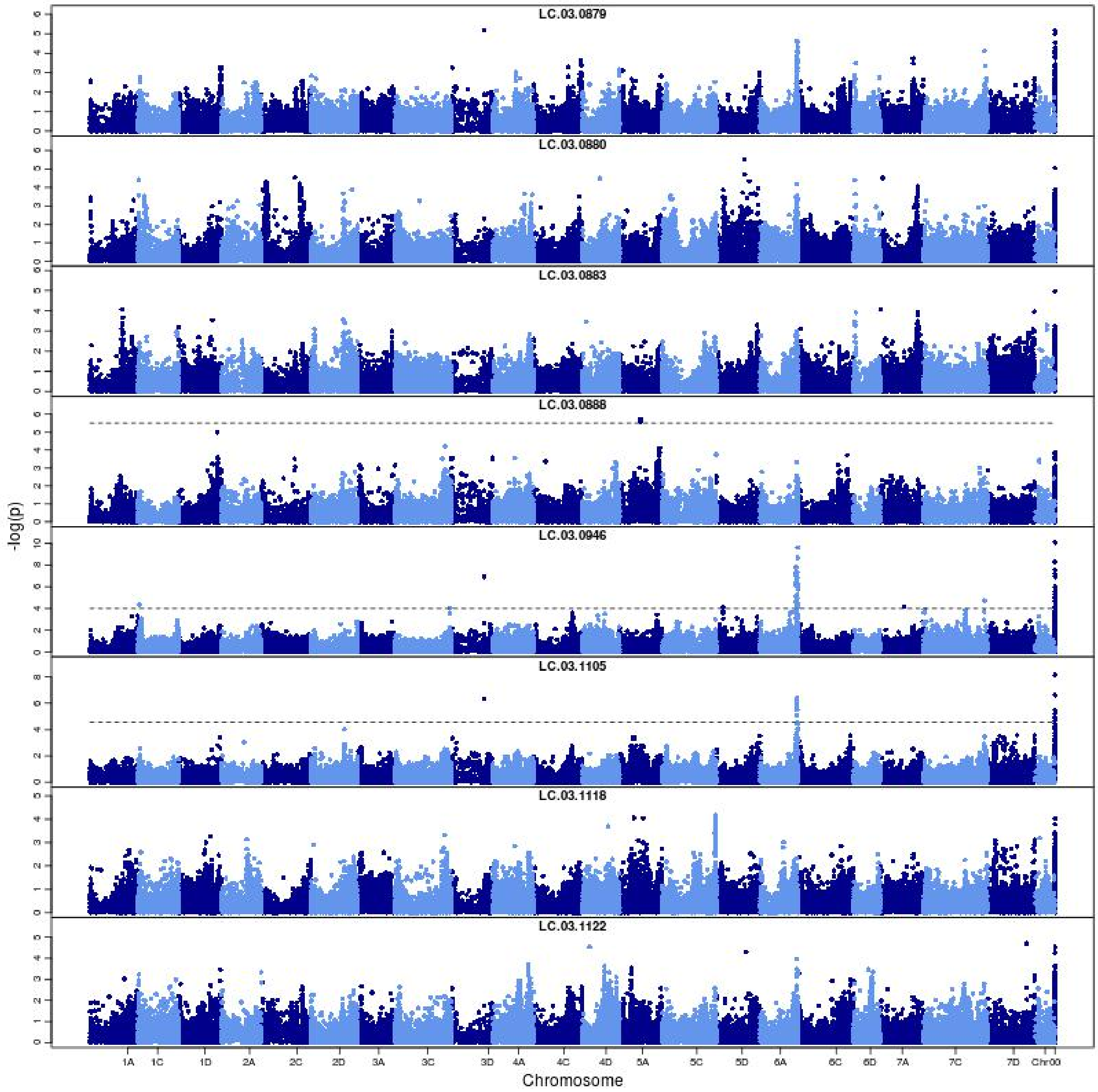

Fig. S15 Manhattan plots of metabolites in the darkred module identified by WGCNA in the Diversity panel. The dashed line corresponds to an FDR rate of 0.05 (continued on the next page).

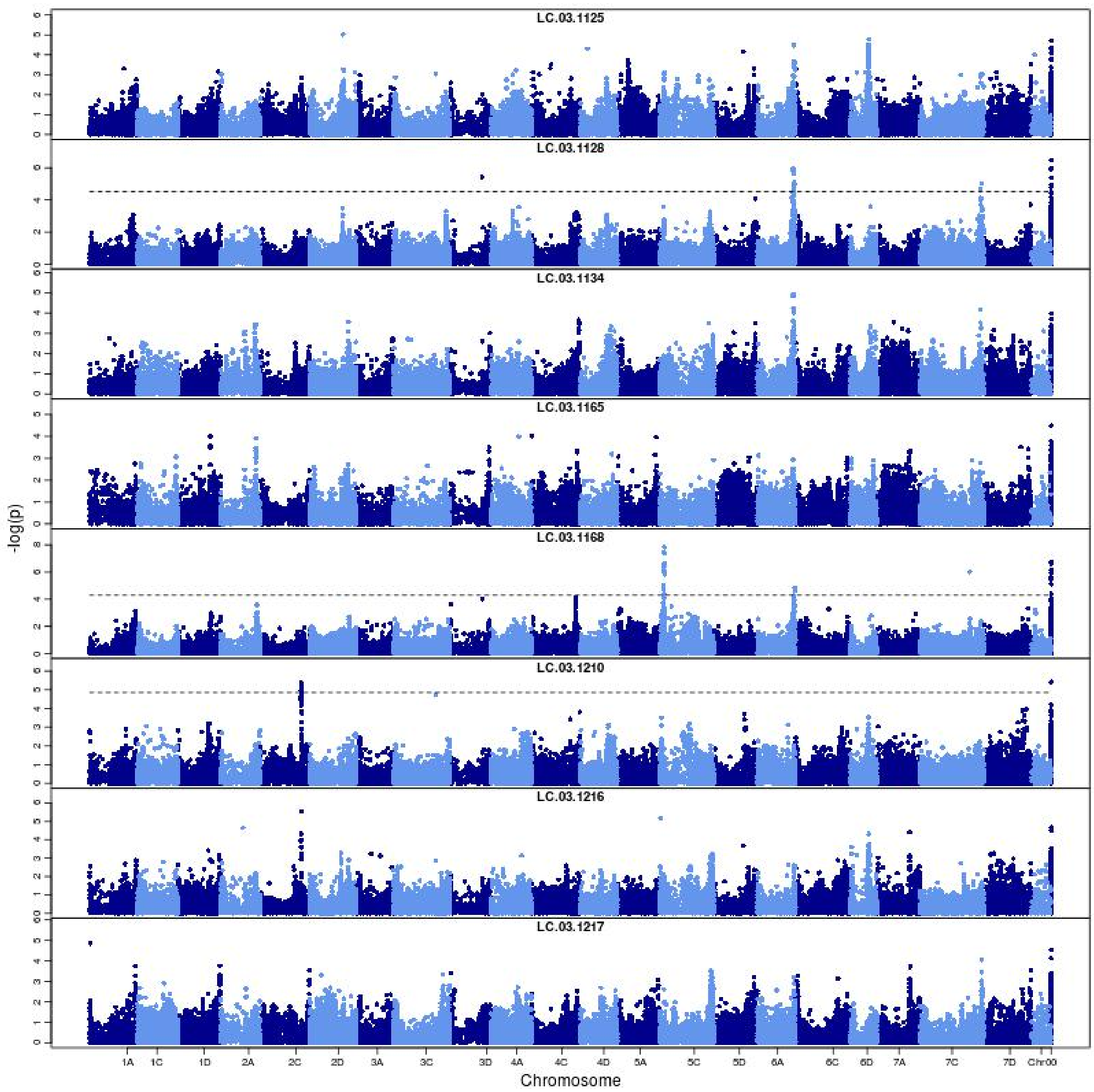

Fig. S15 Manhattan plots of metabolites in the darkred module identified by WGCNA in the Diversity panel. The dashed line corresponds to an FDR rate of 0.05 (continued on the next page).

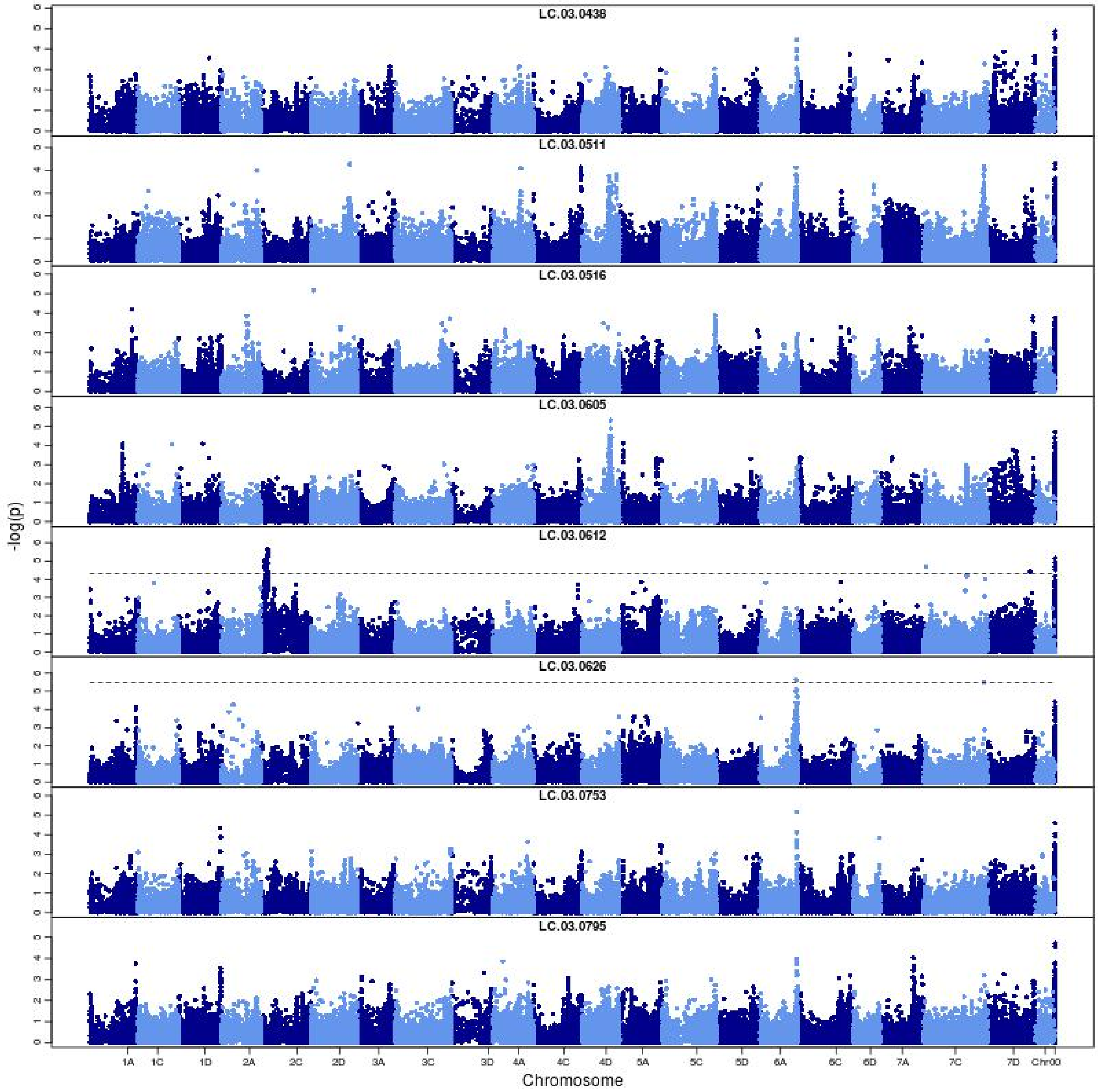

Fig. S15 Manhattan plots of metabolites in the darkred module identified by WGCNA in the Diversity panel. The dashed line corresponds to an FDR rate of 0.05.

Supplemental Tables

Table S1 Phenotypic traits evaluated in the Diversity panel and the Elite panel and their heritabilities

Table S2 A list of the 368 and 232 lines included in the Diversity panel and Elite panel

Table S3 Description of the multi-trait models

Table S4 Percent changes in prediction accuracy over GBLUP of 5, 10, 11, 17 and 17 traits from transcriptomic BLUP (T), metabolomic BLUP (M), G+T, G+M and G+T+M models in the Diversity panel.

Table S5 Annotation of Metabolites identified in the Diversity Panel

Table S6 Lipids and lipid-like molecules enrichment test in 26 network modules identified by WGCNA based on metabolite annotation

Table S7 Percent changes in prediction accuracy of G+M over GBLUP(G) and metabolomic BLUP (M) models for the 17 traits in the Diversity panel.

Table S1 Phenotypic traits evaluated in the Diversity panel and the Elite panel and their heritabilities

		Diversity Panel	Elite Panel			
Category	Trait Unknownme	ITH	Across-Site	MN	SD	WI
Agronomic	Plant Height	0.82	0.76	0.63	0.50	0.52
	Days to Heading	0.80	0.89	0.97	0.60	0.71
	Seed Length	0.79	0.81	0.81	–	0.62
	Seed Width	0.86	0.80	0.87	–	0.73
	Seed Height	0.89	0.78	0.76	0.82	0.69
	Hundred Kernel Weight	0.89	0.67	0.64	0.65	0.56
	Hundred Hull Weight	0.82	0.69	0.51	0.41	–
	Groat Percentage	0.88	0.77	0.84	0.90	0.46
Fatty Acids	C14:0	0.80	0.60	0.67	0.82	0.75
	C16:0	0.80	0.71	0.62	0.80	0.76
	C16:1	0.75	0.78	0.67	0.80	0.72
	C18:0	0.92	0.89	0.89	0.93	0.92
	C18:1	0.89	0.80	0.66	0.81	0.82
	C18:2	0.78	0.62	0.54	0.74	0.71
	C18:3	0.49	0.58	0.67	0.68	0.73
	C20:0	0.87	0.89	0.77	0.85	0.89
	C20:1	0.74	0.71	0.55	0.58	0.76
	Total Fatty Acids	–	0.70	0.58	0.78	0.76

ITH=Ithaca, MN=Minnesota, SD=South Dakota, WI=Wisconsin

Table S2 A list of the 368 and 232 lines included in the Diversity panel and Elite panel

Line Name	Population
IL09-5508	Elite
AAC_ROSKENS	Both
ND102000	Elite
WIX8787-3	Elite
IL06-3761	Elite
AAC_NICOLAS	Elite
IL11-5748	Elite
P973A38-9-3-2-29	Elite
OTEE	Elite
RON	Elite
MN04242	Elite
CLINTFORD	Both
SD120129	Elite
MN09115	Elite
SD050834	Elite
SD120640	Elite
BETAGENE	Both
SD110304	Elite
IA02130-2-2	Elite
SD110765	Elite
SD041445-119	Elite
P0541A1-1	Elite
SD080348	Elite
PI344841	Both
P0714A1-29-2	Elite

Line Name	Population
WIX9500-6	Elite
SD091510	Elite
SD120258	Elite
SD120524	Elite
MN08243	Both
SD120266	Elite
IL09-6937	Elite
SD110808	Elite
SD120456	Elite
IL04-7077	Both
CORRAL	Both
ND060182	Both
SD020883	Elite
WIX9878-3	Elite
MN06203	Elite
SD081644	Elite
MN08252	Both
BADGER	Elite
LEGGETT	Elite
P0528A1-6-1	Elite
MN10209	Elite
AAC_OAKLIN	Elite
SD110605	Elite
P021A1-66-2	Elite
P0528A1-1-1	Elite
WIX9528-1	Elite
ND030349	Elite

Line Name	Population
SD081107	Elite
SD100184	Elite
SD090880	Elite
KAME	Elite
FL0238BSB-22	Both
MN08160	Both
SD090893	Elite
MN07208	Elite
IL11-2353	Elite
IL08-2010	Elite
WPAT04P03-PY3B	Elite
IA111003	Both
AAC_ALMONTE	Elite
IL00-1030	Elite
OT2083	Elite
SD050938	Elite
P0216A1-1	Elite
ND050490	Elite
SD041405	Elite
P021A1-25	Elite
HORSEPOWER	Elite
IA02130-2-3	Elite
WIX8787-4	Elite
MNBT1021-1	Elite
AC_ASSINIBOIA	Elite
MN02234	Elite
SD050608	Elite

Line Name	Population
CLINTLAND64	Elite
OA1342-2	Elite
SD041016	Elite
OA1256-1	Elite
SD120553	Elite
SD090965	Elite
MN04136	Elite
TX02U7479	Both
IA02168-1-1	Elite
SD60130	Elite
IL02-8011	Both
LAO-1012-040	Elite
MN10130	Elite
ND090868	Elite
WIX9287-2	Elite
MN04120	Elite
COLT	Elite
ND051306	Elite
IL08-9201	Elite
QUALITY|PI289587	Both
MN06120	Elite
SABER	Elite
WIX10088-6	Elite
MN03114	Elite
ND021612	Elite
MN10253	Elite
X9221-8	Both

Line Name	Population
OA1341-1	Elite
HA07-02X22-3	Elite
P075A1-7	Elite
OA1357-2	Elite
MN08138	Elite
SD111922	Elite
SD041451	Elite
SD100719	Elite
EXCEL	Both
IL05-7133	Elite
SD050945	Elite
SD090510	Elite
SD090552	Elite
ND111357	Elite
WIX9449-1	Elite
P9741A1-4-6-86	Elite
IL05-9931	Elite
SD030888	Elite
ND060342	Both
MN09255	Elite
ND070388	Elite
SD090780	Elite
SD081949	Elite
MN05237	Elite
SD081577	Elite
IA02010-2-1	Elite
WIX10045-9	Elite

Line Name	Population
SD100198	Elite
SD082192	Elite
MN09230	Elite
SD081085	Elite
ND040492	Elite
MN11221	Elite
OT2074	Elite
SD100602	Elite
MN11110	Elite
OA1331-6	Elite
WIX9487-1	Elite
ND040250	Elite
SD031128	Elite
OA1271-3	Elite
UnknownTTY	Elite
WIX10045-12	Elite
MN05119	Elite
LAO-1104-028C1	Elite
SD080788	Elite
95AB12770	Both
SD61081	Elite
ND040196	Elite
MN09223	Elite
SD050716	Elite
HA08-03X15-1	Elite
IL05-3337	Elite
SD081563	Elite

Line Name	Population
MN06213	Elite
P075A1-3-2	Elite
WIX9645-1	Elite
MN08260	Both
WIX9414-1	Elite
P021A1-78-1	Elite
04P07A-BS5C	Elite
SD110640	Elite
ND090807	Elite
GOPHER	Elite
SD100015	Elite
MN05157	Elite
SUMO	Elite
SD120316	Elite
WIX9562-5	Elite
MN05205	Elite
WIX9897-5	Elite
06P04A-E07B3	Elite
P0216A1-1-45	Elite
MN09105	Elite
SD100454	Elite
ND021052	Elite
SD110470	Elite
IL06-8153	Elite
MN10121	Elite
TX07CS1402	Both
SD050616	Elite

Line Name	Population
IA01005-2	Elite
ND100362	Elite
SD081038	Elite
MN09103	Elite
DON	Elite
MN09256	Elite
IL09-5239	Elite
MN07204	Elite
IL09-5745	Elite
OMSKIJ19260	Elite
SHELBY427	Elite
SD61433	Elite
SD051005	Elite
SD101027	Elite
SD080611	Elite
IL06-5465	Elite
OPTIMUM	Both
SD100644	Elite
WIX9150-1	Elite
ND101473	Elite
HAYDEN	Elite
IL02-8663	Elite
00P28-AN01B1	Elite
CIAV5218	Both
SD090240	Elite
MORTON	Elite
MN02231	Elite

Line Name	Population
WIX8791-1	Elite
04P07B-GN1C	Elite
ND051312	Both
WIX9082-1	Both
SD60980	Elite
MN08211	Elite
SD111736	Elite
MN04232	Elite
ND070182	Elite
MN08130	Both
SHERWOOD	Both
IL05-8515	Both
PERDEBERG	Both
ANDREW	Both
X8826-1	Both
WOODBURN	Both
DEON	Elite
OGLE	Elite
9876C1-2-1-5-2-4-1	Diversity
0219A1-84-4-4-4-4	Diversity
IL05-10069	Diversity
WHITE_TARTARIAN|CIAV800	Diversity
IA111188	Diversity
X9410-2	Diversity
GERE	Diversity
02HO-139	Diversity
OA1232-2	Diversity

Line Name	Population
MN861900	Diversity
LANG-DOERFLERS_WEIHENSTEPHANER_WEISSHAFER|PI180932	Diversity
RODGERS	Diversity
RANCH	Diversity
ND060223	Diversity
X9287-2	Diversity
CIAV5389	Diversity
X9507-1	Diversity
IL05-1705	Diversity
IA111041	Diversity
027A1-87-8-1	Diversity
OA1250-2	Diversity
PI577862	Diversity
OA1226-4	Diversity
LAO-1135-015	Diversity
IA111006	Diversity
IA111186	Diversity
IA111153	Diversity
DRUMMOND	Diversity
SA070906	Diversity
04P07B-GY5E	Diversity
OA1248-1	Diversity
NIAGARA	Diversity
IA111235	Diversity
OA1197-1	Diversity
PI159180	Diversity
CEUnknownD88|PI361889	Diversity

Line Name	Population
IA111281	Diversity
ABERDEEN_SELECTION1939-2005	Diversity
TIOGA	Diversity
IA111199	Diversity
ENDRESS	Diversity
FLORIDA500	Diversity
ONOHOJSKIJ_A-547	Diversity
SA070781	Diversity
X8903-2	Diversity
IA111036	Diversity
RANSOM	Diversity
IA111180	Diversity
MN08268	Diversity
CIAV6209	Diversity
CIAV5220	Diversity
ND060111	Diversity
IA111136	Diversity
IA111045	Diversity
CDC_BIG_BROWN	Diversity
SA071616	Diversity
IA111027	Diversity
ND061813	Diversity
MN06125	Diversity
00P06-HD1D	Diversity
IA111119	Diversity
ND061614	Diversity
PI168094	Diversity

Line Name	Population
059A1-2-2-4	Diversity
HA05AB20-1	Diversity
FLAMINGSKOMET	Diversity
02G31-NL7A	Diversity
CD3774	Diversity
II-30-39	Diversity
SA061148	Diversity
AVOINE_NUE-NUE_NOISE|PI401772	Diversity
CIAV5222	Diversity
SA071760	Diversity
IA111071	Diversity
LAO-1136-014	Diversity
HAZEL	Diversity
KHARKOVSKIJ596|PI158223	Diversity
BANKUT_GALBEN	Diversity
TX07CS1039	Diversity
PI577985	Diversity
OA1263-2	Diversity
KOLBU	Diversity
TX07CS2140	Diversity
MN861218	Diversity
PENNCOMP38	Diversity
HORIZON270	Diversity
SYLVA	Diversity
GRANE	Diversity
OA1234-1	Diversity
FLORILAND	Diversity

Line Name	Population
CIAV5666	Diversity
TX05CS542	Diversity
MN08234	Diversity
IA111228	Diversity
HOHENHEIMER_V|PI180928	Diversity
97AB7767	Diversity
IA111158	Diversity
ND051236	Diversity
IA111178	Diversity
IA111123	Diversity
SA070655	Diversity
KRYMSKIJ90|PI296174	Diversity
26-35-B69	Diversity
Y-90	Diversity
JERRY	Diversity
CDC_WEAVER	Diversity
HORIZON201	Diversity
IL05-9948	Diversity
TYLER	Diversity
ORBIT	Diversity
IA111018	Diversity
CIAV5928	Diversity
SELECTION3841-1	Diversity
GOLDEN_RUSTPROOF|CIAV1751	Diversity
CDC_SOL-FI	Diversity
X345-1-B4-20-1	Diversity
SEVERIANIN|PI326231	Diversity

Line Name	Population
IA111218	Diversity
CILLA	Diversity
IA111044	Diversity
OA1266-1	Diversity
TIFT	Diversity
FL0047-J9	Diversity
X9396-1	Diversity
BIRI	Diversity
CHLUMECKY	Diversity
IA111282	Diversity
STANTON|PI412928	Diversity
00P01-A11A4	Diversity
CDC_MINSTREL	Diversity
CIAV6130	Diversity
IA111046	Diversity
X9195-2	Diversity
IA00020-12-3	Diversity
TENNESSEE_SELECTION090	Diversity
IA111185	Diversity
IL03-7936	Diversity
BRANCH	Diversity
LA02012-S-B-139-S2-B-S2-B-S2	Diversity
PENNLINE6571	Diversity
MN08270	Diversity
IL06-3751	Diversity
LA604	Diversity
IA111161	Diversity

Line Name	Population
IL06-3258	Diversity
SA070469	Diversity
151C-2	Diversity
OT380	Diversity
IA111004	Diversity
MN08230	Diversity
WIR4301	Diversity
LUTZ	Diversity
HUSAR	Diversity
MN08238	Diversity
DAWSON	Diversity
PA7967-11759	Diversity
X9421-3	Diversity
IA111200	Diversity
BIHARIA	Diversity
IA111177	Diversity
IA111232	Diversity
IL04-2727	Diversity
MN08132	Diversity
IL05-9330	Diversity
IL86-4189	Diversity
IL05-3806	Diversity
WIR4672	Diversity
MARION_QC	Diversity
IL75-5743	Diversity
SA060605	Diversity
MAIDA	Diversity

Line Name	Population
LAO-882-036	Diversity
SA070592	Diversity
CIAV6218	Diversity
SA071405	Diversity
IA111208	Diversity
IA111244	Diversity
Y-498	Diversity
MOHAWK	Diversity
04P06A-CY5D	Diversity
KAPP	Diversity
SA060830	Diversity
X9290-2	Diversity
BLANCHE_DE_HONGRIE	Diversity
IA111072	Diversity
LENROC	Diversity
VI56	Diversity
SA070972	Diversity
PA7733-1268	Diversity
IA111149	Diversity
X397-1-B5-2|PI605547	Diversity
GN04399	Diversity
IA111221	Diversity
LANG	Diversity
HA05AB38-22	Diversity
ND080724	Diversity
WIR4071	Diversity
OSAGE	Diversity

Line Name	Population
X9384-2	Diversity
IA111165	Diversity
57A-3	Diversity
OA1180-5	Diversity
MN08251	Diversity
IA111215	Diversity
IA111222	Diversity
LONDRIUnknown	Diversity
MN08129	Diversity
IA111002	Diversity
HA05AB29-17	Diversity
TIPPECANOE	Diversity
FL03001BSB-S7	Diversity
LISCHOWER_FRUHHAFER|CIAV3799	Diversity
SA060716	Diversity
OT399	Diversity
REEVES	Diversity
IL00-7070	Diversity
IA111162	Diversity
CORNELLIAN	Diversity
SKOROSPELKS	Diversity
IL75-5665	Diversity
MN08254	Diversity
PI159172	Diversity
CIAV5925	Diversity
IA111070	Diversity
BENDERY878A|PI258703	Diversity

Line Name	Population
CIAV5033	Diversity
IA111137	Diversity
OA1268-3	Diversity
FLAEMINGSNOVA	Diversity
FL03129-AB7	Diversity
CDC_ORRIN	Diversity
IA111109	Diversity
DUPPAWSKI	Diversity
IL98-10145	Diversity
SELECTION3863-8	Diversity
TX02U7605	Diversity
AMES_SELECTION4103	Diversity
M1-7	Diversity
FREDDY	Diversity
ND050506	Diversity
CLINTLAND60	Diversity
IA111077	Diversity
MN08225	Diversity
ND071521	Diversity
IA111012	Diversity
053B1-95	Diversity
74C-1	Diversity
ROBUST	Diversity
MN08139	Diversity
IA111238	Diversity
IA111155	Diversity
CIAV6227	Diversity

Line Name	Population
BELINDA	Diversity
ABERDEEN_SELECTION1939-3927	Diversity
IL05-3928	Diversity
NEWDAK	Diversity
2A-3	Diversity
ND060464	Diversity
X9258-5	Diversity
77NZ_AA322	Diversity
IA111121	Diversity
ND051467	Diversity
X9285-1	Diversity
MN08253	Diversity
IA111144	Diversity
IL05-6223	Diversity
SA070452	Diversity
ND051069	Diversity
X9270-4	Diversity
TERRY	Diversity
OT3028	Diversity
NES	Diversity
ABEGWEIT	Diversity
ND051037	Diversity
VALLEY	Diversity
HY174-OA	Diversity
CDC_SEABISCUIT	Diversity
IL03-2658	Diversity
CIAV5019	Diversity

Line Name	Population
LAO-1134-022	Diversity
IA111171	Diversity
SA070860	Diversity
IA111138	Diversity
ROUnknownLD	Diversity
HA05AB41-38	Diversity
CHERNIGOVSKIJ27B	Diversity
VI2	Diversity
P1-9	Diversity
IL2294-8	Diversity
ND050578	Diversity
X9396-4	Diversity
TX02U7047	Diversity
SD751187	Diversity
ODAL	Diversity
DELAIR	Diversity
IA10033	Diversity
G2-8	Diversity
IA111066	Diversity
PI193957	Diversity
RED_TEXAS|CIAV1914	Diversity
MISSOURI04103	Diversity
02G31-NU6D	Diversity
CD3708	Diversity
BARAGAN114	Diversity
ND060652	Diversity
KINVARRA_NO_A-8	Diversity

Line Name	Population
X9410-1	Diversity
OA1130-1	Diversity
MN06108	Diversity
IA111279	Diversity
IA111236	Diversity
ALLEN	Diversity
02G31-NN4B	Diversity
PI436071	Diversity
IA111192	Diversity
FULGRAIN_SELECTION|CIAV4565	Diversity
001A1-24-2-4-1-3	Diversity
FL03167BSB-147	Diversity
COLBERSON	Diversity
LA03046SBS7-B-S1	Diversity
IA111146	Diversity
ARIANE|PI361884	Diversity
LEUnknown	Diversity
SA070845	Diversity
ND072258	Diversity
SEVERNYJ209	Diversity
TX07CS1268	Diversity
SA01223-02	Diversity
IA111069	Diversity
CAUCAZ4275	Diversity
TX07CS2201	Diversity
TX02U7097	Diversity
X8995-4	Diversity

Line Name	Population
CIAV4143	Diversity
SECRETARIAT_LA495	Diversity
CD3737	Diversity

Table S3 Description of the multi-trait models

		Covariance structure[a]	
ID	Code	G	R
1	D-D	D	D
2	D-UN	D	UN
3	UN-D	UN	UN
4	UN-UN	UN	UN
5	FA-D	FA	D
6	FA-UN	FA	UN

[a]when fitted a multi-trait models with two kernels (G+M), G kernel and M kernel had the same genetic and residual covariance structures.

D=diagonal, UN=unstructured, FA=factor-analytic.

G=genetic covariance structure, R= residual covariance structure

Table S4 Percent changes in prediction accuracy over GBLUP of 5, 10, 11, 17 and 17 traits from transcriptomic BLUP (T), metabolomic BLUP (M), G+T, G+M and G+T+M models in the Diversity panel.

Model	TraitName	Percent Change
T	Days to Heading	12.4
T	Seed Length	17.9
T	Hundred Hull Weight	21.6
T	C16:1	0.7
T	C18:3	21.4
M	Days to Heading	6.0
M	Hundred Hull Weight	8.0
M	Groat Percentage	1.9
M	C16:0	51.5
M	C18:0	58.1
M	C18:1	52.0
M	C18:2	34.9
M	C18:3	23.5
M	C20:0	56.3
M	C20:1	18.4
G+T	Days to Heading	0.1
G+T	Seed Length	24.1
G+T	Hundred Kernel Weight	0.9
G+T	Hundred Hull Weight	32.5
G+T	Groat Percentage	3.0
G+T	C14:0	17.7
G+T	C16:0	8.4
G+T	C18:0	9.7
G+T	C18:1	8.2

Model	TraitName	Percent Change
G+T	C18:3	25.4
G+T	C20:0	0.7
G+M	Plant Height	11.3
G+M	Days to Heading	13.6
G+M	Seed Length	42.9
G+M	Seed Width	5.0
G+M	Seed Height	4.4
G+M	Hundred Kernel Weight	13.4
G+M	Hundred Hull Weight	39.7
G+M	Groat Percentage	14.3
G+M	C14:0	54.3
G+M	C16:0	63.4
G+M	C16:1	3.8
G+M	C18:0	70.3
G+M	C18:1	58.1
G+M	C18:2	43.4
G+M	C18:3	48.8
G+M	C20:0	64.5
G+M	C20:1	28.1
G+T+M	Plant Height	8.0
G+T+M	Days to Heading	17.3
G+T+M	Seed Length	43.2
G+T+M	Seed Width	6.6
G+T+M	Seed Height	5.8
G+T+M	Hundred Kernel Weight	14.5
G+T+M	Hundred Hull Weight	42.1
G+T+M	Groat Percentage	15.5

Model	TraitName	Percent Change
G+T+M	C14:0	52.2
G+T+M	C16:0	58.1
G+T+M	C16:1	6.0
G+T+M	C18:0	68.0
G+T+M	C18:1	56.0
G+T+M	C18:2	42.4
G+T+M	C18:3	48.4
G+T+M	C20:0	62.0
G+T+M	C20:1	26.3

Table S5 Annotation of Metabolites identified in the Diversity Panel

Compound	Retention Time	Super class
GC.03.0001	905.342419	Organic oxygen compounds
GC.03.0002	873.6860748	Organic oxygen compounds
GC.03.0003	913.280496	Organic oxygen compounds
GC.03.0004	1079.375838	Organic oxygen compounds
GC.03.0005	608.4465354	Organic oxygen compounds
GC.03.0006	615.0757784	Organic oxygen compounds
GC.03.0007	622.9920508	Organic oxygen compounds
GC.03.0008	562.0521686	NA
GC.03.0009	604.3603099	Organic oxygen compounds
GC.03.0010	689.5111293	Organic oxygen compounds
GC.03.0011	905.3270492	NA
GC.03.0012	618.4773554	NA
GC.03.0013	973.4837304	Organic oxygen compounds
GC.03.0014	426.7172661	Organic acids and derivatives
GC.03.0015	554.5475926	Lipids and lipid-like molecules
GC.03.0016	582.2242427	Organic oxygen compounds
GC.03.0017	913.19679	NA
GC.03.0018	235.2794138	Homogeneous non-metal compounds
GC.03.0019	512.4669012	Organic acids and derivatives
GC.03.0020	526.9852785	NA
GC.03.0021	416.0852179	NA
GC.03.0022	720.6606712	Lipids and lipid-like molecules
GC.03.0023	317.2797887	Homogeneous non-metal compounds
GC.03.0024	578.4344783	Organic acids and derivatives
GC.03.0025	873.5896212	NA
GC.03.0026	488.1969242	Organic acids and derivatives

Compound	Retention Time	Super class
GC.03.0027	785.2558571	NA
GC.03.0029	265.8842459	Organic acids and derivatives
GC.03.0030	615.115	Organic oxygen compounds
GC.03.0031	1079.578789	NA
GC.03.0032	873.6081964	NA
GC.03.0033	913.213625	NA
GC.03.0034	722.9986364	Organic acids and derivatives
GC.03.0035	973.4794375	NA
GC.03.0036	317.1070217	Organic oxygen compounds
GC.03.0037	613.7480435	Organic oxygen compounds
GC.03.0038	905.3105455	NA
GC.03.0039	615.0139545	NA
GC.03.0040	517.92075	NA
GC.03.0041	317.229119	Organic oxygen compounds
GC.03.0042	731.4316829	NA
GC.03.0043	628.381475	Organic oxygen compounds
GC.03.0044	543.4971	NA
GC.03.0046	893.4089487	NA
GC.03.0047	604.6133421	NA
GC.03.0048	604.368	Organic acids and derivatives
GC.03.0050	689.4509459	NA
GC.03.0051	803.7083514	Organic oxygen compounds
GC.03.0052	659.4322222	Lipids and lipid-like molecules
GC.03.0053	263.9096765	NA
GC.03.0054	905.4049091	NA
GC.03.0055	442.8898438	Organic acids and derivatives
GC.03.0056	622.9176129	NA

Compound	Retention Time	Super class
GC.03.0057	607.9156774	Organoheterocyclic compounds
GC.03.0058	412.8580323	NA
GC.03.0059	704.0695484	NA
GC.03.0060	659.3991	NA
GC.03.0061	350.137	NA
GC.03.0062	761.0765333	NA
GC.03.0063	628.8161724	NA
GC.03.0064	392.5553448	NA
GC.03.0066	338.6081111	NA
GC.03.0067	641.2526667	NA
GC.03.0069	873.5831923	NA
GC.03.0070	647.3682692	NA
GC.03.0071	618.4015385	NA
GC.03.0072	628.4966923	NA
GC.03.0073	1079.40664	NA
GC.03.0075	189.03824	NA
GC.03.0076	623.17728	NA
GC.03.0077	467.44088	NA
GC.03.0078	731.15488	Organoheterocyclic compounds
GC.03.0079	1079.38425	NA
GC.03.0080	608.1451667	NA
GC.03.0081	531.9253333	NA
GC.03.0082	562.0827083	NA
GC.03.0083	740.2407083	NA
GC.03.0084	792.018625	NA
GC.03.0085	1079.578957	NA
GC.03.0086	631.8909565	Organic acids and derivatives

Compound	Retention Time	Super class
GC.03.0087	785.402	NA
GC.03.0088	209.7632273	NA
GC.03.0089	631.0757273	NA
GC.03.0090	427.4815	NA
GC.03.0091	721.4842381	Lipids and lipid-like molecules
GC.03.0092	873.5687	NA
GC.03.0094	513.1085	Organic oxygen compounds
GC.03.0095	233.8148947	NA
GC.03.0096	235.2594211	Homogeneous non-metal compounds
GC.03.0097	606.3486842	NA
GC.03.0098	722.9605789	NA
GC.03.0099	898.6029444	NA
GC.03.0100	246.4493889	NA
GC.03.0101	246.5315556	NA
GC.03.0102	664.4071667	NA
GC.03.0103	615.0996111	NA
GC.03.0104	432.1025556	Organic acids and derivatives
GC.03.0105	572.2538333	NA
GC.03.0106	864.1787778	NA
GC.03.0107	206.4792941	NA
GC.03.0108	188.7794118	NA
GC.03.0109	308.4041765	NA
GC.03.0110	284.8552353	NA
GC.03.0111	596.7862941	NA
GC.03.0112	381.0474118	NA
GC.03.0113	496.5770588	Organic acids and derivatives
GC.03.0114	460.4919412	NA

Compound	Retention Time	Super class
GC.03.0115	561.9962353	NA
GC.03.0117	595.261625	NA
GC.03.0118	485.7451875	NA
GC.03.0119	582.1916875	NA
GC.03.0120	832.465	NA
GC.03.0121	203.3900667	NA
GC.03.0123	240.4784	NA
GC.03.0124	335.339	NA
GC.03.0125	263.3005333	NA
GC.03.0126	656.6228667	NA
GC.03.0127	618.1748667	Organoheterocyclic compounds
GC.03.0128	630.5423333	NA
GC.03.0129	634.2838667	Organic oxygen compounds
GC.03.0131	444.79	NA
GC.03.0132	582.3156667	NA
GC.03.0133	553.9890667	Organic oxygen compounds
GC.03.0134	942.3575	NA
GC.03.0135	892.7530714	NA
GC.03.0136	335.219	Organic acids and derivatives
GC.03.0137	679.1025	NA
GC.03.0138	618.2345	Organic acids and derivatives
GC.03.0139	604.5229286	NA
GC.03.0140	377.0225	Organic acids and derivatives
GC.03.0141	413.0057143	NA
GC.03.0143	819.23	NA
GC.03.0144	1101.444846	NA
GC.03.0145	972.3684615	NA

Compound	Retention Time	Super class
GC.03.0146	873.6438462	NA
GC.03.0147	879.5807692	NA
GC.03.0148	940.225	NA
GC.03.0149	194.9468462	Organoheterocyclic compounds
GC.03.0150	246.4186923	NA
GC.03.0152	301.2323846	NA
GC.03.0153	293.6822308	NA
GC.03.0154	628.313	NA
GC.03.0156	427.171	NA
GC.03.0157	443.808	Organic acids and derivatives
GC.03.0158	476.6702308	Organic acids and derivatives
GC.03.0161	968.5646667	NA
GC.03.0162	972.1901667	NA
GC.03.0163	917.8673333	NA
GC.03.0165	227.0151667	NA
GC.03.0166	284.8361667	NA
GC.03.0167	614.381	NA
GC.03.0168	618.1998333	NA
GC.03.0169	604.8261667	NA
GC.03.0170	598.7845	NA
GC.03.0171	476.1353333	Organic acids and derivatives
GC.03.0172	472.0796667	NA
GC.03.0173	689.3560833	Organoheterocyclic compounds
GC.03.0174	710.2935	NA
GC.03.0175	767.903	NA
GC.03.0176	807.1899167	NA
GC.03.0178	1092.359727	NA

Compound	Retention Time	Super class
GC.03.0179	181.0630909	NA
GC.03.0180	673.1062727	NA
GC.03.0181	604.7054545	NA
GC.03.0182	609.2276364	NA
GC.03.0183	608.609	NA
GC.03.0184	598.5461818	NA
GC.03.0185	731.5755455	NA
GC.03.0186	760.5769091	NA
GC.03.0187	1004.9993	NA
GC.03.0189	205.5885	NA
GC.03.0190	313.7486	Organic nitrogen compounds
GC.03.0191	284.7833	NA
GC.03.0192	295.9964	NA
GC.03.0193	670.4067	NA
GC.03.0194	675.2756	NA
GC.03.0195	655.9534	NA
GC.03.0196	625.1102	Organic acids and derivatives
GC.03.0197	635.9493	NA
GC.03.0198	426.6229	NA
GC.03.0200	446.7036	Organic acids and derivatives
GC.03.0201	450.1565	NA
GC.03.0202	461.6541	NA
GC.03.0203	456.0132	NA
GC.03.0204	522.9084	NA
GC.03.0205	814.038	NA
GC.03.0206	819.2587	NA
GC.03.0207	1078.298	NA

Compound	Retention Time	Super class
GC.03.0208	1100.992333	NA
GC.03.0209	1068.961222	NA
GC.03.0210	973.8846667	NA
GC.03.0211	943.3602222	NA
GC.03.0212	909.169	NA
GC.03.0213	326.7267778	Organoheterocyclic compounds
GC.03.0215	360.925	NA
GC.03.0216	488.6055556	NA
GC.03.0217	479.5762222	NA
GC.03.0218	460.9891111	NA
GC.03.0220	730.1925556	Lipids and lipid-like molecules
GC.03.0221	756.8393333	NA
GC.03.0222	788.3192222	NA
GC.03.0223	1084.893875	NA
GC.03.0224	1004.480125	NA
GC.03.0225	973.410375	NA
GC.03.0226	877.066375	NA
GC.03.0227	935.778	NA
GC.03.0228	194.964875	NA
GC.03.0229	249.973125	NA
GC.03.0230	314.811125	NA
GC.03.0231	312.413875	NA
GC.03.0232	622.81	NA
GC.03.0233	622.87675	NA
GC.03.0234	426.713625	NA
GC.03.0235	441.438625	NA
GC.03.0237	577.382	NA

Compound	Retention Time	Super class
GC.03.0238	695.023625	NA
GC.03.0239	725.275125	NA
GC.03.0241	826.794375	NA
GC.03.0242	1051.95	NA
GC.03.0243	1005.120571	NA
GC.03.0244	1025.348	NA
GC.03.0245	996.541	NA
GC.03.0247	228.9065714	NA
GC.03.0248	218.4891429	NA
GC.03.0249	327.7115714	NA
GC.03.0250	673.1548571	NA
GC.03.0251	623.7868571	NA
GC.03.0252	622.707	NA
GC.03.0253	604.2615714	NA
GC.03.0254	363.3385714	Organic acids and derivatives
GC.03.0255	490.4192857	NA
GC.03.0256	442.2804286	NA
GC.03.0257	444.5221429	NA
GC.03.0258	445.0444286	NA
GC.03.0259	477.2072857	NA
GC.03.0260	466.036	Organic acids and derivatives
GC.03.0261	523.5861429	NA
GC.03.0262	565.2338571	NA
GC.03.0263	686.191	NA
GC.03.0264	714.9767143	NA
GC.03.0265	1094.098333	NA
GC.03.0266	1100.736667	NA

Compound	Retention Time	Super class
GC.03.0267	1280.6235	NA
GC.03.0269	987.5375	NA
GC.03.0270	873.631	NA
GC.03.0271	878.1773333	NA
GC.03.0272	877.0715	NA
GC.03.0274	891.6821667	NA
GC.03.0276	194.5426667	NA
GC.03.0277	183.1735	NA
GC.03.0278	247.3016667	NA
GC.03.0279	221.6095	NA
GC.03.0280	259.7858333	NA
GC.03.0281	268.316	NA
GC.03.0282	644.6096667	NA
GC.03.0283	623.0313333	NA
GC.03.0285	631.5475	NA
GC.03.0286	351.7346667	Organic acids and derivatives
GC.03.0287	402.2008333	NA
GC.03.0288	426.8566667	NA
GC.03.0289	443.5591667	NA
GC.03.0290	443.99	NA
GC.03.0291	474.8458333	NA
GC.03.0292	450.0641667	NA
GC.03.0293	512.752	NA
GC.03.0294	512.724	NA
GC.03.0295	558.9558333	NA
GC.03.0296	733.558	NA
GC.03.0297	730.4918333	NA

Compound	Retention Time	Super class
GC.03.0298	800.3888333	NA
GC.03.0299	834.6165	NA
GC.03.0300	863.8686667	NA
GC.03.0301	1079.201	NA
GC.03.0302	1094.0334	NA
GC.03.0303	1130.0212	NA
GC.03.0304	1127.9542	NA
GC.03.0305	1163.1256	NA
GC.03.0306	1244.5964	NA
GC.03.0307	1056.609	NA
GC.03.0310	981.745	NA
GC.03.0311	879.2638	NA
GC.03.0312	893.073	NA
GC.03.0313	195.2622	NA
GC.03.0314	199.7622	Organic acids and derivatives
GC.03.0315	184.9592	NA
GC.03.0316	246.8122	NA
GC.03.0317	246.5346	NA
GC.03.0318	330.6656	NA
GC.03.0319	269.8078	NA
GC.03.0320	296.0504	NA
GC.03.0321	662.4996	NA
GC.03.0322	644.958	NA
GC.03.0323	613.7746	NA
GC.03.0324	622.7488	NA
GC.03.0325	631.1318	NA
GC.03.0326	608.2534	NA

Compound	Retention Time	Super class
GC.03.0327	595.037	NA
GC.03.0328	596.7488	NA
GC.03.0329	388.4514	NA
GC.03.0330	488.2368	NA
GC.03.0331	493.2632	Organic acids and derivatives
GC.03.0332	432.1688	NA
GC.03.0333	433.127	NA
GC.03.0334	439.1348	NA
GC.03.0335	435.684	NA
GC.03.0336	435.3612	NA
GC.03.0337	459.0384	NA
GC.03.0338	519.0626	NA
GC.03.0340	551.7124	NA
GC.03.0343	717.1176	NA
GC.03.0344	715.5446	NA
GC.03.0345	739.7466	NA
GC.03.0347	762.1498	NA
GC.03.0348	754.2912	NA
GC.03.0350	863.9002	NA
GC.03.0351	840.9386	NA
GC.03.0352	1082.608	NA
GC.03.0353	1124.36225	NA
GC.03.0354	1136.354	NA
GC.03.0355	1280.7175	NA
GC.03.0356	1025.72525	NA
GC.03.0358	966.374	NA
GC.03.0359	988.6985	NA

Compound	Retention Time	Super class
GC.03.0360	981.723	NA
GC.03.0361	873.561	NA
GC.03.0362	883.904	NA
GC.03.0365	883.28175	NA
GC.03.0366	886.9605	NA
GC.03.0367	934.69775	NA
GC.03.0368	891.4185	NA
GC.03.0370	920.947	NA
GC.03.0371	210.04125	NA
GC.03.0372	191.25025	NA
GC.03.0373	254.05275	NA
GC.03.0375	246.93825	NA
GC.03.0376	246.54625	NA
GC.03.0377	249.331	NA
GC.03.0378	223.194	NA
GC.03.0379	314.111	NA
GC.03.0380	302.46375	NA
GC.03.0381	296.22475	NA
GC.03.0382	675.47575	NA
GC.03.0383	674.29525	NA
GC.03.0384	665.9075	NA
GC.03.0385	655.71375	NA
GC.03.0386	645.0745	Organoheterocyclic compounds
GC.03.0387	637.2755	NA
GC.03.0388	605.3515	NA
GC.03.0389	363.18825	Organic acids and derivatives
GC.03.0390	365.58475	Organoheterocyclic compounds

Compound	Retention Time	Super class
GC.03.0391	378.51625	NA
GC.03.0393	389.81175	NA
GC.03.0394	391.336	NA
GC.03.0397	480.44975	NA
GC.03.0398	482.176	NA
GC.03.0399	471.09075	Organic acids and derivatives
GC.03.0400	464.094	NA
GC.03.0401	457.2385	NA
GC.03.0402	536.88525	NA
GC.03.0403	543.9925	NA
GC.03.0404	582.9045	NA
GC.03.0406	560.33375	NA
GC.03.0408	707.4015	NA
GC.03.0409	702.86125	NA
GC.03.0410	715.51	NA
GC.03.0411	724.2105	NA
GC.03.0412	748.74025	NA
GC.03.0413	736.8835	NA
GC.03.0414	766.417	NA
GC.03.0415	798.72375	NA
GC.03.0416	843.2145	NA
GC.03.0417	851.511	NA
GC.03.0418	1081.232333	NA
GC.03.0419	1086.226667	NA
GC.03.0420	1083.263	NA
GC.03.0421	1089.906333	NA
GC.03.0423	1102.091	NA

Compound	Retention Time	Super class
GC.03.0424	1123.993	NA
GC.03.0428	1107.434333	NA
GC.03.0430	1137.665	NA
GC.03.0431	1156.337667	NA
GC.03.0433	1262.054	NA
GC.03.0434	1319.258	NA
GC.03.0437	1060.916333	NA
GC.03.0439	1052.597	NA
GC.03.0441	1038.898333	NA
GC.03.0442	1040.258667	NA
GC.03.0443	1034.104	NA
GC.03.0445	962.382	NA
GC.03.0452	882.0203333	NA
GC.03.0453	938.6636667	NA
GC.03.0454	940.42	NA
GC.03.0455	893.1216667	NA
GC.03.0456	917.2693333	NA
GC.03.0457	921.737	NA
GC.03.0458	920.0956667	NA
GC.03.0459	208.616	NA
GC.03.0460	182.455	NA
GC.03.0461	188.8603333	NA
GC.03.0462	235.7036667	NA
GC.03.0463	323.4	NA
GC.03.0464	275.1286667	NA
GC.03.0465	281.1256667	NA
GC.03.0466	291.202	NA

Compound	Retention Time	Super class
GC.03.0467	668.8576667	NA
GC.03.0468	641.913	NA
GC.03.0469	640.053	NA
GC.03.0471	621.667	Nucleosides, nucleotides, and analogues
GC.03.0473	347.1076667	NA
GC.03.0474	355.275	NA
GC.03.0475	372.5243333	NA
GC.03.0476	396.2766667	NA
GC.03.0478	420.5663333	NA
GC.03.0480	497.2276667	NA
GC.03.0481	443.8643333	NA
GC.03.0482	432.495	NA
GC.03.0483	437.1293333	NA
GC.03.0484	470.0406667	NA
GC.03.0485	453.0446667	NA
GC.03.0486	534.1453333	NA
GC.03.0488	547.064	NA
GC.03.0491	586.896	NA
GC.03.0492	584.338	NA
GC.03.0493	565.8193333	NA
GC.03.0494	554.077	NA
GC.03.0495	555.001	NA
GC.03.0496	556.6086667	NA
GC.03.0498	727.9223333	NA
GC.03.0500	806.621	NA
GC.03.0501	803.4856667	NA
GC.03.0502	796.653	NA

Compound	Retention Time	Super class
GC.03.0503	794.8743333	NA
GC.03.0505	1507.061	NA
GC.03.0506	1505.7175	NA
GC.03.0510	1447.731	NA
GC.03.0511	1453.019	NA
GC.03.0514	1440.88	NA
GC.03.0517	1499.0775	NA
GC.03.0518	1497.005	NA
GC.03.0520	1081.8115	NA
GC.03.0521	1080.897	NA
GC.03.0522	1080.568	NA
GC.03.0523	1080.5895	NA
GC.03.0524	1079.4165	NA
GC.03.0525	1087.061	NA
GC.03.0528	1087.2835	NA
GC.03.0531	1085.512	NA
GC.03.0532	1084.3415	NA
GC.03.0533	1090.57	NA
GC.03.0535	1094.228	NA
GC.03.0537	1094.451	NA
GC.03.0540	1094.8665	NA
GC.03.0543	1092.308	NA
GC.03.0545	1091.455	NA
GC.03.0548	1100.317	NA
GC.03.0549	1099.996	NA
GC.03.0550	1100.124	NA
GC.03.0551	1101.866	NA

Compound	Retention Time	Super class
GC.03.0552	1101.911	NA
GC.03.0556	1101.3125	NA
GC.03.0557	1129.5605	NA
GC.03.0559	1122.5225	NA
GC.03.0561	1127.4445	NA
GC.03.0562	1125.1655	NA
GC.03.0564	1126.174	NA
GC.03.0565	1119.4915	NA
GC.03.0567	1114.5935	NA
GC.03.0568	1113.4565	NA
GC.03.0569	1114.053	NA
GC.03.0571	1112.0295	NA
GC.03.0573	1108.224	NA
GC.03.0574	1108.3875	NA
GC.03.0576	1136.4615	NA
GC.03.0577	1136.1075	NA
GC.03.0578	1136.3315	NA
GC.03.0579	1135.3305	NA
GC.03.0580	1135.984	NA
GC.03.0582	1138.107	NA
GC.03.0583	1137.66	NA
GC.03.0584	1137.0845	NA
GC.03.0585	1136.7075	NA
GC.03.0590	1149.0065	NA
GC.03.0591	1148.9255	NA
GC.03.0592	1148.008	NA
GC.03.0593	1151.455	NA

Compound	Retention Time	Super class
GC.03.0594	1149.724	NA
GC.03.0596	1165.0225	NA
GC.03.0598	1161.359	NA
GC.03.0599	1161.627	NA
GC.03.0603	1154.6745	NA
GC.03.0604	1168.353	NA
GC.03.0605	1170.2275	NA
GC.03.0607	1185.3105	NA
GC.03.0609	1182.9055	NA
GC.03.0612	1225.5135	NA
GC.03.0613	1226.596	NA
GC.03.0616	1203.4205	NA
GC.03.0621	1245.6655	NA
GC.03.0623	1280.7505	NA
GC.03.0626	1311.237	NA
GC.03.0627	1314.4905	NA
GC.03.0628	1322.559	NA
GC.03.0629	1327.3725	NA
GC.03.0632	1302.2585	NA
GC.03.0634	1373.1765	NA
GC.03.0635	1369.5795	NA
GC.03.0637	1339.252	NA
GC.03.0641	1064.5965	NA
GC.03.0642	1065.136	NA
GC.03.0643	1066.8795	NA
GC.03.0644	1056.5305	NA
GC.03.0645	1051.8145	Nucleosides, nucleotides, and analogues

Compound	Retention Time	Super class
GC.03.0646	1053.913	NA
GC.03.0647	1008.8785	NA
GC.03.0648	1005.6365	NA
GC.03.0649	1006.284	NA
GC.03.0650	1006.7795	NA
GC.03.0651	1005.3445	NA
GC.03.0653	1013.815	NA
GC.03.0658	1025.9895	NA
GC.03.0659	1026.5455	NA
GC.03.0661	1028.216	NA
GC.03.0662	1031.3965	NA
GC.03.0663	1041.6035	NA
GC.03.0666	951.3515	NA
GC.03.0667	950.025	NA
GC.03.0670	963.062	NA
GC.03.0671	956.2795	NA
GC.03.0672	956.9945	NA
GC.03.0673	966.9	NA
GC.03.0678	972.179	NA
GC.03.0680	973.3825	NA
GC.03.0682	991.6155	NA
GC.03.0683	978.3865	NA
GC.03.0684	985.1345	NA
GC.03.0686	985.4665	NA
GC.03.0688	984.259	NA
GC.03.0689	984.065	NA
GC.03.0692	981.8155	NA

Compound	Retention Time	Super class
GC.03.0693	981.8805	NA
GC.03.0696	980.0325	NA
GC.03.0698	979.784	NA
GC.03.0699	873.5935	NA
GC.03.0702	884.332	NA
GC.03.0703	883.281	Phenylpropanoids and polyketides
GC.03.0707	933.543	NA
GC.03.0708	943.0695	NA
GC.03.0709	939.6265	NA
GC.03.0713	893.4225	NA
GC.03.0714	894.408	NA
GC.03.0715	895.2145	NA
GC.03.0716	900.8085	NA
GC.03.0717	898.6835	NA
GC.03.0718	909.483	NA
GC.03.0720	929.686	NA
GC.03.0722	198.202	NA
GC.03.0723	181.0715	NA
GC.03.0725	232.982	NA
GC.03.0727	257.821	NA
GC.03.0728	286.482	NA
GC.03.0729	680.6805	Phenylpropanoids and polyketides
GC.03.0731	660.1215	NA
GC.03.0734	614.445	NA
GC.03.0735	618.2345	NA
GC.03.0736	630.2255	NA
GC.03.0737	638.0565	NA

Compound	Retention Time	Super class
GC.03.0738	604.3975	NA
GC.03.0739	592.343	NA
GC.03.0740	353.5505	NA
GC.03.0741	378.55	NA
GC.03.0743	389.1205	NA
GC.03.0745	400.76	NA
GC.03.0746	406.86	NA
GC.03.0747	413.1325	NA
GC.03.0748	415.691	NA
GC.03.0749	429.02	NA
GC.03.0751	499.4625	NA
GC.03.0753	438.068	NA
GC.03.0754	527.18	NA
GC.03.0755	523.557	NA
GC.03.0756	536.612	NA
GC.03.0757	569.6815	NA
GC.03.0762	700.75	NA
GC.03.0763	715.4665	NA
GC.03.0764	715.4805	NA
GC.03.0765	714.951	NA
GC.03.0766	725.451	NA
GC.03.0768	747.856	NA
GC.03.0769	748.942	NA
GC.03.0771	740.136	NA
GC.03.0774	762.131	NA
GC.03.0776	753.5765	NA
GC.03.0779	831.594	NA

Compound	Retention Time	Super class
GC.03.0780	833.7625	NA
GC.03.0781	828.3125	NA
GC.03.0782	812.4275	NA
GC.03.0783	814.0175	NA
GC.03.0784	818.905	NA
GC.03.0786	865.552	NA
GC.03.0787	864.103	NA
GC.03.0788	842.8385	NA
GC.03.0790	853.7805	NA
GC.03.0791	856.4775	NA
GC.03.0792	847.2415	NA
LC.03.0001	342.2560634	Lipids and lipid-like molecules
LC.03.0002	473.8421209	NA
LC.03.0003	749.0235172	Lipids and lipid-like molecules
LC.03.0004	188.265942	Lipids and lipid-like molecules
LC.03.0005	342.285	NA
LC.03.0006	689.0814667	NA
LC.03.0007	680.6349322	Lipids and lipid-like molecules
LC.03.0008	498.8051897	NA
LC.03.0009	550.7769649	Lipids and lipid-like molecules
LC.03.0010	18.09253571	NA
LC.03.0011	476.60474	Lipids and lipid-like molecules
LC.03.0012	484.5197755	Lipids and lipid-like molecules
LC.03.0013	200.7148333	NA
LC.03.0014	521.5479333	Lipids and lipid-like molecules
LC.03.0015	23.47621951	Organic oxygen compounds
LC.03.0016	704.3701795	Lipids and lipid-like molecules

Compound	Retention Time	Super class
LC.03.0017	711.3491795	Lipids and lipid-like molecules
LC.03.0018	224.5752105	NA
LC.03.0019	311.2088421	Lipids and lipid-like molecules
LC.03.0020	588.0414865	Benzenoids
LC.03.0022	696.0413243	NA
LC.03.0023	187.6288108	Organic acids and derivatives
LC.03.0024	338.1327222	Lipids and lipid-like molecules
LC.03.0025	17.98617143	NA
LC.03.0026	618.6593714	NA
LC.03.0027	857.9161143	Lipids and lipid-like molecules
LC.03.0028	761.6957714	Lipids and lipid-like molecules
LC.03.0031	195.5017143	NA
LC.03.0032	608.8271765	Organoheterocyclic compounds
LC.03.0033	863.9952059	Lipids and lipid-like molecules
LC.03.0034	388.1878529	Lipids and lipid-like molecules
LC.03.0036	659.5264545	NA
LC.03.0037	383.9499688	NA
LC.03.0038	612.4354516	Organoheterocyclic compounds
LC.03.0039	443.2282581	Lipids and lipid-like molecules
LC.03.0040	496.2920968	Lipids and lipid-like molecules
LC.03.0041	18.04603333	NA
LC.03.0043	727.6321	Phenylpropanoids and polyketides
LC.03.0044	756.8296	Lipids and lipid-like molecules
LC.03.0045	452.6413	Lipids and lipid-like molecules
LC.03.0046	23.55168966	Organic oxygen compounds
LC.03.0047	1005.00969	NA
LC.03.0048	23.62496296	Organoheterocyclic compounds

Compound	Retention Time	Super class
LC.03.0049	773.9751481	Lipids and lipid-like molecules
LC.03.0050	397.0408519	Lipids and lipid-like molecules
LC.03.0051	394.4412222	Lipids and lipid-like molecules
LC.03.0052	349.1564815	Lipids and lipid-like molecules
LC.03.0053	840.1081154	Lipids and lipid-like molecules
LC.03.0054	764.8521923	Lipids and lipid-like molecules
LC.03.0055	286.7376154	Lignans, neolignans and related compounds
LC.03.0056	840.90832	Lipids and lipid-like molecules
LC.03.0057	846.69404	Lipids and lipid-like molecules
LC.03.0058	855.2888	Lipids and lipid-like molecules
LC.03.0059	749.00476	Lipids and lipid-like molecules
LC.03.0060	250.90144	Phenylpropanoids and polyketides
LC.03.0061	338.16	Lipids and lipid-like molecules
LC.03.0062	587.6154167	Lipids and lipid-like molecules
LC.03.0063	578.7604167	Organoheterocyclic compounds
LC.03.0064	830.2118333	NA
LC.03.0066	422.3746522	Lipids and lipid-like molecules
LC.03.0067	457.368913	Lipids and lipid-like molecules
LC.03.0068	578.943	Hydrocarbons
LC.03.0069	709.7594091	Lipids and lipid-like molecules
LC.03.0070	170.8471818	NA
LC.03.0071	583.1299524	Lipids and lipid-like molecules
LC.03.0072	642.0361429	NA
LC.03.0073	849.1078571	Lipids and lipid-like molecules
LC.03.0075	755.4849524	Lipids and lipid-like molecules
LC.03.0076	774.0224286	Lipids and lipid-like molecules
LC.03.0077	777.0487619	Lipids and lipid-like molecules

Compound	Retention Time	Super class
LC.03.0078	469.4495714	Lipids and lipid-like molecules
LC.03.0079	482.123381	Lipids and lipid-like molecules
LC.03.0080	716.7721	Lipids and lipid-like molecules
LC.03.0081	748.8587	Lipids and lipid-like molecules
LC.03.0082	466.45205	Lipids and lipid-like molecules
LC.03.0083	22.64789474	Lipids and lipid-like molecules
LC.03.0084	618.0843158	Organic oxygen compounds
LC.03.0085	666.2212632	Lipids and lipid-like molecules
LC.03.0087	744.8166842	Lipids and lipid-like molecules
LC.03.0088	749.2202632	Organic oxygen compounds
LC.03.0089	737.9352632	Lipids and lipid-like molecules
LC.03.0090	768.1645789	Lipids and lipid-like molecules
LC.03.0092	188.4445263	NA
LC.03.0093	170.1322632	NA
LC.03.0094	23.52938889	Organic oxygen compounds
LC.03.0095	23.52188889	NA
LC.03.0096	18.11633333	Phenylpropanoids and polyketides
LC.03.0097	618.3228333	NA
LC.03.0098	834.5708333	Lipids and lipid-like molecules
LC.03.0099	788.1256667	NA
LC.03.0100	774.9932778	Lipids and lipid-like molecules
LC.03.0101	770.8487778	NA
LC.03.0102	474.7701667	NA
LC.03.0103	522.9615556	Lipids and lipid-like molecules
LC.03.0104	319.1828889	Lipids and lipid-like molecules
LC.03.0105	339.8824444	Organic acids and derivatives
LC.03.0106	867.3052941	NA

Compound	Retention Time	Super class
LC.03.0107	702.1449412	Lipids and lipid-like molecules
LC.03.0109	726.1416471	Phenylpropanoids and polyketides
LC.03.0110	737.1485294	Lipids and lipid-like molecules
LC.03.0111	761.3968235	Lipids and lipid-like molecules
LC.03.0112	476.599	Lipids and lipid-like molecules
LC.03.0113	250.8772941	Lignans, neolignans and related compounds
LC.03.0114	302.3903529	Phenylpropanoids and polyketides
LC.03.0115	59.9169375	Phenylpropanoids and polyketides
LC.03.0116	582.3154375	NA
LC.03.0117	667.545875	NA
LC.03.0118	675.0288125	Lipids and lipid-like molecules
LC.03.0119	791.113375	NA
LC.03.0120	389.929625	Lipids and lipid-like molecules
LC.03.0121	476.9790625	Lipids and lipid-like molecules
LC.03.0122	482.27875	Lipids and lipid-like molecules
LC.03.0123	200.9040625	NA
LC.03.0124	307.28825	NA
LC.03.0126	22.8896	Organic acids and derivatives
LC.03.0127	23.24886667	Lipids and lipid-like molecules
LC.03.0128	604.8748667	Lipids and lipid-like molecules
LC.03.0129	823.9682	NA
LC.03.0130	823.7772	Lipids and lipid-like molecules
LC.03.0132	728.1604	Lipids and lipid-like molecules
LC.03.0133	805.7139333	Lipids and lipid-like molecules
LC.03.0134	758.4136667	Organic oxygen compounds
LC.03.0135	756.7864667	Hydrocarbons
LC.03.0136	771.1774667	Lipids and lipid-like molecules

Compound	Retention Time	Super class
LC.03.0137	770.5926667	Organic oxygen compounds
LC.03.0138	780.9278667	NA
LC.03.0139	474.3232667	Lipids and lipid-like molecules
LC.03.0140	550.0128	Lipids and lipid-like molecules
LC.03.0141	186.1378667	Phenylpropanoids and polyketides
LC.03.0142	630.1544286	Organic acids and derivatives
LC.03.0143	878.5515	Lipids and lipid-like molecules
LC.03.0145	837.2661429	Lipids and lipid-like molecules
LC.03.0146	867.7555714	Lipids and lipid-like molecules
LC.03.0147	852.7403571	NA
LC.03.0148	724.0943571	Lipids and lipid-like molecules
LC.03.0149	735.0747857	Lipids and lipid-like molecules
LC.03.0150	759.1358571	Lipids and lipid-like molecules
LC.03.0151	756.8590714	Lipids and lipid-like molecules
LC.03.0152	769.183	Lipids and lipid-like molecules
LC.03.0153	386.1592857	Lipids and lipid-like molecules
LC.03.0154	395.2845714	Lipids and lipid-like molecules
LC.03.0155	449.8193571	Lipids and lipid-like molecules
LC.03.0156	454.2207857	Alkaloids and derivatives
LC.03.0157	468.9525	Lipids and lipid-like molecules
LC.03.0158	536.6536429	Lipids and lipid-like molecules
LC.03.0159	192.8697143	NA
LC.03.0160	204.811	NA
LC.03.0161	18.19107692	Organic acids and derivatives
LC.03.0162	584.9566154	Lipids and lipid-like molecules
LC.03.0163	572.924	Lipids and lipid-like molecules
LC.03.0164	572.2059231	Lipids and lipid-like molecules

Compound	Retention Time	Super class
LC.03.0165	618.4393846	Organoheterocyclic compounds
LC.03.0167	830.4668462	Lipids and lipid-like molecules
LC.03.0169	862.1353846	Lipids and lipid-like molecules
LC.03.0170	869.2729231	Lipids and lipid-like molecules
LC.03.0171	716.7555385	Lipids and lipid-like molecules
LC.03.0172	727.6268462	NA
LC.03.0173	732.3497692	Lipids and lipid-like molecules
LC.03.0174	751.8183077	Organic oxygen compounds
LC.03.0175	756.4976923	NA
LC.03.0176	493.9808462	Phenylpropanoids and polyketides
LC.03.0177	554.8421538	Organoheterocyclic compounds
LC.03.0178	186.7520769	NA
LC.03.0179	23.56991667	Organic oxygen compounds
LC.03.0180	23.19658333	NA
LC.03.0181	22.43183333	Lipids and lipid-like molecules
LC.03.0182	23.51608333	Organic oxygen compounds
LC.03.0183	16.91475	NA
LC.03.0184	18.10175	NA
LC.03.0185	596.5398333	Lipids and lipid-like molecules
LC.03.0186	572.1825833	Lipids and lipid-like molecules
LC.03.0187	572.17425	Lipids and lipid-like molecules
LC.03.0189	611.8813333	Organic acids and derivatives
LC.03.0190	614.5425833	NA
LC.03.0191	614.1424167	Organoheterocyclic compounds
LC.03.0192	873.58725	Organic oxygen compounds
LC.03.0193	844.99725	Lipids and lipid-like molecules
LC.03.0194	675.6195833	Organic oxygen compounds

Compound	Retention Time	Super class
LC.03.0195	804.6174167	NA
LC.03.0196	762.8588333	NA
LC.03.0197	767.4145833	NA
LC.03.0198	786.3258333	Organic oxygen compounds
LC.03.0199	780.8195	NA
LC.03.0200	402.65675	Lipids and lipid-like molecules
LC.03.0201	447.5168333	NA
LC.03.0202	457.0085833	Phenylpropanoids and polyketides
LC.03.0203	517.6128333	Organic nitrogen compounds
LC.03.0204	188.7555833	Organic acids and derivatives
LC.03.0205	1000.34575	Lipids and lipid-like molecules
LC.03.0206	586.9548182	Organic Polymers
LC.03.0207	578.4074545	NA
LC.03.0208	631.8280909	Lipids and lipid-like molecules
LC.03.0209	630.5431818	Organoheterocyclic compounds
LC.03.0210	617.3987273	Lipids and lipid-like molecules
LC.03.0211	651.9716364	Lipids and lipid-like molecules
LC.03.0212	647.063	Lipids and lipid-like molecules
LC.03.0213	646.178	NA
LC.03.0215	829.3309091	NA
LC.03.0217	713.4532727	Lipids and lipid-like molecules
LC.03.0218	677.6634545	Lipids and lipid-like molecules
LC.03.0219	691.7984545	NA
LC.03.0220	724.8190909	Lipids and lipid-like molecules
LC.03.0221	727.4273636	Lipids and lipid-like molecules
LC.03.0222	738.7501818	Organic oxygen compounds
LC.03.0223	735.3040909	Lipids and lipid-like molecules

Compound	Retention Time	Super class
LC.03.0227	544.0159091	Lipids and lipid-like molecules
LC.03.0228	186.254	Organic oxygen compounds
LC.03.0229	34.6456	NA
LC.03.0230	589.5901	NA
LC.03.0232	575.3978	Phenylpropanoids and polyketides
LC.03.0233	632.0601	Lipids and lipid-like molecules
LC.03.0234	666.1024	Lipids and lipid-like molecules
LC.03.0235	645.1089	Organic acids and derivatives
LC.03.0236	817.5262	NA
LC.03.0237	826.7764	Organic oxygen compounds
LC.03.0238	834.3775	Lipids and lipid-like molecules
LC.03.0239	711.9875	Lipids and lipid-like molecules
LC.03.0240	717.5192	Lipids and lipid-like molecules
LC.03.0241	685.3476	Lipids and lipid-like molecules
LC.03.0243	724.6249	Lipids and lipid-like molecules
LC.03.0244	727.8339	NA
LC.03.0245	805.9303	Lipids and lipid-like molecules
LC.03.0246	799.0817	NA
LC.03.0247	791.9629	Organic oxygen compounds
LC.03.0248	772.0325	Lipids and lipid-like molecules
LC.03.0249	778.0164	NA
LC.03.0250	447.2739	Lipids and lipid-like molecules
LC.03.0251	488.9638	Lipids and lipid-like molecules
LC.03.0252	187.4836	NA
LC.03.0253	185.0866	NA
LC.03.0254	239.0817	Organic acids and derivatives
LC.03.0255	293.6246	Phenylpropanoids and polyketides

Compound	Retention Time	Super class
LC.03.0256	358.0024	Organoheterocyclic compounds
LC.03.0257	23.84077778	Benzenoids
LC.03.0258	22.92044444	Organic acids and derivatives
LC.03.0259	52.77844444	Phenylpropanoids and polyketides
LC.03.0260	588.8681111	Lipids and lipid-like molecules
LC.03.0261	583.4883333	Organic acids and derivatives
LC.03.0262	636.9371111	Lipids and lipid-like molecules
LC.03.0263	635.4705556	Benzenoids
LC.03.0264	620.1863333	Lipids and lipid-like molecules
LC.03.0265	625.0436667	NA
LC.03.0266	625.1093333	Organic acids and derivatives
LC.03.0267	610.9373333	Lipids and lipid-like molecules
LC.03.0268	662.2994444	NA
LC.03.0269	667.4981111	Lipids and lipid-like molecules
LC.03.0270	876.4415556	Lipids and lipid-like molecules
LC.03.0271	881.0627778	Lipids and lipid-like molecules
LC.03.0272	891.0606667	NA
LC.03.0273	837.526	Lipids and lipid-like molecules
LC.03.0274	844.2912222	NA
LC.03.0275	847.5341111	NA
LC.03.0276	858.8853333	NA
LC.03.0277	854.1113333	NA
LC.03.0280	725.6763333	Lipids and lipid-like molecules
LC.03.0281	740.4885556	NA
LC.03.0282	742.6215556	Lipids and lipid-like molecules
LC.03.0283	742.3885556	NA
LC.03.0284	740.0711111	NA

Compound	Retention Time	Super class
LC.03.0285	737.6944444	Lipids and lipid-like molecules
LC.03.0286	798.7516667	Lipids and lipid-like molecules
LC.03.0287	761.4001111	NA
LC.03.0288	764.9797778	NA
LC.03.0289	787.2145556	Lipids and lipid-like molecules
LC.03.0291	789.7938889	NA
LC.03.0292	772.1188889	NA
LC.03.0293	769.198	Lipids and lipid-like molecules
LC.03.0295	784.8697778	NA
LC.03.0296	778.2196667	Lipids and lipid-like molecules
LC.03.0297	778.5271111	Lipids and lipid-like molecules
LC.03.0298	401.025	NA
LC.03.0299	409.4957778	NA
LC.03.0300	409.4	Lipids and lipid-like molecules
LC.03.0301	500.1615556	NA
LC.03.0302	474.843	NA
LC.03.0303	482.8468889	Lipids and lipid-like molecules
LC.03.0304	186.721	NA
LC.03.0305	188.8614444	NA
LC.03.0306	193.4805556	NA
LC.03.0307	195.4077778	NA
LC.03.0308	178.2732222	NA
LC.03.0309	260.7042222	NA
LC.03.0310	342.4417778	Lipids and lipid-like molecules
LC.03.0311	1003.346	NA
LC.03.0312	31.269375	NA
LC.03.0313	23.68525	NA

Compound	Retention Time	Super class
LC.03.0314	23.628375	Lipids and lipid-like molecules
LC.03.0315	23.086125	Alkaloids and derivatives
LC.03.0316	17.063375	NA
LC.03.0317	18.045625	NA
LC.03.0318	44.0005	NA
LC.03.0319	579.70025	Lipids and lipid-like molecules
LC.03.0320	578.35275	NA
LC.03.0321	629.95125	NA
LC.03.0322	617.200875	NA
LC.03.0323	623.798875	NA
LC.03.0324	604.82875	NA
LC.03.0325	610.9585	NA
LC.03.0326	659.30625	NA
LC.03.0327	653.464125	NA
LC.03.0328	645.15775	NA
LC.03.0329	641.238625	NA
LC.03.0330	819.352125	NA
LC.03.0332	828.511875	NA
LC.03.0333	823.946375	NA
LC.03.0335	842.074125	NA
LC.03.0336	833.45425	Lipids and lipid-like molecules
LC.03.0339	696.6665	NA
LC.03.0340	730.9925	NA
LC.03.0341	746.408875	NA
LC.03.0342	751.85175	Lipids and lipid-like molecules
LC.03.0343	805.675	NA
LC.03.0344	764.796875	Lipids and lipid-like molecules

Compound	Retention Time	Super class
LC.03.0345	756.435375	NA
LC.03.0346	756.716625	Lipids and lipid-like molecules
LC.03.0347	790.313	NA
LC.03.0348	770.76975	NA
LC.03.0349	772.26325	NA
LC.03.0351	781.567125	Lipids and lipid-like molecules
LC.03.0352	780.097375	NA
LC.03.0353	778.159	NA
LC.03.0354	777.33775	Lipids and lipid-like molecules
LC.03.0355	777.36775	NA
LC.03.0356	380.171375	NA
LC.03.0357	397.628125	Lipids and lipid-like molecules
LC.03.0358	456.90625	Lipids and lipid-like molecules
LC.03.0359	502.271625	Lipids and lipid-like molecules
LC.03.0360	467.973375	NA
LC.03.0361	551.159125	Organoheterocyclic compounds
LC.03.0362	184.440375	NA
LC.03.0363	199.23175	NA
LC.03.0364	199.83275	NA
LC.03.0365	180.8735	NA
LC.03.0369	272.71225	NA
LC.03.0370	286.82	Phenylpropanoids and polyketides
LC.03.0372	347.814625	NA
LC.03.0373	22.79942857	NA
LC.03.0374	23.14685714	NA
LC.03.0375	23.13985714	Lipids and lipid-like molecules
LC.03.0376	23.32542857	NA

Compound	Retention Time	Super class
LC.03.0377	22.75528571	Organoheterocyclic compounds
LC.03.0378	17.943	Organic oxygen compounds
LC.03.0379	46.71114286	NA
LC.03.0380	596.7442857	Lipids and lipid-like molecules
LC.03.0381	577.3551429	Lipids and lipid-like molecules
LC.03.0382	631.6404286	NA
LC.03.0383	630.8931429	Phenylpropanoids and polyketides
LC.03.0384	631.9091429	NA
LC.03.0385	628.1182857	Lipids and lipid-like molecules
LC.03.0386	624.766	NA
LC.03.0387	600.9855714	NA
LC.03.0388	608.69	NA
LC.03.0389	664.5968571	Lipids and lipid-like molecules
LC.03.0390	848.9338571	NA
LC.03.0391	861.9571429	Lipids and lipid-like molecules
LC.03.0392	857.6711429	NA
LC.03.0393	859.2584286	Lipids and lipid-like molecules
LC.03.0394	711.2688571	NA
LC.03.0395	712.813	NA
LC.03.0396	721.7582857	Lipids and lipid-like molecules
LC.03.0397	674.4045714	Benzenoids
LC.03.0398	681.2037143	Lipids and lipid-like molecules
LC.03.0400	697.0745714	NA
LC.03.0402	692.469	Lipids and lipid-like molecules
LC.03.0403	726.8544286	Lipids and lipid-like molecules
LC.03.0404	746.3941429	Lipids and lipid-like molecules
LC.03.0405	746.0428571	Organic acids and derivatives

Compound	Retention Time	Super class
LC.03.0406	735.6632857	Lipids and lipid-like molecules
LC.03.0408	796.1501429	NA
LC.03.0409	796.7842857	NA
LC.03.0410	757.8942857	Phenylpropanoids and polyketides
LC.03.0411	755.6172857	Lipids and lipid-like molecules
LC.03.0412	770.853	Lipids and lipid-like molecules
LC.03.0413	784.2265714	NA
LC.03.0414	785.573	Lipids and lipid-like molecules
LC.03.0415	780.9108571	Lipids and lipid-like molecules
LC.03.0416	779.2102857	Organoheterocyclic compounds
LC.03.0417	373.7687143	Organoheterocyclic compounds
LC.03.0418	396.1874286	NA
LC.03.0419	395.0228571	NA
LC.03.0420	404.5392857	NA
LC.03.0421	413.1582857	Lipids and lipid-like molecules
LC.03.0422	497.0507143	Lipids and lipid-like molecules
LC.03.0423	500.34	Lipids and lipid-like molecules
LC.03.0424	541.6601429	Lipids and lipid-like molecules
LC.03.0426	186.4548571	NA
LC.03.0427	186.0041429	NA
LC.03.0428	189.554	NA
LC.03.0429	193.3357143	NA
LC.03.0430	196.2865714	NA
LC.03.0431	200.6652857	NA
LC.03.0432	197.6215714	NA
LC.03.0434	261.365	NA
LC.03.0435	229.0274286	NA

Compound	Retention Time	Super class
LC.03.0436	219.3641429	NA
LC.03.0437	315.2612857	Phenylpropanoids and polyketides
LC.03.0438	298.4435714	Lipids and lipid-like molecules
LC.03.0439	335.6967143	NA
LC.03.0440	343.3422857	Lipids and lipid-like molecules
LC.03.0441	32.142	Phenylpropanoids and polyketides
LC.03.0442	34.78916667	NA
LC.03.0443	21.70466667	Phenylpropanoids and polyketides
LC.03.0444	22.98566667	NA
LC.03.0445	22.98466667	NA
LC.03.0446	17.04016667	NA
LC.03.0447	18.14533333	Organoheterocyclic compounds
LC.03.0448	60.046	NA
LC.03.0449	44.28066667	NA
LC.03.0450	595.7781667	NA
LC.03.0451	583.288	Lipids and lipid-like molecules
LC.03.0452	577.3768333	Organoheterocyclic compounds
LC.03.0453	574.7131667	NA
LC.03.0454	632.6543333	NA
LC.03.0456	626.937	NA
LC.03.0457	623.8405	NA
LC.03.0458	623.5641667	Lipids and lipid-like molecules
LC.03.0459	623.7191667	NA
LC.03.0460	604.8513333	Lipids and lipid-like molecules
LC.03.0461	645.6496667	NA
LC.03.0462	645.5255	NA
LC.03.0463	870.9551667	NA

Compound	Retention Time	Super class
LC.03.0464	896.0383333	Lipids and lipid-like molecules
LC.03.0465	821.4775	Lipids and lipid-like molecules
LC.03.0466	815.0978333	NA
LC.03.0469	848.8095	NA
LC.03.0471	866.3981667	NA
LC.03.0472	855.2743333	Lipids and lipid-like molecules
LC.03.0473	700.7153333	NA
LC.03.0474	710.3183333	Lipids and lipid-like molecules
LC.03.0475	720.51	Lipids and lipid-like molecules
LC.03.0476	717.5561667	Lipids and lipid-like molecules
LC.03.0477	718.9123333	Lipids and lipid-like molecules
LC.03.0478	674.19	Lipids and lipid-like molecules
LC.03.0479	679.9371667	NA
LC.03.0480	695.9811667	NA
LC.03.0481	692.927	NA
LC.03.0482	690.1338333	NA
LC.03.0483	722.8616667	NA
LC.03.0484	731.6405	NA
LC.03.0485	745.6671667	NA
LC.03.0486	744.8145	NA
LC.03.0487	744.2225	NA
LC.03.0488	748.4273333	Lipids and lipid-like molecules
LC.03.0489	748.9246667	Organic oxygen compounds
LC.03.0490	738.5878333	Organic oxygen compounds
LC.03.0491	802.4348333	NA
LC.03.0492	800.7626667	NA
LC.03.0493	765.187	NA

Compound	Retention Time	Super class
LC.03.0494	764.4735	NA
LC.03.0495	790.4328333	Lipids and lipid-like molecules
LC.03.0496	787.5468333	NA
LC.03.0497	789.3603333	Lipids and lipid-like molecules
LC.03.0498	767.7416667	NA
LC.03.0499	768.9703333	Lipids and lipid-like molecules
LC.03.0500	784.3068333	Organic oxygen compounds
LC.03.0501	785.5315	Lipids and lipid-like molecules
LC.03.0502	781.832	Lipids and lipid-like molecules
LC.03.0503	781.1201667	Lipids and lipid-like molecules
LC.03.0504	779.9603333	Lipids and lipid-like molecules
LC.03.0505	779.3933333	Lipids and lipid-like molecules
LC.03.0506	780.9278333	Lipids and lipid-like molecules
LC.03.0507	387.5458333	Lipids and lipid-like molecules
LC.03.0508	409.483	Phenylpropanoids and polyketides
LC.03.0509	429.8105	Lipids and lipid-like molecules
LC.03.0510	444.7783333	NA
LC.03.0511	459.3028333	Lipids and lipid-like molecules
LC.03.0512	494.0485	Phenylpropanoids and polyketides
LC.03.0513	493.433	NA
LC.03.0515	479.1803333	NA
LC.03.0516	481.7378333	Lipids and lipid-like molecules
LC.03.0517	509.58	NA
LC.03.0518	523.6296667	NA
LC.03.0519	531.8875	NA
LC.03.0520	561.1721667	NA
LC.03.0521	558.5926667	Lipids and lipid-like molecules

Compound	Retention Time	Super class
LC.03.0522	563.2918333	Lipids and lipid-like molecules
LC.03.0523	194.3438333	NA
LC.03.0524	178.4958333	NA
LC.03.0525	175.4705	NA
LC.03.0526	170.0255	Organoheterocyclic compounds
LC.03.0527	161.8415	NA
LC.03.0529	257.4003333	Benzenoids
LC.03.0530	269.1216667	Phenylpropanoids and polyketides
LC.03.0531	276.028	Benzenoids
LC.03.0532	240.3298333	NA
LC.03.0533	246.9325	NA
LC.03.0534	311.6365	Organic acids and derivatives
LC.03.0535	357.369	NA
LC.03.0536	333.5505	Benzenoids
LC.03.0537	333.9526667	Lipids and lipid-like molecules
LC.03.0538	331.3646667	Organoheterocyclic compounds
LC.03.0539	1002.354	Phenylpropanoids and polyketides
LC.03.0540	71.0132	Organoheterocyclic compounds
LC.03.0541	70.7738	Organoheterocyclic compounds
LC.03.0542	32.7344	NA
LC.03.0543	33.6744	Nucleosides, nucleotides, and analogues
LC.03.0544	23.5142	Organoheterocyclic compounds
LC.03.0545	23.589	NA
LC.03.0546	25.3556	NA
LC.03.0547	23.223	NA
LC.03.0548	21.7178	Benzenoids
LC.03.0549	52.1256	NA

Compound	Retention Time	Super class
LC.03.0550	46.8744	Organic acids and derivatives
LC.03.0551	588.2316	NA
LC.03.0552	575.0152	NA
LC.03.0553	568.7996	Lipids and lipid-like molecules
LC.03.0555	617.4708	NA
LC.03.0556	623.815	NA
LC.03.0557	668.2492	NA
LC.03.0558	649.0706	NA
LC.03.0559	883.1052	NA
LC.03.0560	881.3402	NA
LC.03.0561	882.3758	Lipids and lipid-like molecules
LC.03.0562	872.2074	NA
LC.03.0564	901.0276	Organic oxygen compounds
LC.03.0565	815.6816	NA
LC.03.0566	817.3788	NA
LC.03.0567	814.2602	Lipids and lipid-like molecules
LC.03.0569	842.9766	NA
LC.03.0570	846.5042	NA
LC.03.0571	849.53	NA
LC.03.0573	860.5482	Organic oxygen compounds
LC.03.0574	868.9736	Lipids and lipid-like molecules
LC.03.0575	857.815	NA
LC.03.0576	856.7972	NA
LC.03.0577	859.3522	NA
LC.03.0578	852.7774	NA
LC.03.0579	851.9562	NA
LC.03.0580	704.5222	NA

Compound	Retention Time	Super class
LC.03.0581	716.9544	NA
LC.03.0582	718.3982	NA
LC.03.0583	681.6978	Lipids and lipid-like molecules
LC.03.0585	683.4006	NA
LC.03.0586	698.133	NA
LC.03.0587	694.997	Lipids and lipid-like molecules
LC.03.0589	725.2816	Lipids and lipid-like molecules
LC.03.0590	732.664	NA
LC.03.0591	746.8584	Lipids and lipid-like molecules
LC.03.0592	751.517	Lipids and lipid-like molecules
LC.03.0593	750.2308	NA
LC.03.0594	738.1194	Organic acids and derivatives
LC.03.0595	796.4674	NA
LC.03.0596	799.2546	Lipids and lipid-like molecules
LC.03.0597	755.2304	NA
LC.03.0598	788.9484	Lipids and lipid-like molecules
LC.03.0599	789.6946	NA
LC.03.0600	791.474	Lipids and lipid-like molecules
LC.03.0601	793.116	NA
LC.03.0602	774.2984	NA
LC.03.0603	782.634	Lipids and lipid-like molecules
LC.03.0604	382.7472	NA
LC.03.0605	388.1226	NA
LC.03.0606	372.9346	NA
LC.03.0607	396.643	NA
LC.03.0608	394.1066	Organoheterocyclic compounds
LC.03.0609	402.1658	NA

Compound	Retention Time	Super class
LC.03.0610	438.1418	NA
LC.03.0611	446.3924	NA
LC.03.0612	444.1152	Lipids and lipid-like molecules
LC.03.0613	456.3678	Organic acids and derivatives
LC.03.0614	486.3364	NA
LC.03.0615	490.3114	Organoheterocyclic compounds
LC.03.0616	488.1836	NA
LC.03.0617	467.128	Lipids and lipid-like molecules
LC.03.0618	467.8018	NA
LC.03.0619	473.7856	NA
LC.03.0621	480.5684	NA
LC.03.0622	508.581	NA
LC.03.0623	545	Lipids and lipid-like molecules
LC.03.0624	533.911	Lipids and lipid-like molecules
LC.03.0625	532.4618	Lipids and lipid-like molecules
LC.03.0626	555.338	NA
LC.03.0627	551.055	Alkaloids and derivatives
LC.03.0628	187.5498	NA
LC.03.0629	185.7932	NA
LC.03.0630	185.646	NA
LC.03.0631	190.4132	NA
LC.03.0632	200.5026	NA
LC.03.0633	202.6474	NA
LC.03.0634	179.0494	NA
LC.03.0635	170.8746	NA
LC.03.0636	268.9506	NA
LC.03.0637	226.9856	Benzenoids

Compound	Retention Time	Super class
LC.03.0638	217.8296	NA
LC.03.0639	235.098	NA
LC.03.0640	291.7698	Phenylpropanoids and polyketides
LC.03.0641	292.1006	NA
LC.03.0642	349.0844	Organoheterocyclic compounds
LC.03.0643	339.354	NA
LC.03.0644	1001.6088	NA
LC.03.0645	1000.3658	NA
LC.03.0646	36.74925	NA
LC.03.0647	25.151	Benzenoids
LC.03.0648	26.99225	Organic acids and derivatives
LC.03.0649	20.6485	Organoheterocyclic compounds
LC.03.0650	23.3565	NA
LC.03.0651	23.39425	NA
LC.03.0652	22.884	NA
LC.03.0653	22.60875	Organic acids and derivatives
LC.03.0654	22.64025	NA
LC.03.0655	23.60125	NA
LC.03.0656	18.57275	Organic nitrogen compounds
LC.03.0657	20.92475	NA
LC.03.0658	45.06525	NA
LC.03.0659	910.24525	NA
LC.03.0660	915.9385	Organic oxygen compounds
LC.03.0661	916.394	NA
LC.03.0662	594.7685	Lipids and lipid-like molecules
LC.03.0663	587.92825	NA
LC.03.0664	574.606	Lipids and lipid-like molecules

Compound	Retention Time	Super class
LC.03.0665	572.772	NA
LC.03.0666	618.418	Phenylpropanoids and polyketides
LC.03.0667	618.06475	Lipids and lipid-like molecules
LC.03.0669	666.8545	NA
LC.03.0670	664.69075	NA
LC.03.0671	652.0005	Lipids and lipid-like molecules
LC.03.0672	890.0625	Lipids and lipid-like molecules
LC.03.0673	895.731	NA
LC.03.0675	818.897	Lipids and lipid-like molecules
LC.03.0676	818.78225	NA
LC.03.0677	821.4725	NA
LC.03.0678	819.73925	Lipids and lipid-like molecules
LC.03.0679	820.60325	Lipids and lipid-like molecules
LC.03.0680	813.4305	NA
LC.03.0682	827.429	Lipids and lipid-like molecules
LC.03.0684	826.81125	NA
LC.03.0685	829.36475	NA
LC.03.0688	840.9385	NA
LC.03.0689	836.59	NA
LC.03.0690	836.316	Lipids and lipid-like molecules
LC.03.0691	849.03875	NA
LC.03.0692	846.6175	NA
LC.03.0699	856.6255	NA
LC.03.0700	852.8615	Lipids and lipid-like molecules
LC.03.0701	854.6445	NA
LC.03.0703	703.11775	Lipids and lipid-like molecules
LC.03.0704	704.6665	Lipids and lipid-like molecules

Compound	Retention Time	Super class
LC.03.0705	714.4545	NA
LC.03.0706	708.07375	NA
LC.03.0707	681.17075	NA
LC.03.0710	695.799	NA
LC.03.0711	695.96425	Lipids and lipid-like molecules
LC.03.0712	689.43725	Lipids and lipid-like molecules
LC.03.0713	687.1315	NA
LC.03.0715	731.0715	NA
LC.03.0716	731.852	Lipids and lipid-like molecules
LC.03.0717	741.95875	NA
LC.03.0718	807.289	NA
LC.03.0719	804.0045	Lipids and lipid-like molecules
LC.03.0720	807.5665	NA
LC.03.0721	796.05675	NA
LC.03.0722	801.93475	NA
LC.03.0723	798.68475	NA
LC.03.0724	797.99	NA
LC.03.0726	763.931	NA
LC.03.0727	764.292	NA
LC.03.0729	758.3795	Lipids and lipid-like molecules
LC.03.0731	794.6735	Lipids and lipid-like molecules
LC.03.0733	792.84125	NA
LC.03.0735	771.36175	Lipids and lipid-like molecules
LC.03.0736	769.40525	Lipids and lipid-like molecules
LC.03.0737	785.6155	NA
LC.03.0738	784.18475	Organoheterocyclic compounds
LC.03.0739	784.632	NA

Compound	Retention Time	Super class
LC.03.0740	783.08175	NA
LC.03.0741	783.249	NA
LC.03.0742	783.004	Lipids and lipid-like molecules
LC.03.0743	780.70525	Lipids and lipid-like molecules
LC.03.0744	781.16775	Lipids and lipid-like molecules
LC.03.0745	781.58325	NA
LC.03.0747	776.23	NA
LC.03.0748	776.16775	NA
LC.03.0749	386.251	NA
LC.03.0750	380.299	NA
LC.03.0751	436.605	NA
LC.03.0752	430.3315	Phenylpropanoids and polyketides
LC.03.0753	441.33325	NA
LC.03.0754	452.07675	NA
LC.03.0755	491.713	NA
LC.03.0756	503.49675	NA
LC.03.0757	497.13825	Organosulfur compounds
LC.03.0758	496.22625	Lipids and lipid-like molecules
LC.03.0759	464.767	Lipids and lipid-like molecules
LC.03.0760	466.53175	Organic acids and derivatives
LC.03.0761	468.223	NA
LC.03.0763	474.64	NA
LC.03.0764	483.3625	NA
LC.03.0765	481.10175	NA
LC.03.0766	507.1525	Lipids and lipid-like molecules
LC.03.0767	527.91425	Lipids and lipid-like molecules
LC.03.0768	518.288	NA

Compound	Retention Time	Super class
LC.03.0769	515.4305	NA
LC.03.0770	545.04175	NA
LC.03.0771	542.52775	Organic nitrogen compounds
LC.03.0772	533.27375	Lipids and lipid-like molecules
LC.03.0773	555.11625	NA
LC.03.0774	188.82575	NA
LC.03.0775	190.82975	NA
LC.03.0776	183.00325	Organic oxygen compounds
LC.03.0777	196.9085	NA
LC.03.0778	195.75125	NA
LC.03.0779	199.632	NA
LC.03.0780	204.1685	Phenylpropanoids and polyketides
LC.03.0781	172.03625	NA
LC.03.0782	173.3625	NA
LC.03.0783	173.3055	NA
LC.03.0784	158.83275	NA
LC.03.0785	262.905	NA
LC.03.0786	209.588	NA
LC.03.0787	212.72075	NA
LC.03.0788	232.749	NA
LC.03.0789	247.705	NA
LC.03.0790	248.1525	NA
LC.03.0791	285.29625	NA
LC.03.0792	325.7125	NA
LC.03.0793	318.903	NA
LC.03.0794	322.2685	NA
LC.03.0795	359.8215	NA

Compound	Retention Time	Super class
LC.03.0796	358.236	Phenylpropanoids and polyketides
LC.03.0797	357.68325	NA
LC.03.0798	355.8645	NA
LC.03.0799	341.9315	NA
LC.03.0800	29.20033333	Organoheterocyclic compounds
LC.03.0802	38.388	NA
LC.03.0803	22.476	NA
LC.03.0804	23.244	Lipids and lipid-like molecules
LC.03.0805	22.74366667	NA
LC.03.0807	25.69366667	NA
LC.03.0808	15.82533333	Organic nitrogen compounds
LC.03.0809	20.841	NA
LC.03.0810	51.952	Organoheterocyclic compounds
LC.03.0811	47.90366667	Organoheterocyclic compounds
LC.03.0812	939.398	NA
LC.03.0813	597.176	NA
LC.03.0814	592.7416667	NA
LC.03.0815	598.4526667	NA
LC.03.0816	596.6913333	Lipids and lipid-like molecules
LC.03.0817	582.369	NA
LC.03.0819	635.6103333	Organoheterocyclic compounds
LC.03.0820	621.5803333	NA
LC.03.0821	600.3233333	NA
LC.03.0822	611.7276667	NA
LC.03.0824	670.207	NA
LC.03.0825	667.2703333	Organic acids and derivatives
LC.03.0826	665.2546667	Lipids and lipid-like molecules

Compound	Retention Time	Super class
LC.03.0827	654.3936667	NA
LC.03.0828	653.9963333	Lipids and lipid-like molecules
LC.03.0829	654.5133333	Organic nitrogen compounds
LC.03.0830	880.5906667	NA
LC.03.0831	885.658	NA
LC.03.0832	902.959	NA
LC.03.0835	839.4	Lipids and lipid-like molecules
LC.03.0837	843.5086667	Lipids and lipid-like molecules
LC.03.0838	832.0226667	NA
LC.03.0839	844.1853333	NA
LC.03.0841	865.908	NA
LC.03.0843	850.8576667	Lipids and lipid-like molecules
LC.03.0844	702.0336667	Lipids and lipid-like molecules
LC.03.0845	701.864	NA
LC.03.0846	700.8206667	Lipids and lipid-like molecules
LC.03.0847	706.5383333	NA
LC.03.0848	704.296	Lipids and lipid-like molecules
LC.03.0851	713.0363333	NA
LC.03.0852	709.203	NA
LC.03.0853	678.146	NA
LC.03.0855	683.4766667	NA
LC.03.0856	697.5783333	NA
LC.03.0857	696.1626667	NA
LC.03.0858	724.6746667	NA
LC.03.0859	746.0456667	NA
LC.03.0860	752.4106667	NA
LC.03.0861	738.0886667	NA

Compound	Retention Time	Super class
LC.03.0862	737.2293333	Lipids and lipid-like molecules
LC.03.0864	806.9423333	Lipids and lipid-like molecules
LC.03.0865	799.6286667	NA
LC.03.0866	765.8376667	Lipids and lipid-like molecules
LC.03.0867	764.788	NA
LC.03.0868	754.721	Lipids and lipid-like molecules
LC.03.0869	793.18	NA
LC.03.0871	789.5896667	NA
LC.03.0873	791.66	Lipids and lipid-like molecules
LC.03.0874	767.6676667	Lipids and lipid-like molecules
LC.03.0875	778.2546667	Organic oxygen compounds
LC.03.0876	397.3666667	NA
LC.03.0877	403.396	NA
LC.03.0879	420.6233333	NA
LC.03.0880	434.657	Organic nitrogen compounds
LC.03.0882	447.7456667	NA
LC.03.0883	441.0543333	NA
LC.03.0884	453.5506667	NA
LC.03.0885	457.691	NA
LC.03.0887	504.0563333	Lipids and lipid-like molecules
LC.03.0888	464.4466667	Lipids and lipid-like molecules
LC.03.0889	468.8346667	Organoheterocyclic compounds
LC.03.0890	476.6913333	NA
LC.03.0891	472.991	Lipids and lipid-like molecules
LC.03.0893	527.4226667	NA
LC.03.0895	513.3593333	NA
LC.03.0896	520.702	Organic acids and derivatives

Compound	Retention Time	Super class
LC.03.0898	542.8566667	Lipids and lipid-like molecules
LC.03.0899	537.4096667	NA
LC.03.0900	536.7043333	NA
LC.03.0901	553.9076667	NA
LC.03.0903	188.2733333	NA
LC.03.0904	183.0856667	NA
LC.03.0906	194.424	NA
LC.03.0907	195.6446667	Organoheterocyclic compounds
LC.03.0908	190.2623333	NA
LC.03.0909	192.8403333	Lipids and lipid-like molecules
LC.03.0910	200.6213333	NA
LC.03.0911	199.1	Organic acids and derivatives
LC.03.0913	206.1103333	Organoheterocyclic compounds
LC.03.0914	152.6073333	NA
LC.03.0915	175.4146667	NA
LC.03.0916	168.749	NA
LC.03.0917	169.5416667	Phenylpropanoids and polyketides
LC.03.0918	172.9333333	NA
LC.03.0919	166.023	NA
LC.03.0921	162.2853333	Benzenoids
LC.03.0922	262.89	NA
LC.03.0923	279.3156667	NA
LC.03.0925	276.969	NA
LC.03.0926	223.5236667	Phenylpropanoids and polyketides
LC.03.0928	210.005	NA
LC.03.0929	216.242	NA
LC.03.0930	216.8366667	NA

Compound	Retention Time	Super class
LC.03.0931	291.8236667	Phenylpropanoids and polyketides
LC.03.0932	314.179	NA
LC.03.0933	317.783	Lipids and lipid-like molecules
LC.03.0934	301.6493333	Lipids and lipid-like molecules
LC.03.0935	334.0303333	Lipids and lipid-like molecules
LC.03.0936	348.1033333	NA
LC.03.0937	336.8936667	Lipids and lipid-like molecules
LC.03.0939	1005.009333	NA
LC.03.0940	1005.115333	NA
LC.03.0941	87.8145	NA
LC.03.0942	30.4685	Lipids and lipid-like molecules
LC.03.0943	30.5075	Organic acids and derivatives
LC.03.0944	25.8475	Organoheterocyclic compounds
LC.03.0945	21.355	Organic acids and derivatives
LC.03.0946	22.587	Organic acids and derivatives
LC.03.0948	21.666	Alkaloids and derivatives
LC.03.0950	23.2375	NA
LC.03.0951	23.4905	Organic oxygen compounds
LC.03.0952	23.727	Organic acids and derivatives
LC.03.0953	21.776	NA
LC.03.0954	23.127	NA
LC.03.0955	23.159	NA
LC.03.0956	23.488	NA
LC.03.0957	23.197	Benzenoids
LC.03.0958	23.4935	NA
LC.03.0960	23.549	NA
LC.03.0961	23.3635	NA

Compound	Retention Time	Super class
LC.03.0962	23.625	NA
LC.03.0963	23.604	NA
LC.03.0964	23.5915	NA
LC.03.0965	22.776	Lipids and lipid-like molecules
LC.03.0966	15.773	Organic oxygen compounds
LC.03.0967	17.9655	NA
LC.03.0968	19.952	NA
LC.03.0969	20.343	Organic oxygen compounds
LC.03.0970	60.2475	Organosulfur compounds
LC.03.0971	60.45	NA
LC.03.0972	51.2545	Organic oxygen compounds
LC.03.0973	50.6215	NA
LC.03.0974	50.9185	NA
LC.03.0975	47.123	NA
LC.03.0976	43.055	NA
LC.03.0977	44.047	NA
LC.03.0978	960.656	Organic acids and derivatives
LC.03.0979	932.5805	NA
LC.03.0980	595.092	NA
LC.03.0981	588.077	Alkaloids and derivatives
LC.03.0982	585.4445	NA
LC.03.0983	583.235	NA
LC.03.0984	579.135	Lipids and lipid-like molecules
LC.03.0987	578.3475	NA
LC.03.0988	574.6485	NA
LC.03.0989	573.823	NA
LC.03.0990	568.549	NA

Compound	Retention Time	Super class
LC.03.0993	636.085	NA
LC.03.0994	632.076	NA
LC.03.0995	633.92	NA
LC.03.0996	630.461	NA
LC.03.0997	631.955	NA
LC.03.0998	628.0945	Lipids and lipid-like molecules
LC.03.0999	615.298	NA
LC.03.1000	620.8625	NA
LC.03.1001	620.2925	NA
LC.03.1002	626.7115	NA
LC.03.1003	624.8945	Lipids and lipid-like molecules
LC.03.1004	606.8615	NA
LC.03.1005	612.1665	Lipids and lipid-like molecules
LC.03.1006	610.2285	Organoheterocyclic compounds
LC.03.1007	659.4325	NA
LC.03.1008	658.3675	Lipids and lipid-like molecules
LC.03.1009	654.331	NA
LC.03.1011	648.772	Lipids and lipid-like molecules
LC.03.1012	884.8855	NA
LC.03.1013	904.425	NA
LC.03.1014	901.3335	NA
LC.03.1015	818.45	NA
LC.03.1017	849.408	NA
LC.03.1020	866.262	Lipids and lipid-like molecules
LC.03.1024	704.296	Lipids and lipid-like molecules
LC.03.1025	698.941	NA
LC.03.1026	699.5205	Lipids and lipid-like molecules

Compound	Retention Time	Super class
LC.03.1027	701.2495	Lipids and lipid-like molecules
LC.03.1028	712.2095	Lipids and lipid-like molecules
LC.03.1029	714.617	NA
LC.03.1030	714.54	Lipids and lipid-like molecules
LC.03.1031	713.3365	NA
LC.03.1032	706.3025	Lipids and lipid-like molecules
LC.03.1033	708.1225	NA
LC.03.1034	715.1735	NA
LC.03.1035	716.5895	NA
LC.03.1036	719.108	NA
LC.03.1037	718.661	NA
LC.03.1038	675.865	NA
LC.03.1039	675.1325	NA
LC.03.1040	675.802	Phenylpropanoids and polyketides
LC.03.1041	679.296	NA
LC.03.1042	680.976	NA
LC.03.1043	682.1705	Lipids and lipid-like molecules
LC.03.1044	679.2565	NA
LC.03.1045	682.962	NA
LC.03.1046	684.125	NA
LC.03.1047	686.423	Lipids and lipid-like molecules
LC.03.1048	697.518	NA
LC.03.1049	697.678	Benzenoids
LC.03.1050	695.439	Organic oxygen compounds
LC.03.1051	687.247	NA
LC.03.1052	690.985	NA
LC.03.1054	693.5605	NA

Compound	Retention Time	Super class
LC.03.1055	724.5825	NA
LC.03.1056	722.2435	NA
LC.03.1057	723.1745	NA
LC.03.1058	729.526	NA
LC.03.1059	727.241	NA
LC.03.1061	754.6835	Lipids and lipid-like molecules
LC.03.1062	753.267	Lipids and lipid-like molecules
LC.03.1063	743.3815	Lipids and lipid-like molecules
LC.03.1064	741.4755	Lipids and lipid-like molecules
LC.03.1067	737.257	NA
LC.03.1068	737.5755	NA
LC.03.1069	738.0475	NA
LC.03.1070	738.2635	Lipids and lipid-like molecules
LC.03.1072	808.806	Organoheterocyclic compounds
LC.03.1073	801.1915	NA
LC.03.1074	800.6985	NA
LC.03.1075	799.5075	NA
LC.03.1077	762.9835	NA
LC.03.1078	763.957	NA
LC.03.1080	763.9875	NA
LC.03.1081	766.304	NA
LC.03.1082	768.338	NA
LC.03.1083	759.5585	NA
LC.03.1084	758.181	NA
LC.03.1085	756.71	NA
LC.03.1088	789.8765	Lipids and lipid-like molecules
LC.03.1089	788.3565	NA

Compound	Retention Time	Super class
LC.03.1091	774.8045	NA
LC.03.1092	773.4965	NA
LC.03.1093	768.6365	NA
LC.03.1095	768.7545	Lipids and lipid-like molecules
LC.03.1096	787.108	NA
LC.03.1097	784.7615	NA
LC.03.1098	782.7555	Lipids and lipid-like molecules
LC.03.1099	783.193	Lipids and lipid-like molecules
LC.03.1100	776.4685	NA
LC.03.1101	779.817	Lipids and lipid-like molecules
LC.03.1102	781.38	NA
LC.03.1104	775.7515	Lipids and lipid-like molecules
LC.03.1105	382.6835	Lipids and lipid-like molecules
LC.03.1106	387.0005	NA
LC.03.1107	373.8905	NA
LC.03.1108	374.1725	NA
LC.03.1109	370.0775	Lipids and lipid-like molecules
LC.03.1110	379.9695	NA
LC.03.1111	379.4375	Lipids and lipid-like molecules
LC.03.1112	376.5455	NA
LC.03.1113	393.3525	NA
LC.03.1114	396.628	NA
LC.03.1115	397.3265	Lipids and lipid-like molecules
LC.03.1117	398.876	Organic acids and derivatives
LC.03.1118	398.865	Lipids and lipid-like molecules
LC.03.1119	401.5995	Benzenoids
LC.03.1120	402.1595	Lipids and lipid-like molecules

Compound	Retention Time	Super class
LC.03.1121	403.427	NA
LC.03.1122	405.708	NA
LC.03.1123	409.453	NA
LC.03.1124	408.466	NA
LC.03.1125	409.388	NA
LC.03.1126	408.0895	Lipids and lipid-like molecules
LC.03.1127	418.1325	NA
LC.03.1128	437.623	Organic oxygen compounds
LC.03.1129	436.6755	NA
LC.03.1131	446.842	NA
LC.03.1132	450.8115	NA
LC.03.1134	459.2015	Lipids and lipid-like molecules
LC.03.1135	494.8975	Organic nitrogen compounds
LC.03.1136	490.564	Lipids and lipid-like molecules
LC.03.1137	500.7425	NA
LC.03.1138	502.1015	NA
LC.03.1139	498.7805	NA
LC.03.1140	495.7435	NA
LC.03.1141	499.441	NA
LC.03.1142	464.966	Lipids and lipid-like molecules
LC.03.1143	464.7515	NA
LC.03.1144	465.8115	NA
LC.03.1145	468.91	Benzenoids
LC.03.1146	468.734	NA
LC.03.1147	477.128	Lipids and lipid-like molecules
LC.03.1149	472.3785	NA
LC.03.1150	473.3805	NA

Compound	Retention Time	Super class
LC.03.1151	473.9825	NA
LC.03.1152	484.5315	NA
LC.03.1153	484.812	NA
LC.03.1154	482.7455	NA
LC.03.1155	479.96	Lipids and lipid-like molecules
LC.03.1156	481.5715	Lipids and lipid-like molecules
LC.03.1157	480.912	Phenylpropanoids and polyketides
LC.03.1158	481.5995	NA
LC.03.1159	526.109	Organic oxygen compounds
LC.03.1160	521.448	Organic oxygen compounds
LC.03.1161	522.685	NA
LC.03.1162	522.3185	NA
LC.03.1163	542.7035	NA
LC.03.1164	537.8945	NA
LC.03.1165	560.0515	NA
LC.03.1166	562.251	NA
LC.03.1167	550.838	Organoheterocyclic compounds
LC.03.1168	549.9465	NA
LC.03.1170	184.6535	NA
LC.03.1171	194.419	NA
LC.03.1173	191.5155	Lipids and lipid-like molecules
LC.03.1174	201.1505	NA
LC.03.1175	204.392	NA
LC.03.1176	143.5615	Organoheterocyclic compounds
LC.03.1177	179.9045	NA
LC.03.1178	179.8385	NA
LC.03.1179	174.2925	NA

Compound	Retention Time	Super class
LC.03.1180	177.136	Phenylpropanoids and polyketides
LC.03.1181	176.0565	NA
LC.03.1183	168.849	Lipids and lipid-like molecules
LC.03.1184	159.6255	Phenylpropanoids and polyketides
LC.03.1185	261.0755	NA
LC.03.1186	272.1685	NA
LC.03.1187	271.541	Phenylpropanoids and polyketides
LC.03.1188	225.381	NA
LC.03.1189	226.758	NA
LC.03.1190	207.137	NA
LC.03.1191	209.72	Organoheterocyclic compounds
LC.03.1192	210.135	NA
LC.03.1193	211.5015	NA
LC.03.1194	211.052	Phenylpropanoids and polyketides
LC.03.1195	218.947	NA
LC.03.1196	219.192	NA
LC.03.1197	217.202	NA
LC.03.1198	241.5335	NA
LC.03.1199	238.747	NA
LC.03.1200	236.442	Lipids and lipid-like molecules
LC.03.1201	234.4845	Phenylpropanoids and polyketides
LC.03.1202	251.2975	NA
LC.03.1205	283.7015	NA
LC.03.1206	287.576	Organoheterocyclic compounds
LC.03.1207	328.0685	Organic oxygen compounds
LC.03.1208	327.295	NA
LC.03.1209	318.109	Organoheterocyclic compounds

Compound	Retention Time	Super class
LC.03.1210	321.9625	NA
LC.03.1211	323.8605	Phenylpropanoids and polyketides
LC.03.1212	307.442	Alkaloids and derivatives
LC.03.1213	304.673	NA
LC.03.1214	307.0595	NA
LC.03.1215	309.7015	Benzenoids
LC.03.1216	296.4415	NA
LC.03.1217	298.375	NA
LC.03.1219	300.225	NA
LC.03.1221	357.251	NA
LC.03.1222	355.1905	NA
LC.03.1223	362.9445	NA
LC.03.1224	352.9475	NA
LC.03.1225	338.739	NA
LC.03.1226	336.582	NA
LC.03.1227	340.461	Organic nitrogen compounds
LC.03.1228	342.3705	Hydrocarbons
LC.03.1229	1005.149	Organic acids and derivatives
LC.03.1231	1002.738	NA
LC.03.1232	1001.9225	NA
LC.03.1233	985.176	NA

Table S6 The enrichment test of lipids and lipid-like molecules among the 26 network modules identified by WGCNA based on metabolite annotation. The enrichment test was done with the Fisher's exact test.

	Network Module		All metabolites		Pvalue	Pvalue.adj
Module	Lipids-like	Non-Lipids-like	Lipids-like	Non-Lipids-like		
red	37	44	321	1373	9.51×10^{-8}	2.00×10^{-6}
tan	27	29	321	1373	1.16×10^{-6}	1.50×10^{-5}
pink	30	37	321	1373	2.21×10^{-6}	1.90×10^{-5}
orange	14	8	321	1373	6.37×10^{-6}	3.30×10^{-5}
green	34	49	321	1373	5.72×10^{-6}	3.30×10^{-5}
brown	39	73	321	1373	1.00×10^{-4}	4.33×10^{-4}
darkred	14	18	321	1373	1.30×10^{-3}	4.82×10^{-3}
yellow	31	75	321	1373	8.80×10^{-3}	2.86×10^{-2}
lightyellow	12	24	321	1373	0.03	0.09
midnightblue	14	32	321	1373	0.04	0.12
lightcyan	12	34	321	1373	0.15	0.34
darkgrey	7	17	321	1373	0.16	0.34
greenyellow	15	48	321	1373	0.21	0.42
royalblue	7	27	321	1373	0.47	0.88
black	0	76	321	1373	1.00	1.00
magenta	1	63	321	1373	1.00	1.00
grey	4	132	321	1373	1.00	1.00
darkgreen	0	31	321	1373	1.00	1.00
lightgreen	1	36	321	1373	1.00	1.00
purple	2	62	321	1373	1.00	1.00
turquoise	1	181	321	1373	1.00	1.00
blue	4	119	321	1373	1.00	1.00
grey60	5	37	321	1373	0.92	1.00

darkturquoise	1	29	321	1373	1.00	1.00
salmon	2	50	321	1373	1.00	1.00
cyan	7	42	321	1373	0.84	1.00

Lipids-like=Lipids and lipid-like molecules;

Non-Lipids-like= Non-Lipids and non-lipid-like molecules

Table S7 Percent changes in prediction accuracy of G+M over GBLUP(G) and metabolomic BLUP (M) models for the 17 traits in the Diversity panel. The first three columns are median values of prediction accuracies across 50 re-sampling runs for G, M and G+M models, respectively; the last two columns are percent changes in prediction accuracy of G+M over G and M models, respectively.

TraitName	G	M	G+M	GM_to_G (%)[a]	GM_to_M (%)[b]
Plant Height	0.598	0.539	0.660	10.4	11.5
Days to Heading	0.615	0.652	0.688	11.9	11.2
Seed Length	0.457	0.465	0.672	46.9	46.2
Seed Width	0.694	0.692	0.722	4.1	4.1
Seed Height	0.779	0.705	0.799	2.5	2.8
Hundred Kernel Weight	0.715	0.700	0.801	12.0	12.2
Hundred Hull Weight	0.395	0.436	0.560	41.7	37.8
Groat Percentage	0.545	0.534	0.622	14.2	14.5
C14:0	0.421	0.405	0.628	49.2	51.1
C16:0	0.524	0.803	0.840	60.4	39.4
C16:1	0.612	0.566	0.651	6.4	6.9
C18:0	0.501	0.784	0.839	67.3	43.1
C18:1	0.550	0.855	0.878	59.7	38.4
C18:2	0.580	0.805	0.844	45.4	32.7
C18:3	0.358	0.430	0.520	45.1	37.5
C20:0	0.525	0.843	0.848	61.4	38.3
C20:1	0.609	0.707	0.768	26.2	22.5

[a]percent changes in prediction accuracy of G+M over G model

[b]percent changes in prediction accuracy of G+M over M model
